# Supplementary material for: Human WDR5 promotes breast cancer growth and metastasis via KMT2-independent translation regulation
Source: eLife. 2022 Aug 31;11:e78163. doi: 10.7554/eLife.78163 (PMC9584608; doi:10.7554/eLife.78163)
Supplement: Figure 1—source data 1. [file elife-78163-fig1-data1.zip › Figure 1-source data 1/Figure 1-source data 1_labeled images.pptx]

## Slide 1
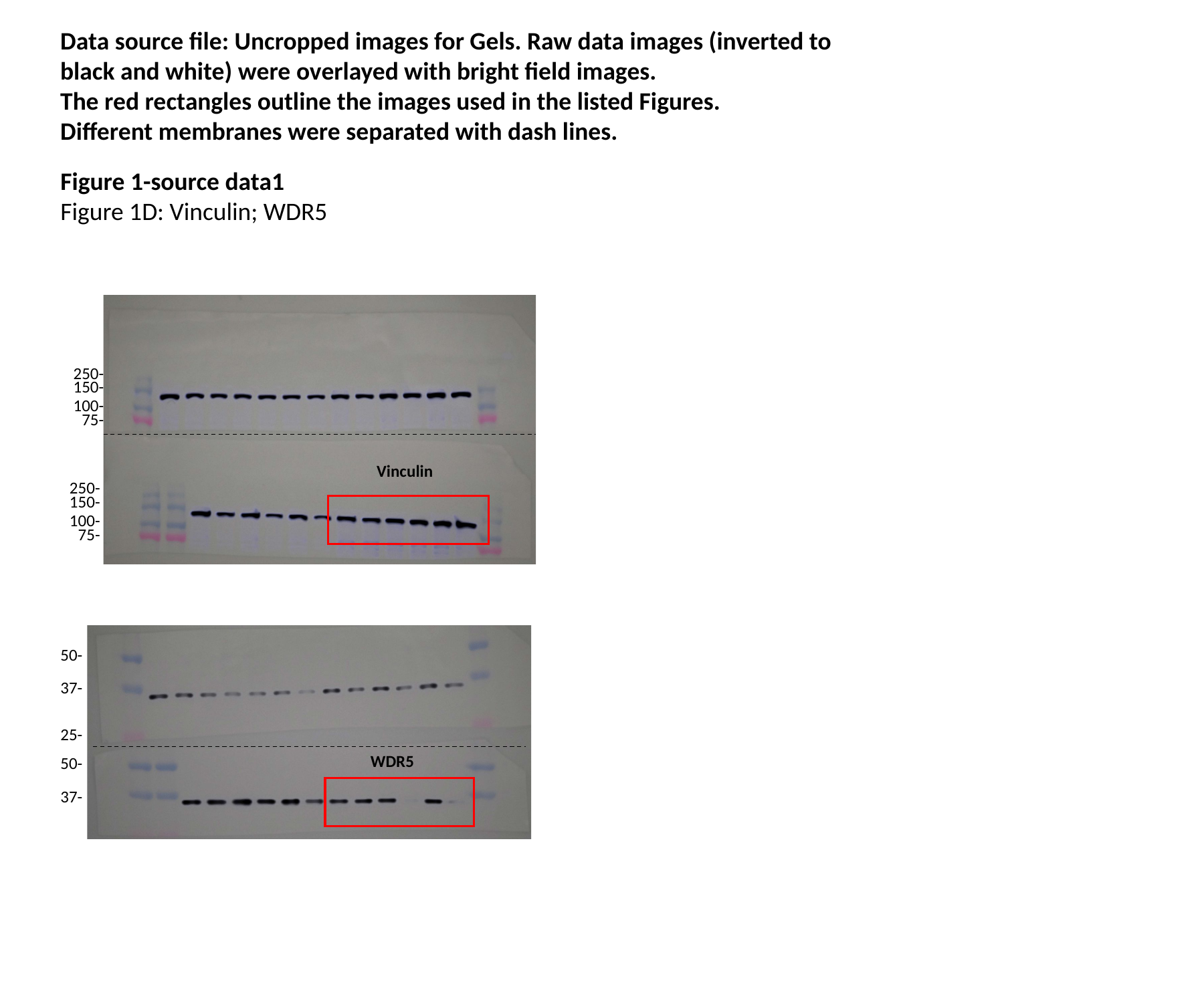

Data source file: Uncropped images for Gels. Raw data images (inverted to black and white) were overlayed with bright field images.
The red rectangles outline the images used in the listed Figures.
Different membranes were separated with dash lines.
Figure 1-source data1
Figure 1D: Vinculin; WDR5
250-
150-
100-
75-
Vinculin
250-
150-
100-
75-
50-
37-
25-
WDR5
50-
37-
