# Supplementary figures and images for: Human WDR5 promotes breast cancer growth and metastasis via KMT2-independent translation regulation (part 1 of 2)
Source: eLife. 2022 Aug 31;11:e78163. doi: 10.7554/eLife.78163 (PMC9584608; doi:10.7554/eLife.78163)

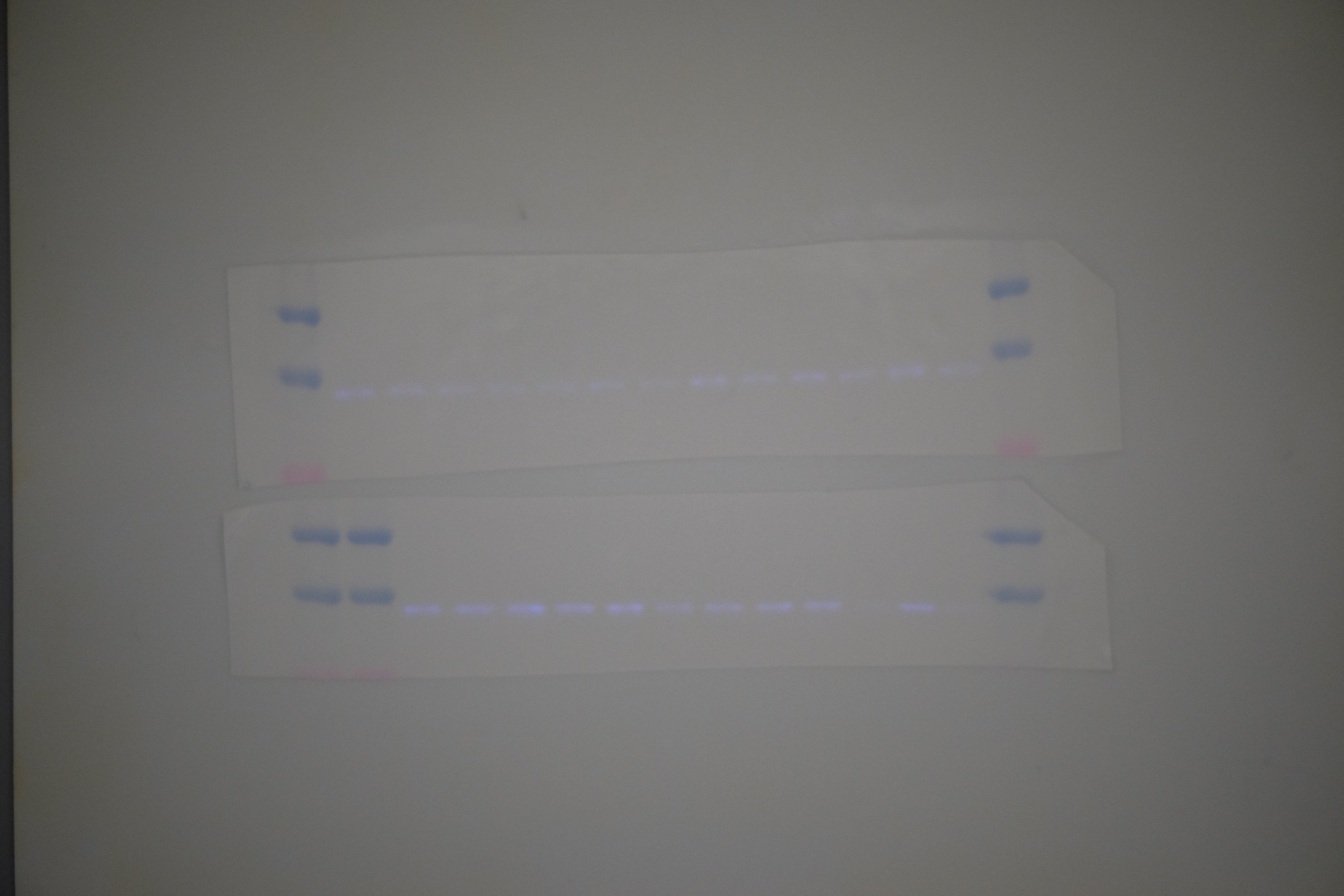

Supplement: Figure 1—source data 1. [file elife-78163-fig1-data1.zip › Figure 1-source data 1/DSCF9383.JPG]

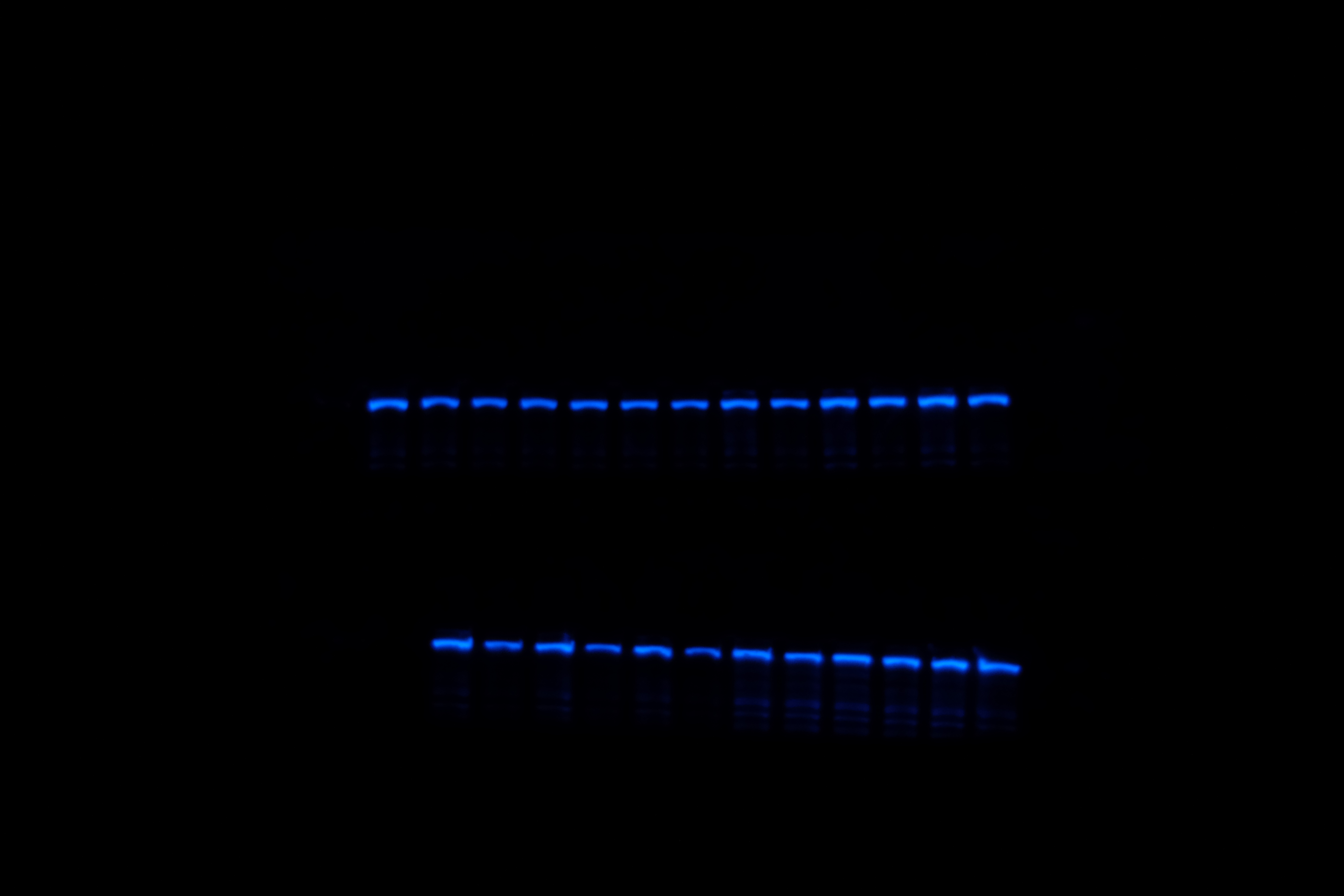

Supplement: Figure 1—source data 1. [file elife-78163-fig1-data1.zip › Figure 1-source data 1/Fig. 1D_Vinvulin.JPG]

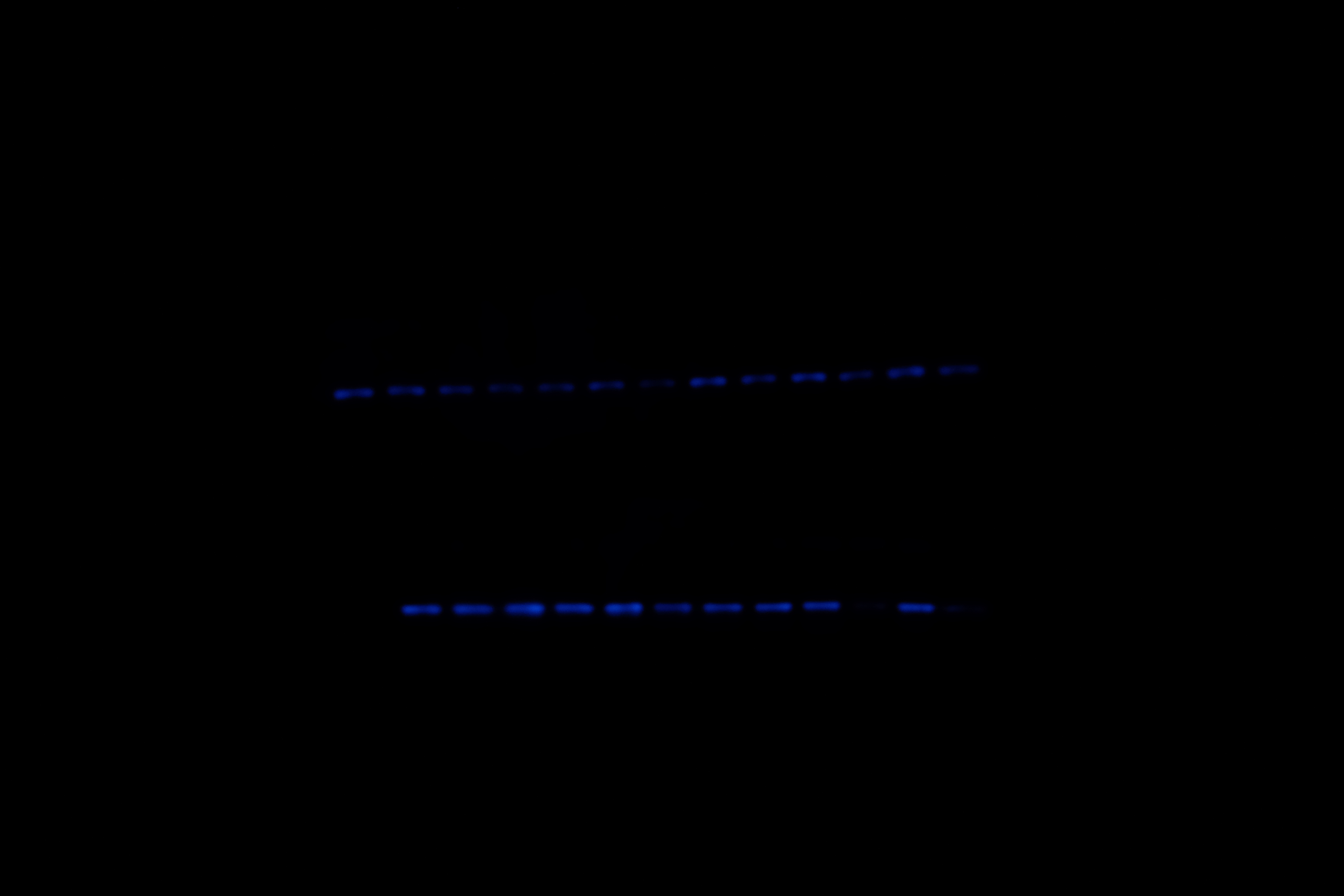

Supplement: Figure 1—source data 1. [file elife-78163-fig1-data1.zip › Figure 1-source data 1/Fig. 1D_WDR5.JPG]

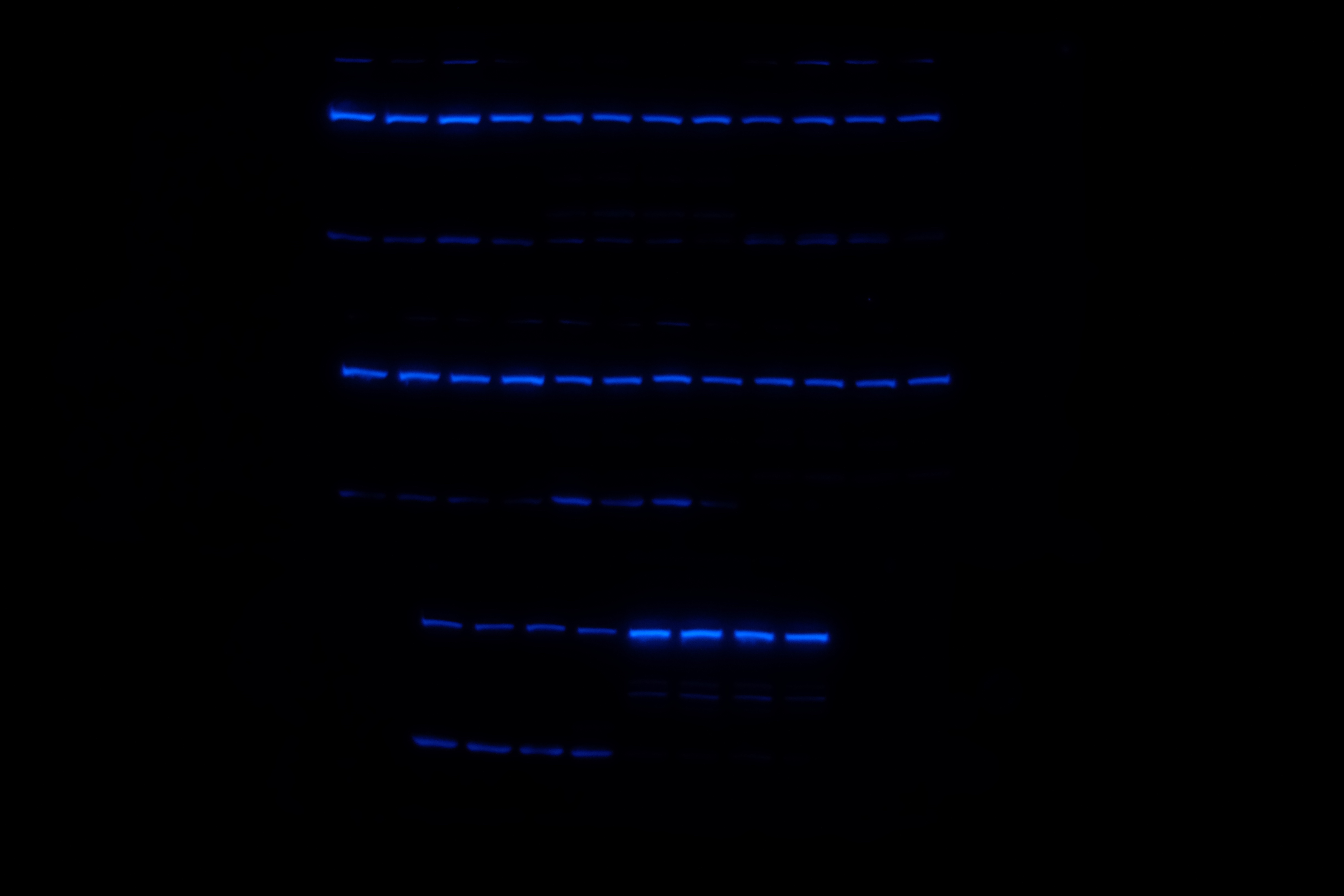

Supplement: Figure 2—source data 1. [file elife-78163-fig2-data1.zip › Figure 2-source data 1/F2A_vinculin.JPG]

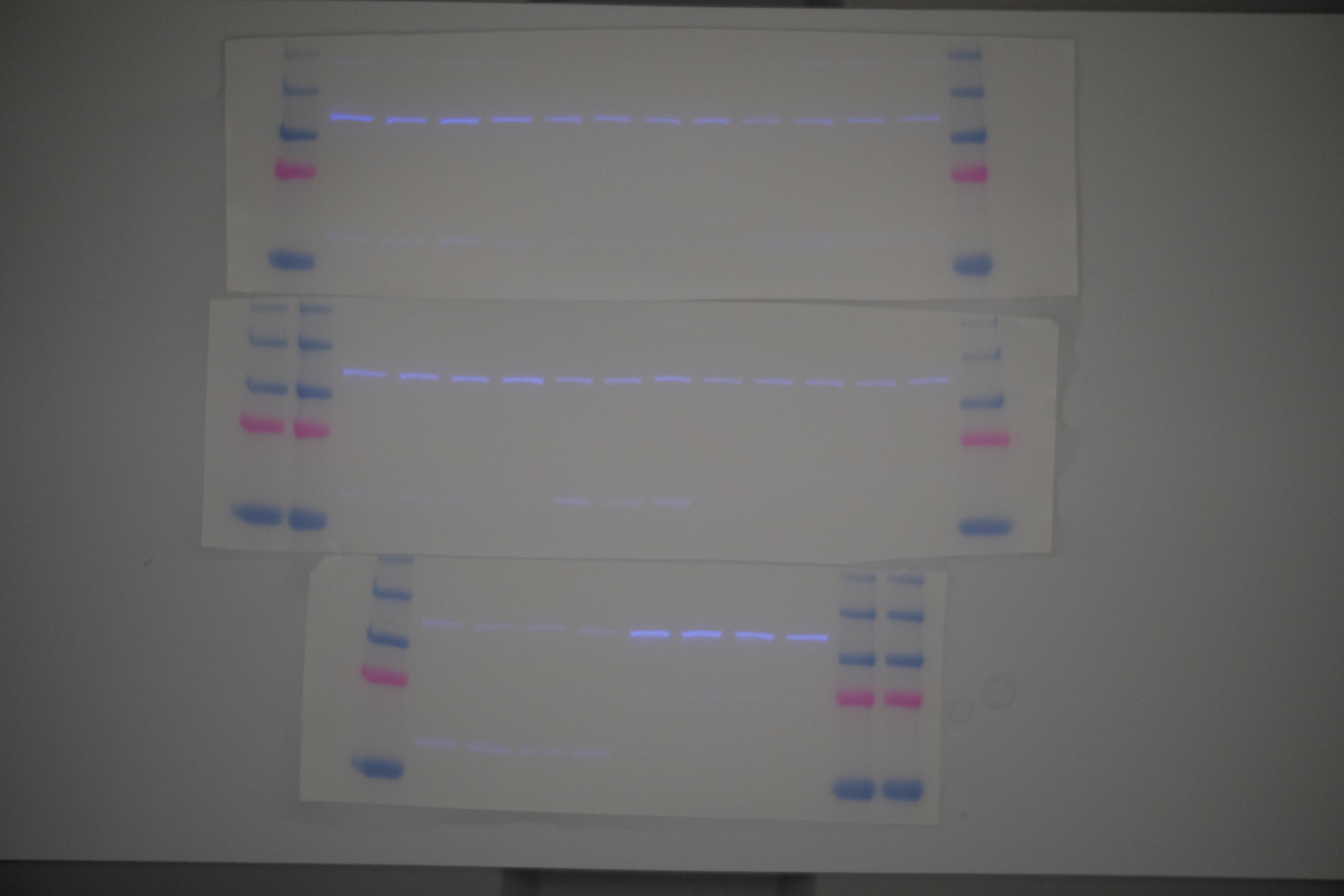

Supplement: Figure 2—source data 1. [file elife-78163-fig2-data1.zip › Figure 2-source data 1/F2A_vinculin_light.JPG]

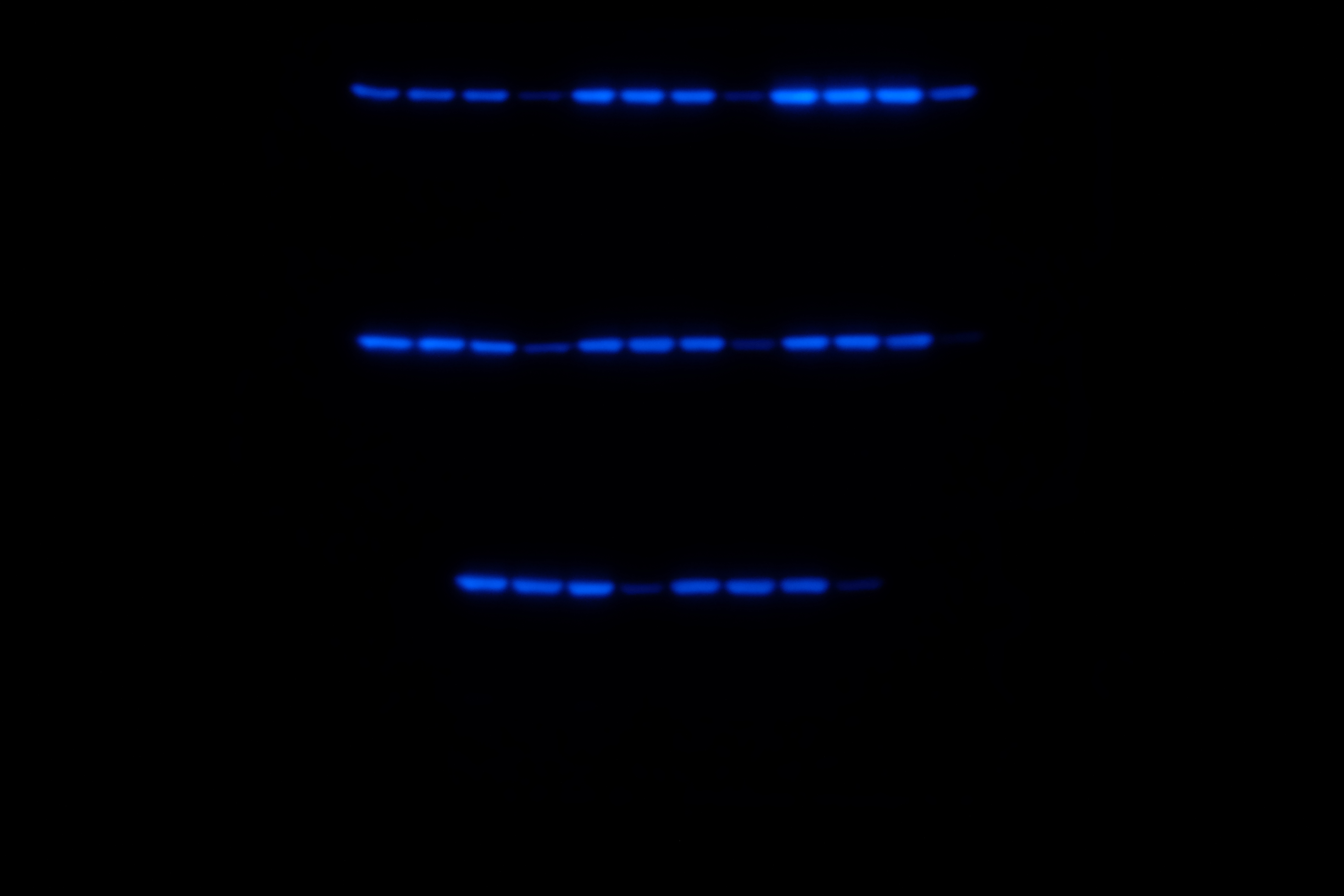

Supplement: Figure 2—source data 1. [file elife-78163-fig2-data1.zip › Figure 2-source data 1/F2A_WDR5_HCC1143_UACC812_MCF7_T47D_MDAMB231_UACC893_BT474_SKBR3.JPG]

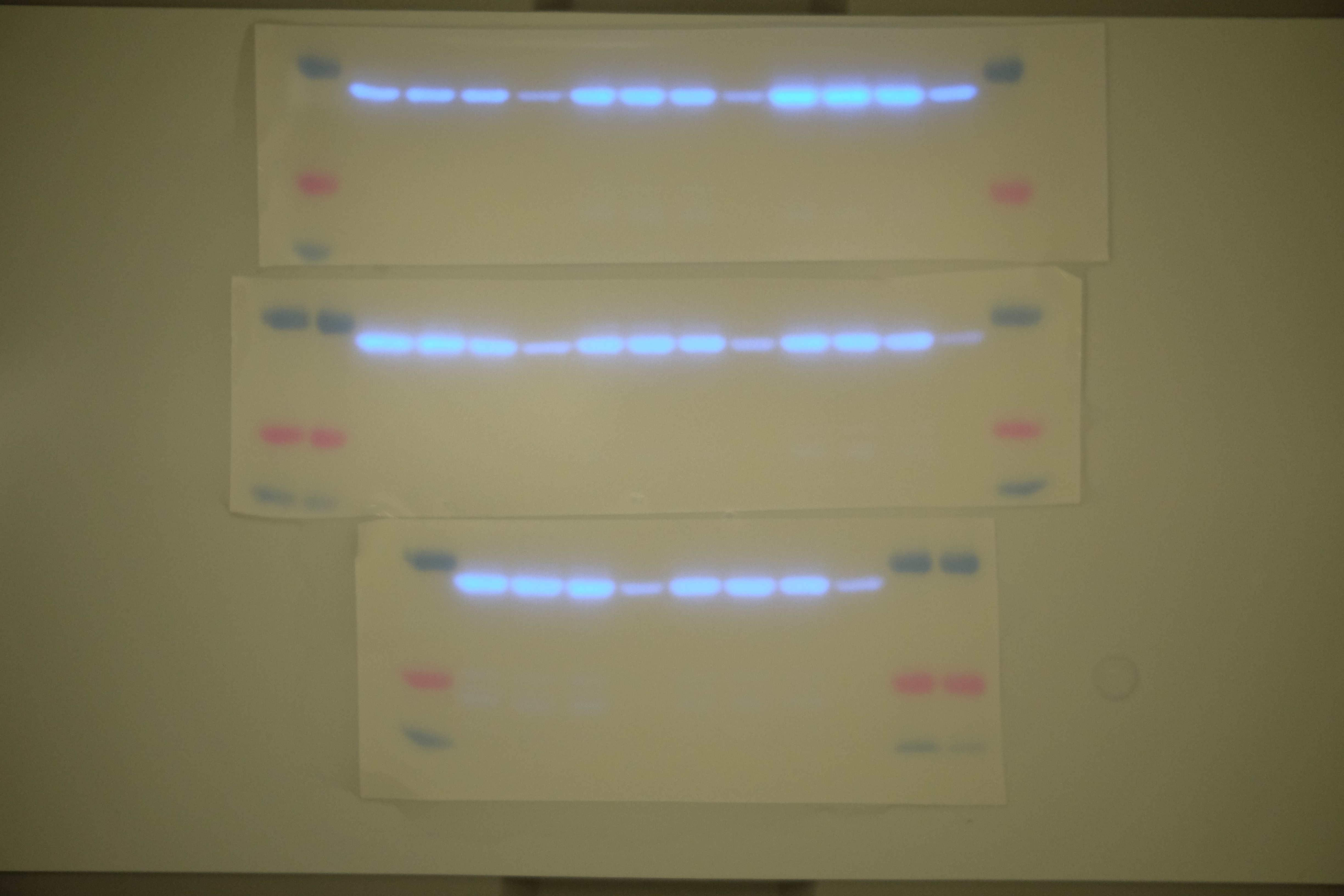

Supplement: Figure 2—source data 1. [file elife-78163-fig2-data1.zip › Figure 2-source data 1/F2A_WDR5_light.JPG]

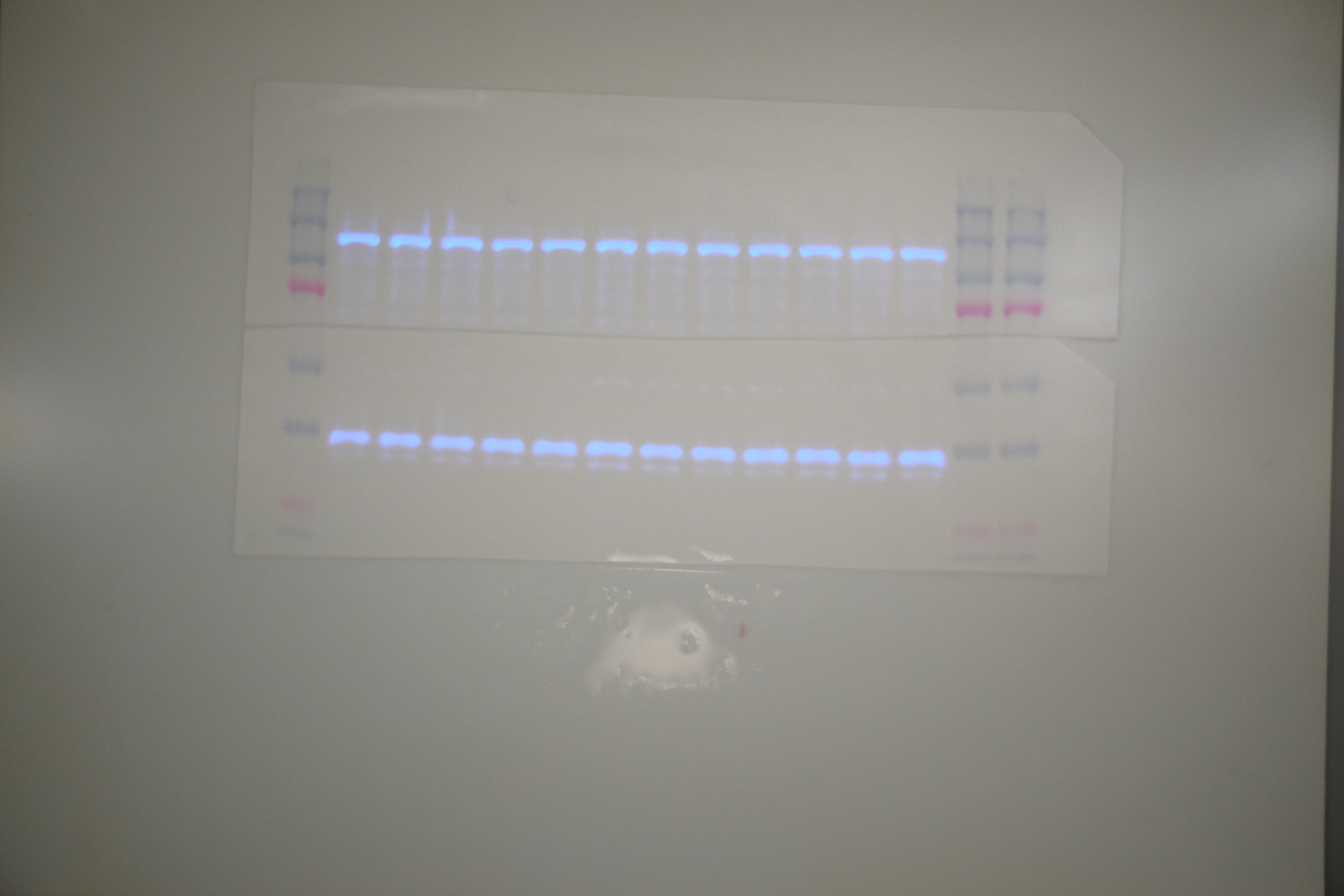

Supplement: Figure 2—source data 2. [file elife-78163-fig2-data2.zip › Figure 2-source data 2/DSCF4107.JPG]

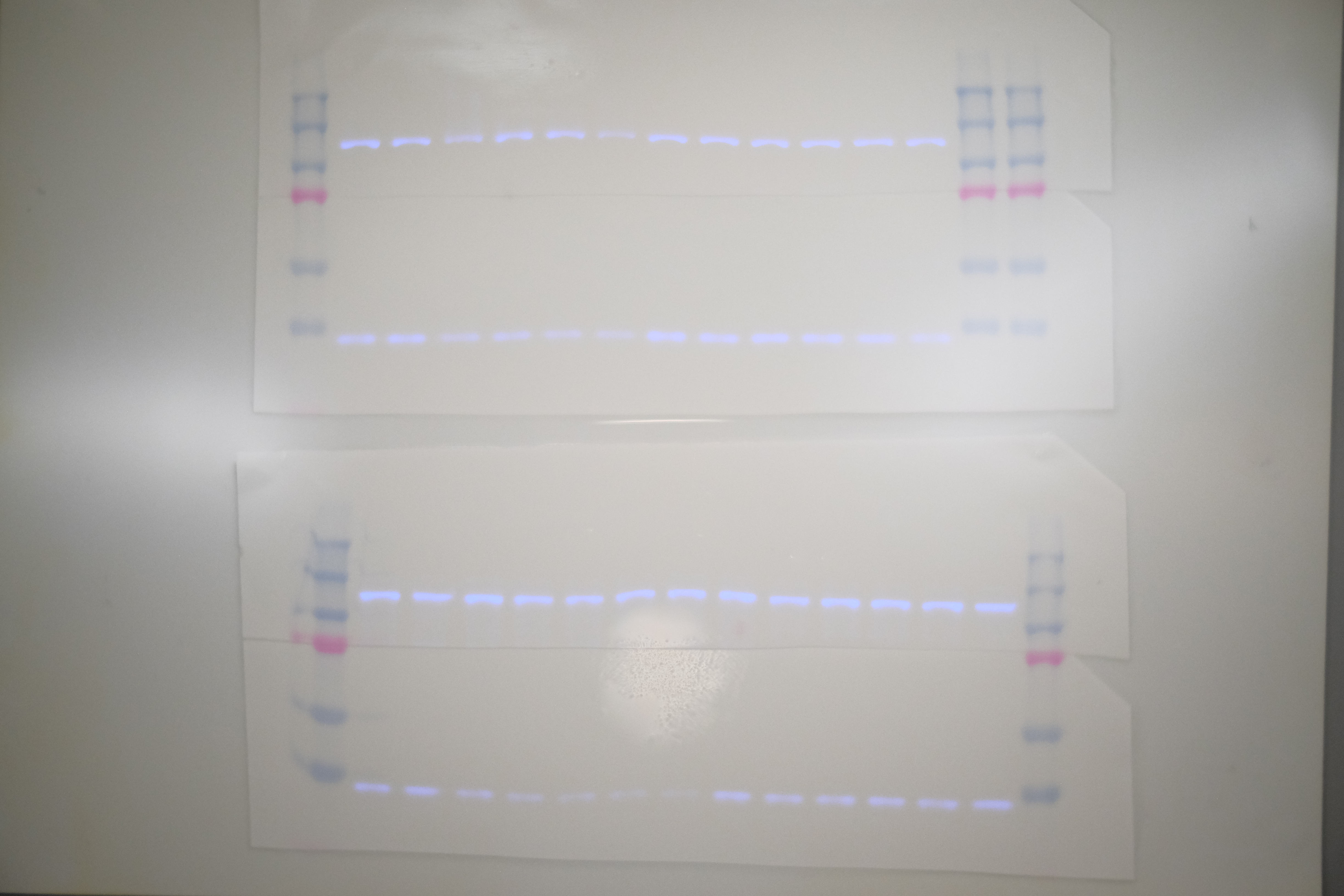

Supplement: Figure 2—source data 2. [file elife-78163-fig2-data2.zip › Figure 2-source data 2/DSCF4165.JPG]

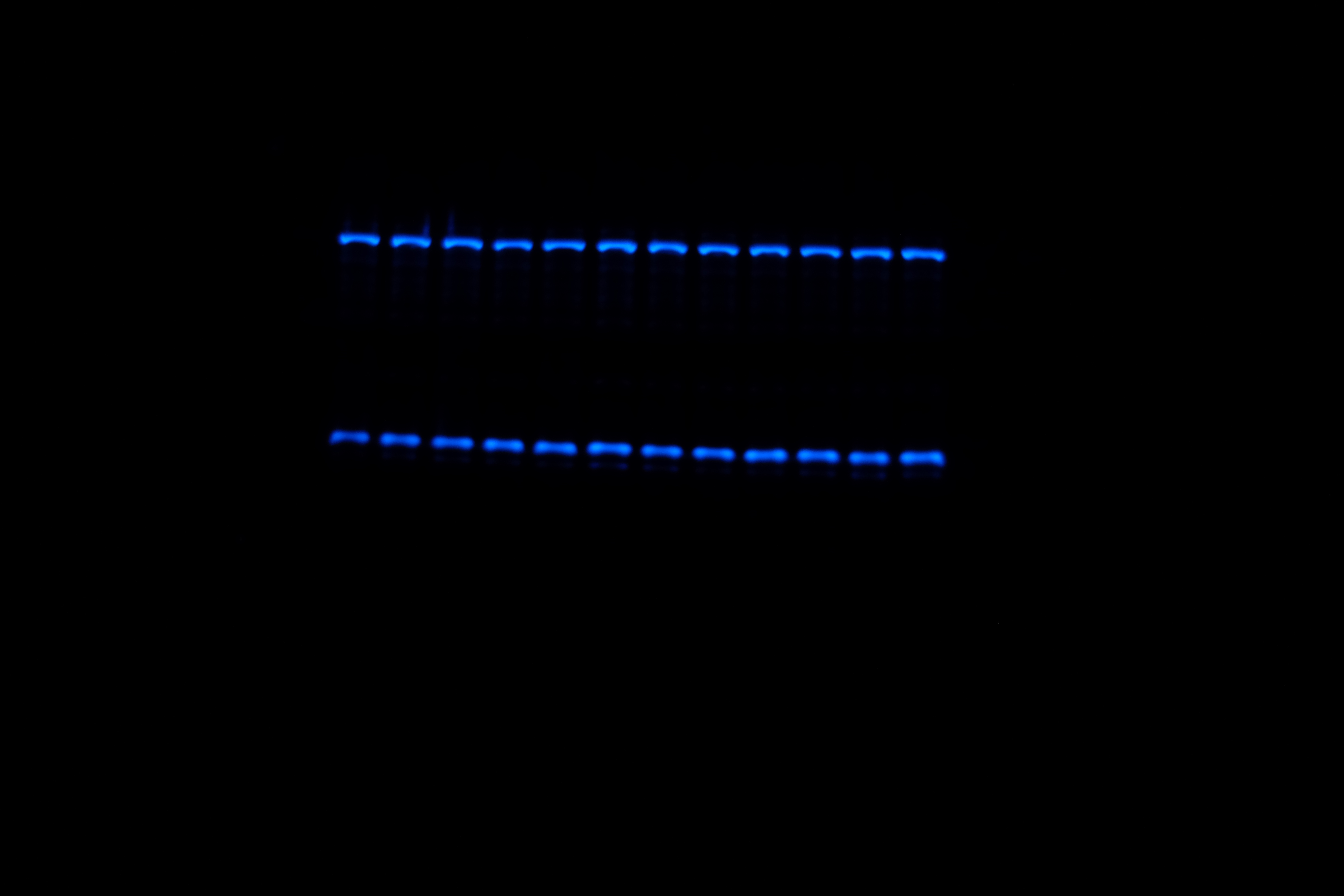

Supplement: Figure 2—source data 2. [file elife-78163-fig2-data2.zip › Figure 2-source data 2/Fig.2H_Vinculin_WDR5_MS67_MS67N.JPG]

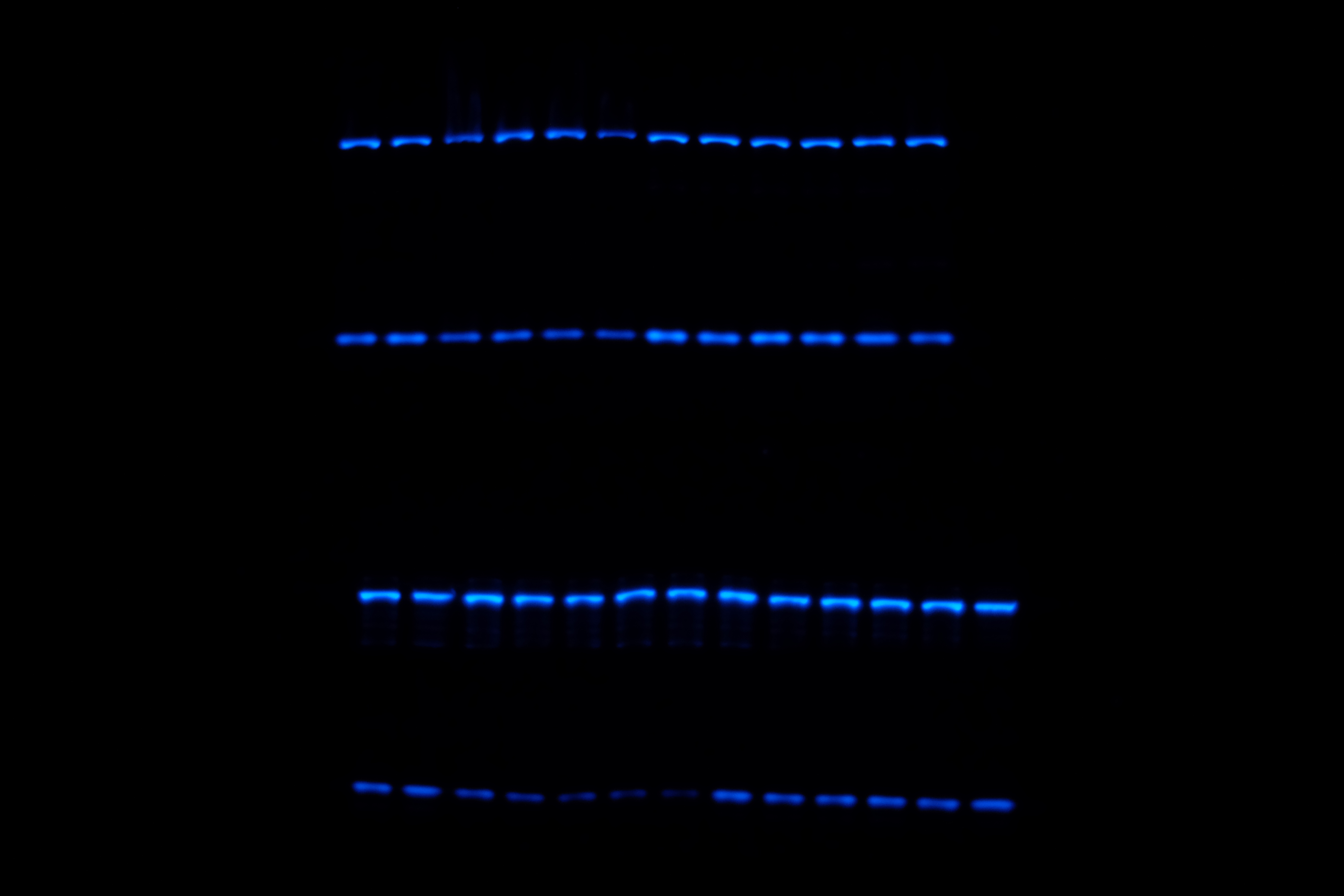

Supplement: Figure 2—source data 2. [file elife-78163-fig2-data2.zip › Figure 2-source data 2/Fig.2H_Vinculin_WDR5_OICR-9429.JPG]

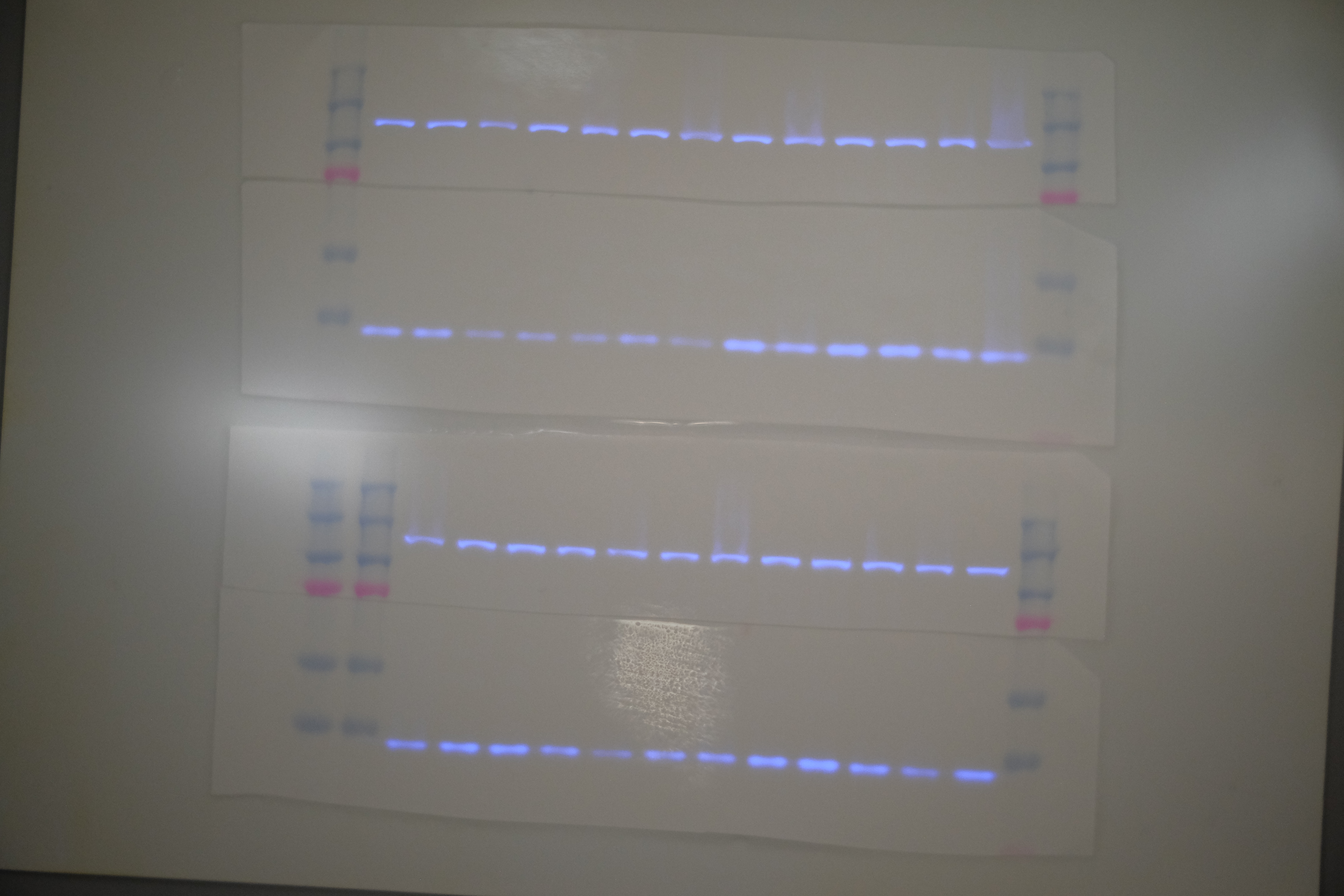

Supplement: Figure 2—figure supplement 1—source data 1. [file elife-78163-fig2-figsupp1-data1.zip › Figure 2-figure supplement 1-source data 1/DSCF4157.JPG]

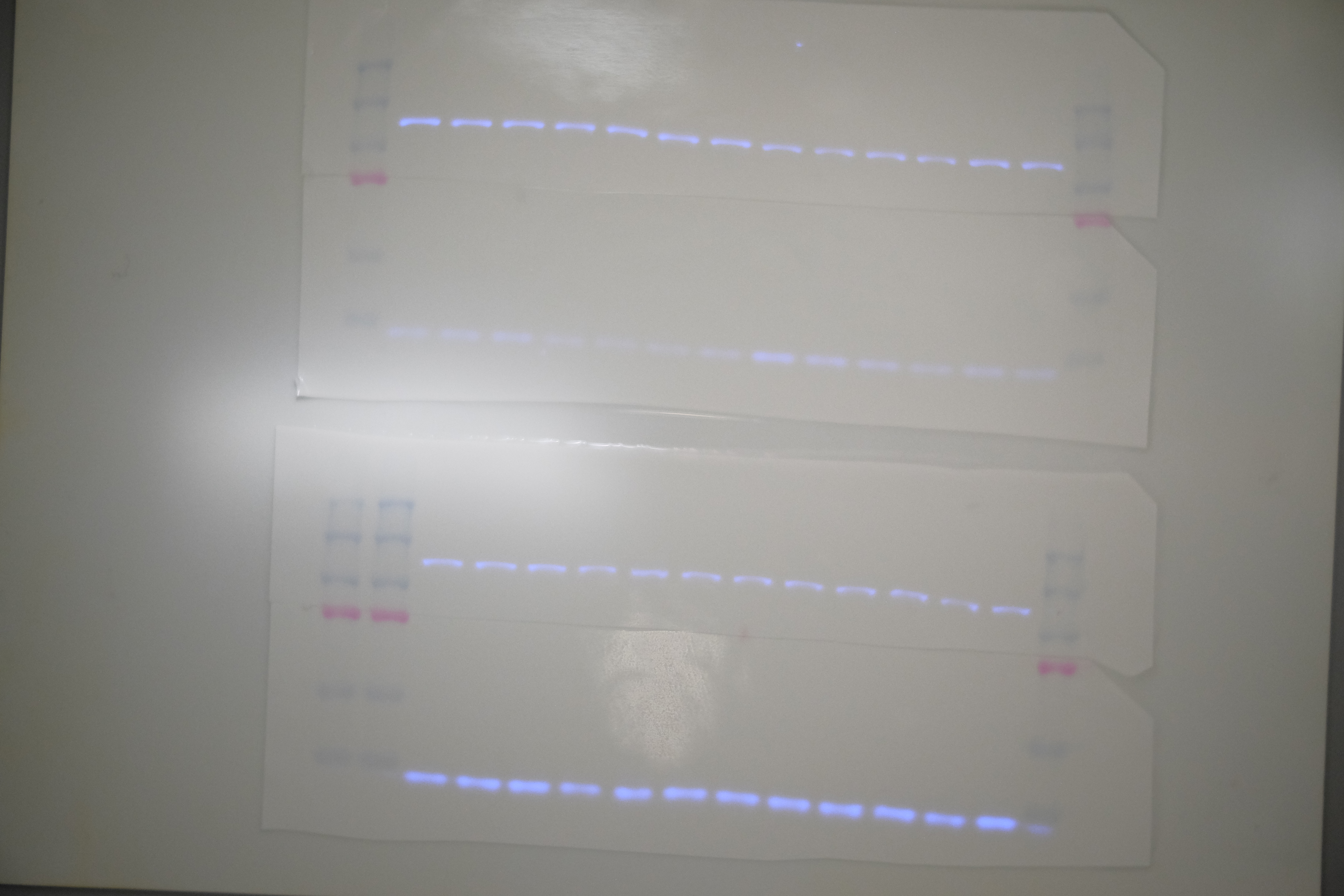

Supplement: Figure 2—figure supplement 1—source data 1. [file elife-78163-fig2-figsupp1-data1.zip › Figure 2-figure supplement 1-source data 1/DSCF4187.JPG]

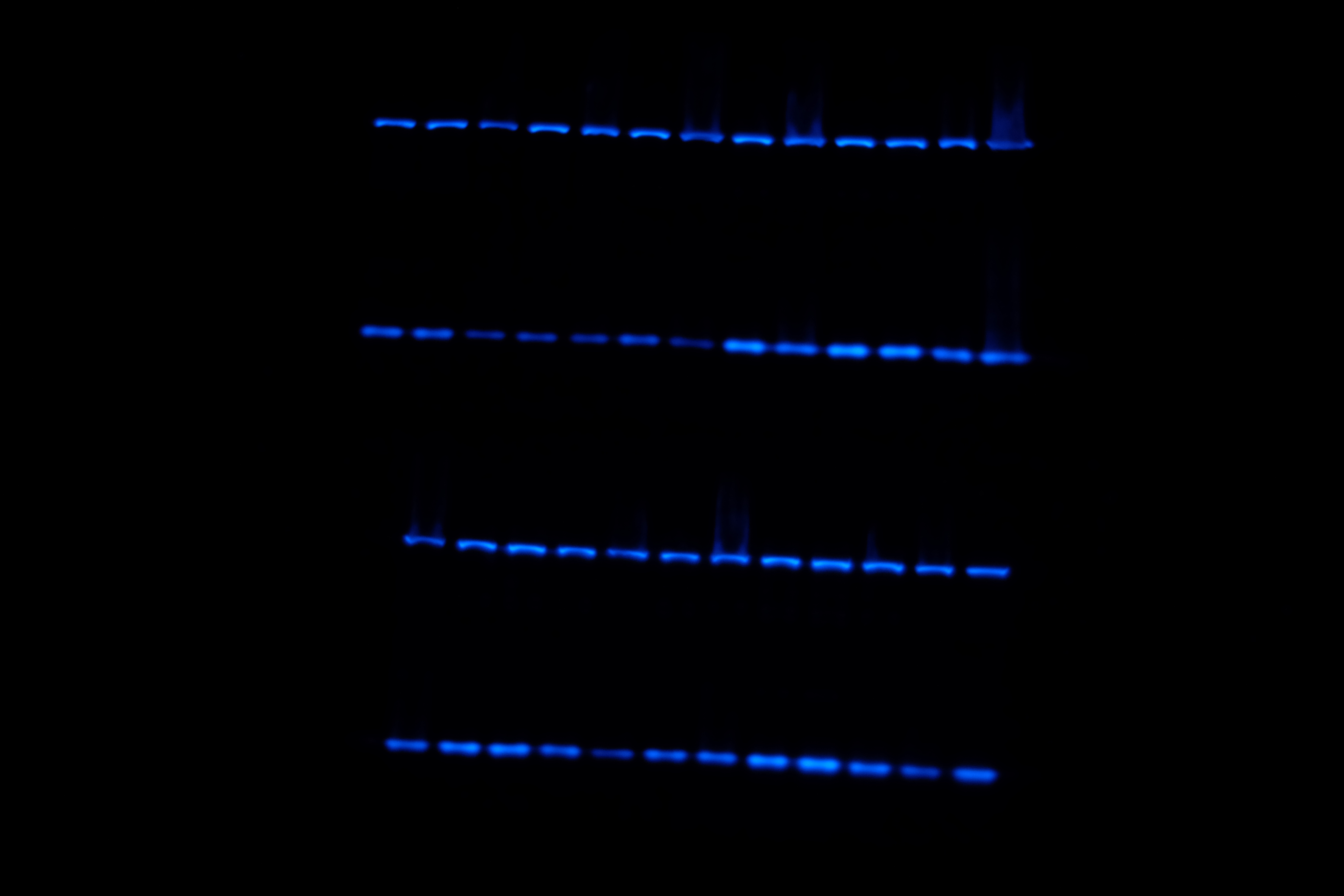

Supplement: Figure 2—figure supplement 1—source data 1. [file elife-78163-fig2-figsupp1-data1.zip › Figure 2-figure supplement 1-source data 1/Fig2-S1C_MS67N_WDR5_vinculin.JPG]

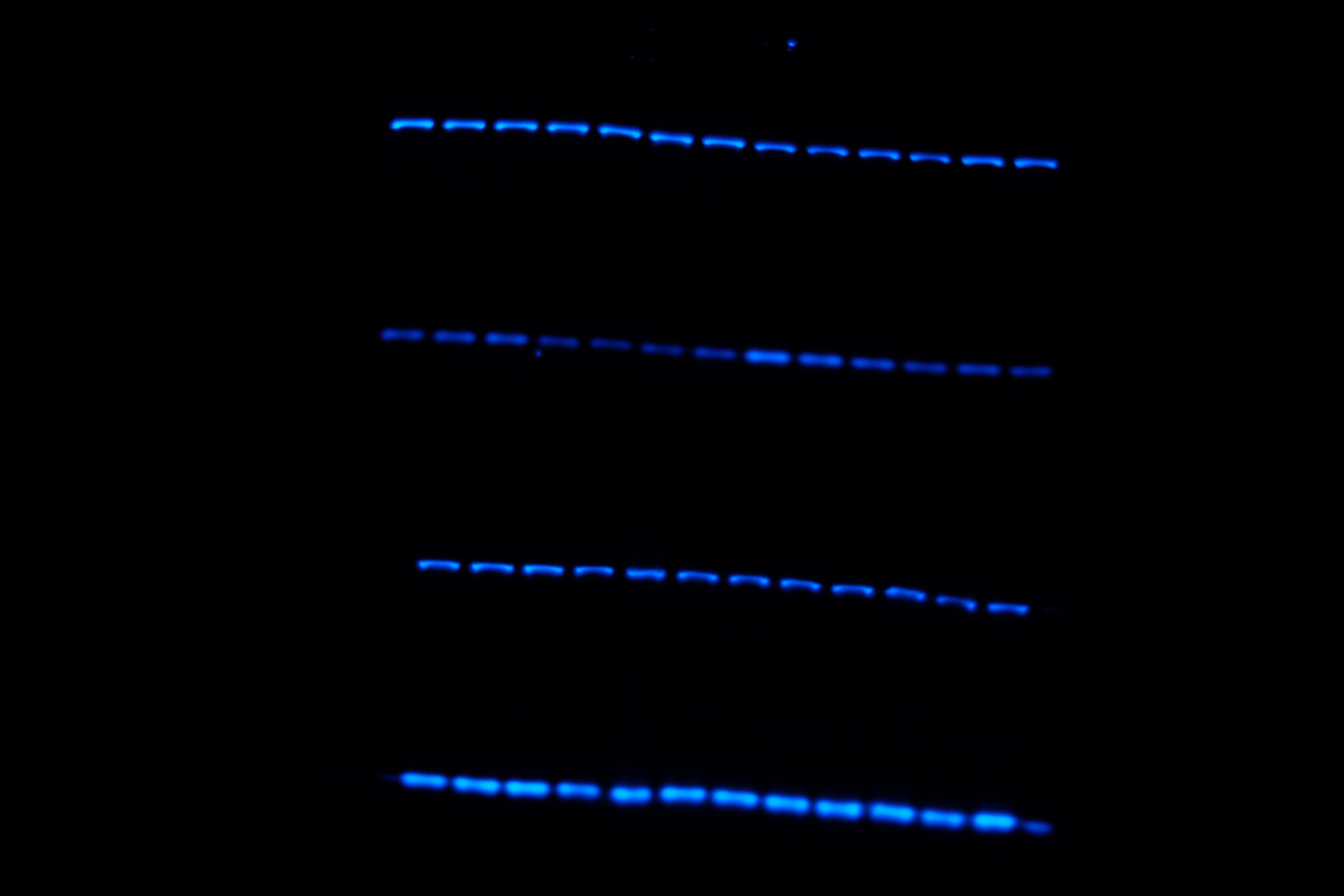

Supplement: Figure 2—figure supplement 1—source data 1. [file elife-78163-fig2-figsupp1-data1.zip › Figure 2-figure supplement 1-source data 1/Fig2-S1C_MS67_vinculin.JPG]

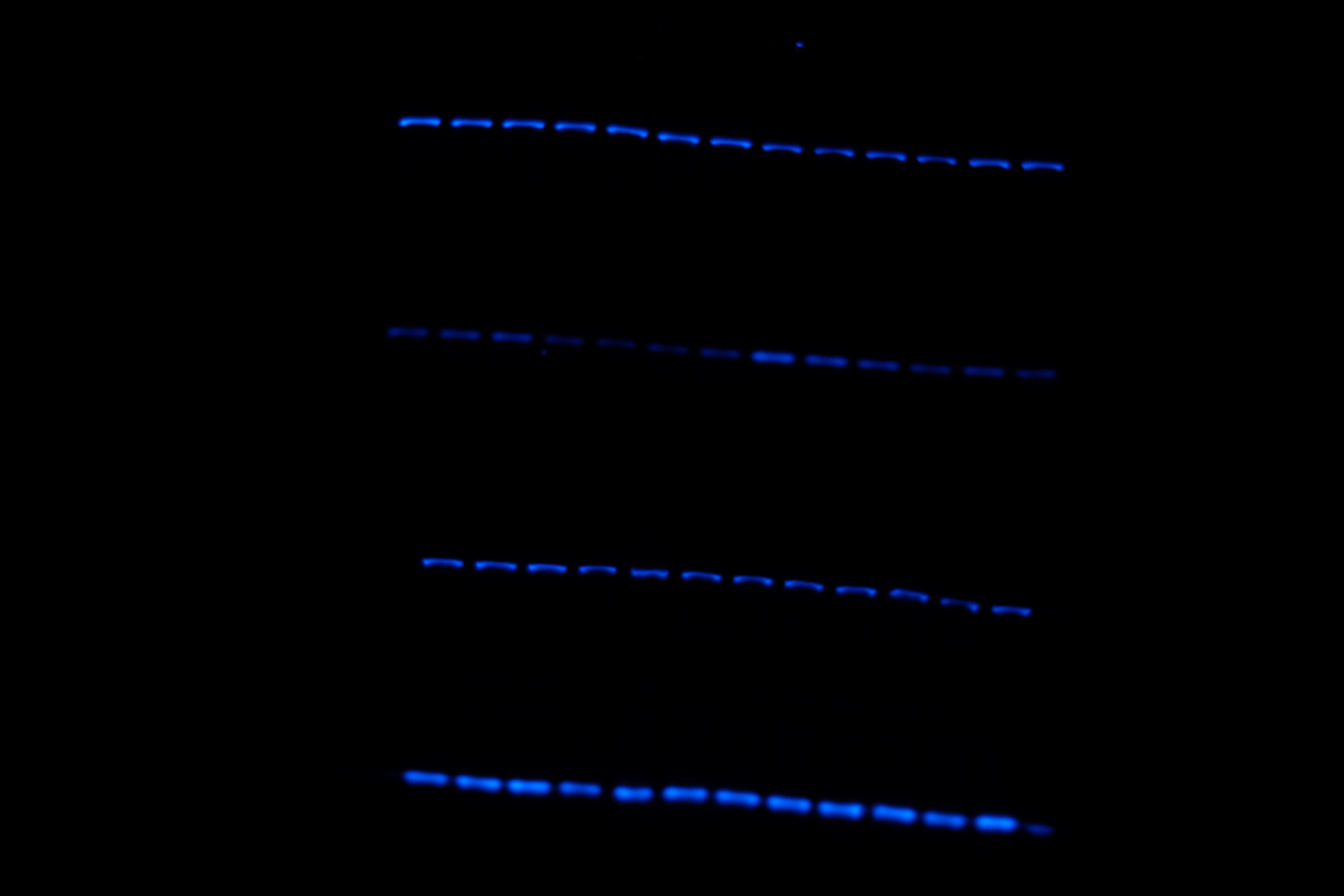

Supplement: Figure 2—figure supplement 1—source data 1. [file elife-78163-fig2-figsupp1-data1.zip › Figure 2-figure supplement 1-source data 1/Fig2-S1C_MS67_WDR5.JPG]

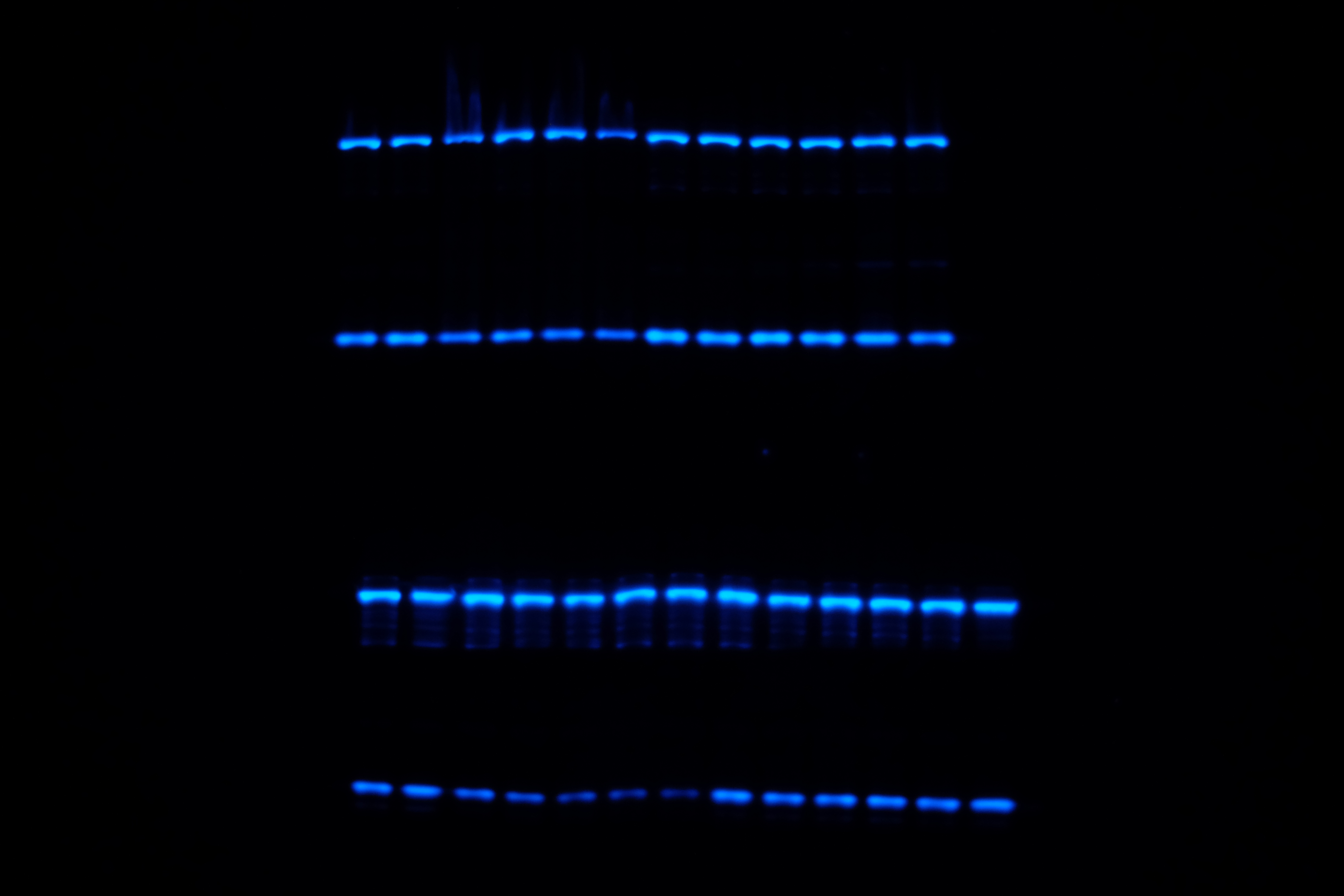

Supplement: Figure 2—figure supplement 1—source data 1. [file elife-78163-fig2-figsupp1-data1.zip › Figure 2-figure supplement 1-source data 1/Fig2-S1C_OICR-9429_WDR5_vinculin.JPG]

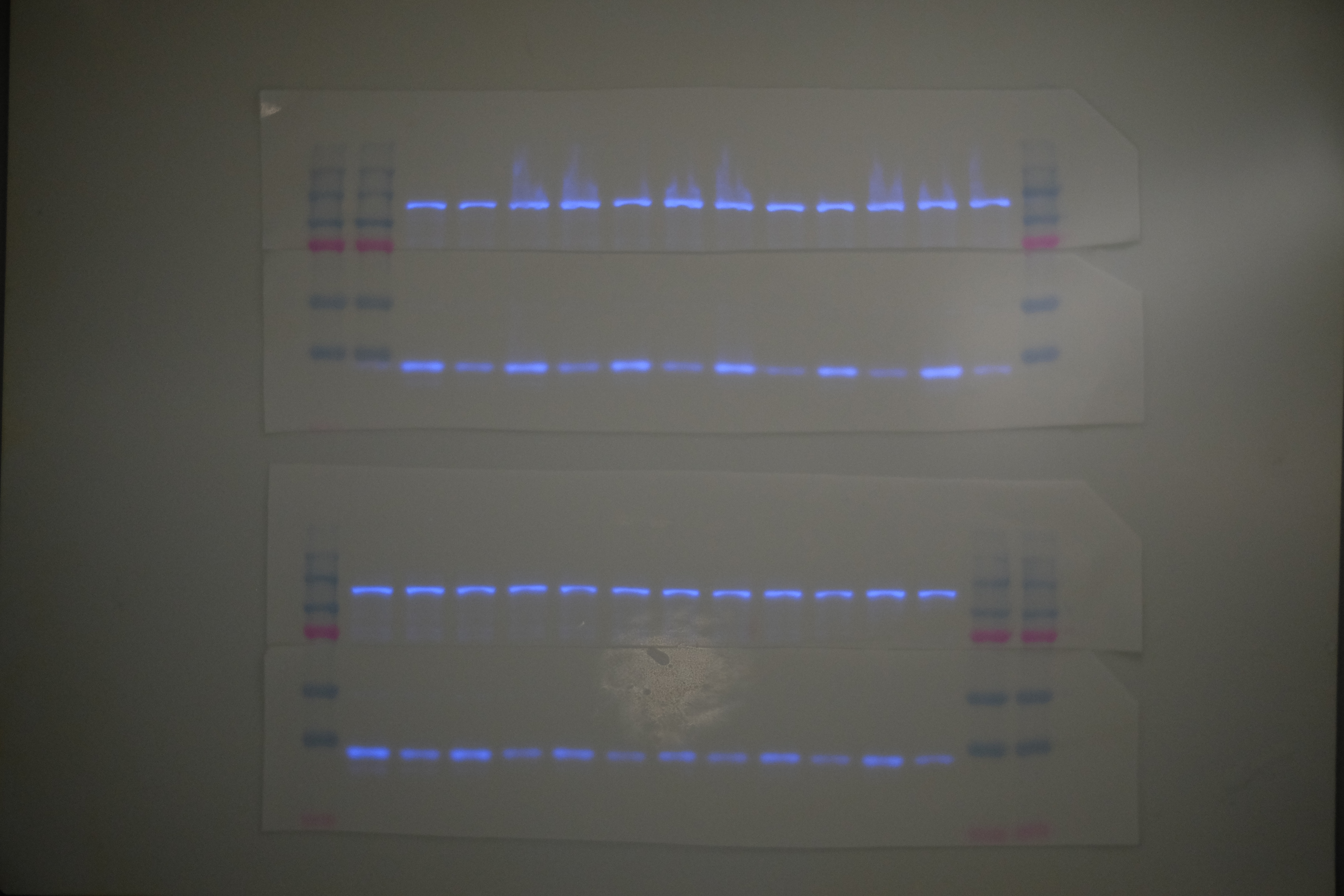

Supplement: Figure 2—figure supplement 1—source data 2. [file elife-78163-fig2-figsupp1-data2.zip › Figure 2-figure supplement 1-source data 2/DSCF4229.JPG]

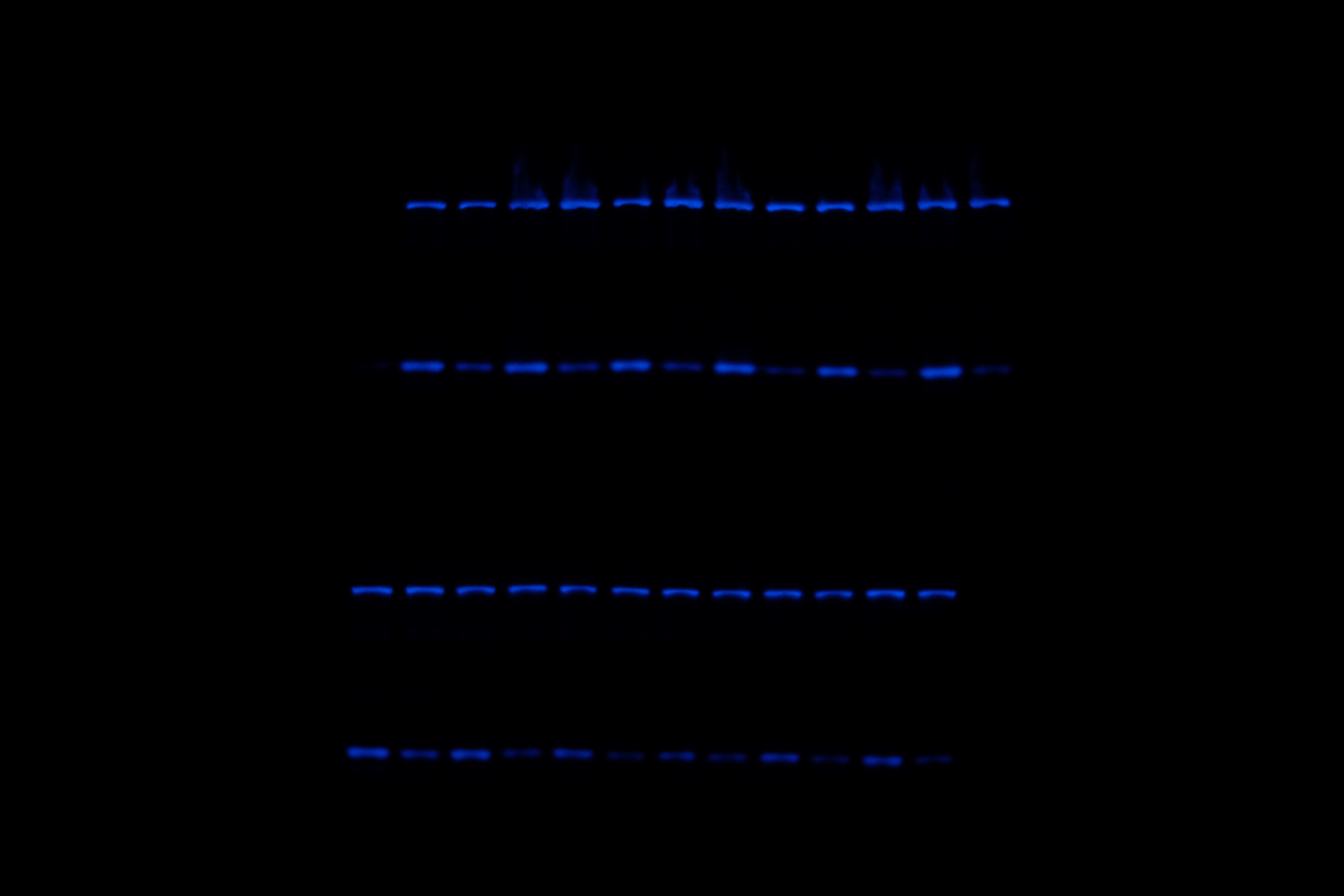

Supplement: Figure 2—figure supplement 1—source data 2. [file elife-78163-fig2-figsupp1-data2.zip › Figure 2-figure supplement 1-source data 2/Fig.2-S1D.JPG]

## Slide 1
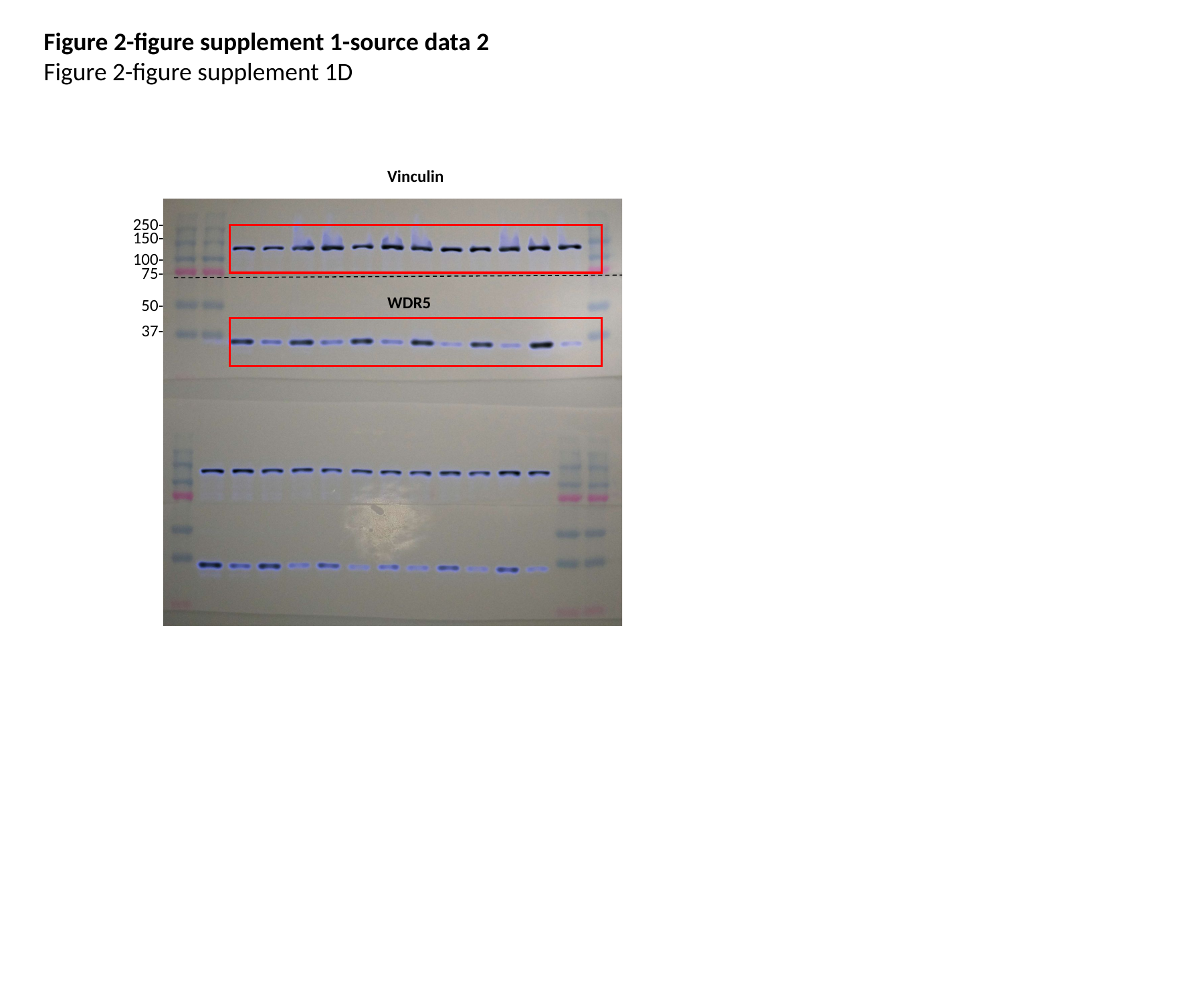

Figure 2-figure supplement 1-source data 2
Figure 2-figure supplement 1D
Vinculin
250-
150-
100-
75-
WDR5
50-
37-

Supplement: Figure 2—figure supplement 1—source data 2. [file elife-78163-fig2-figsupp1-data2.zip › Figure 2-figure supplement 1-source data 2/Figure 2-figure supplement 1-source data 2_labeled images.pptx]

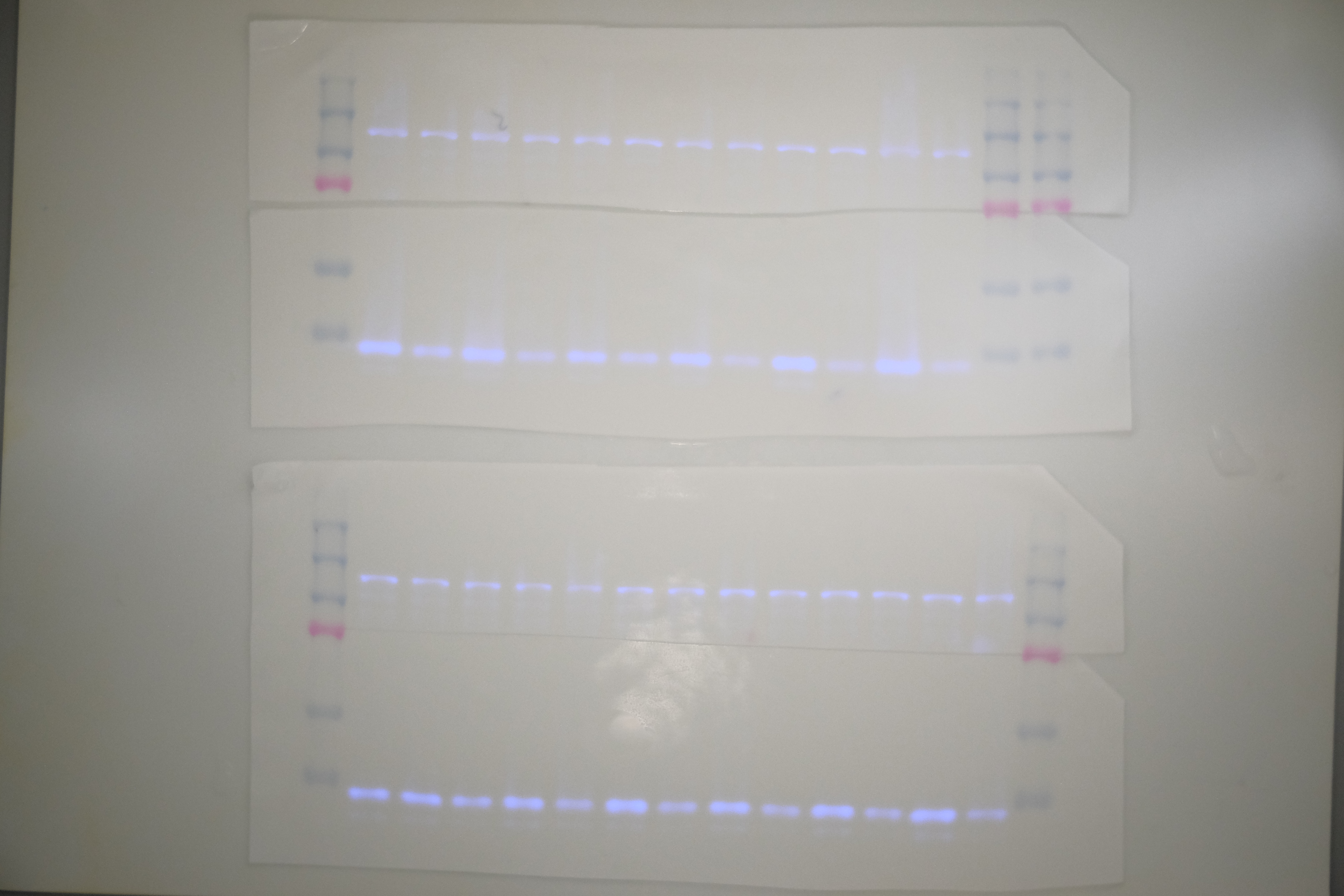

Supplement: Figure 2—figure supplement 1—source data 3. [file elife-78163-fig2-figsupp1-data3.zip › Figure 2-figure supplement 1-source data 3/DSCF4220.JPG]

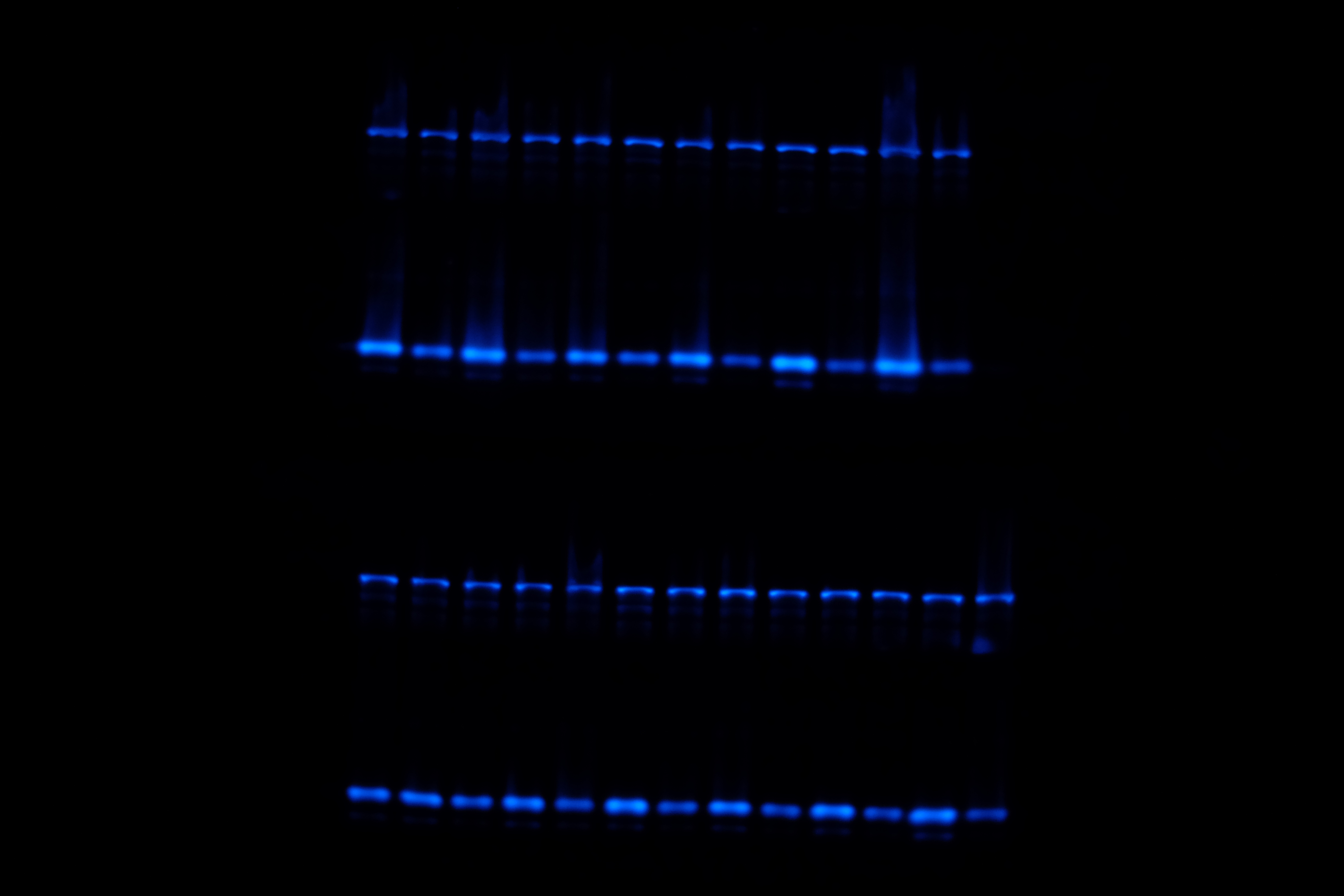

Supplement: Figure 2—figure supplement 1—source data 3. [file elife-78163-fig2-figsupp1-data3.zip › Figure 2-figure supplement 1-source data 3/Fig2-S1E_vinculin.JPG]

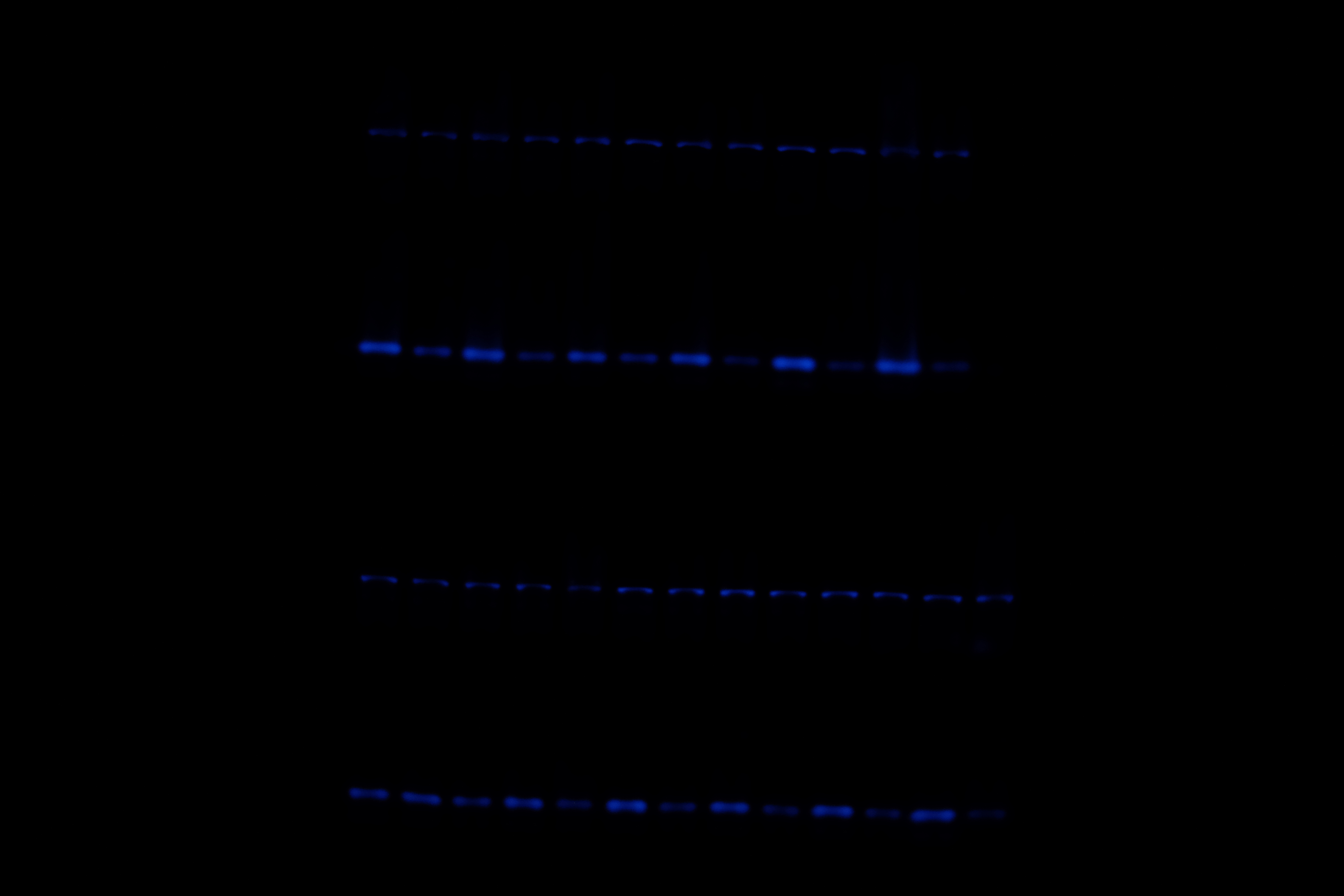

Supplement: Figure 2—figure supplement 1—source data 3. [file elife-78163-fig2-figsupp1-data3.zip › Figure 2-figure supplement 1-source data 3/Fig2-S1E_WDR5.JPG]

## Slide 1
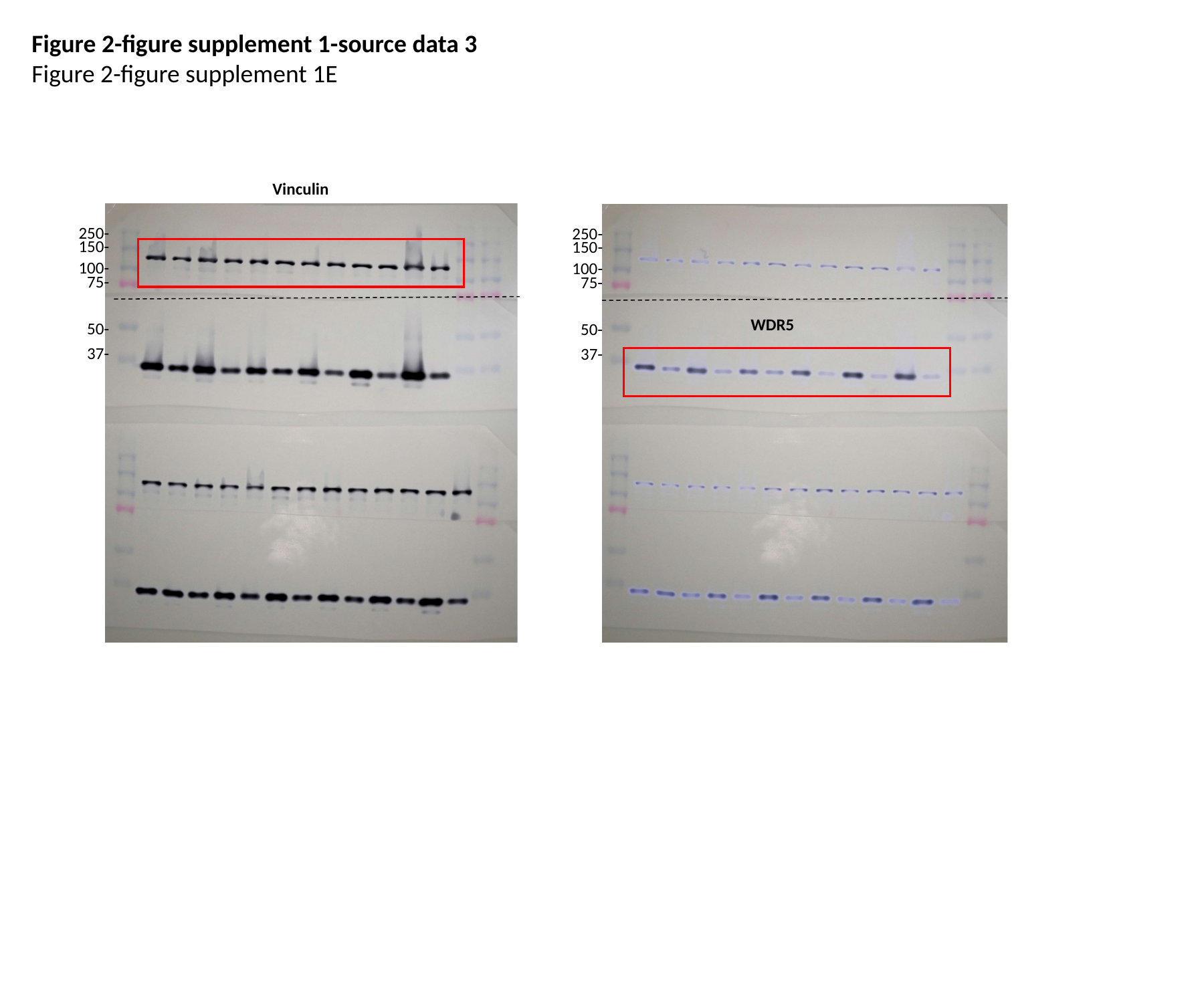

Figure 2-figure supplement 1-source data 3
Figure 2-figure supplement 1E
Vinculin
250-
250-
150-
150-
100-
100-
75-
75-
WDR5
50-
50-
37-
37-

Supplement: Figure 2—figure supplement 1—source data 3. [file elife-78163-fig2-figsupp1-data3.zip › Figure 2-figure supplement 1-source data 3/Figure 2-figure supplement 1-source data 3_labeled images.pptx]

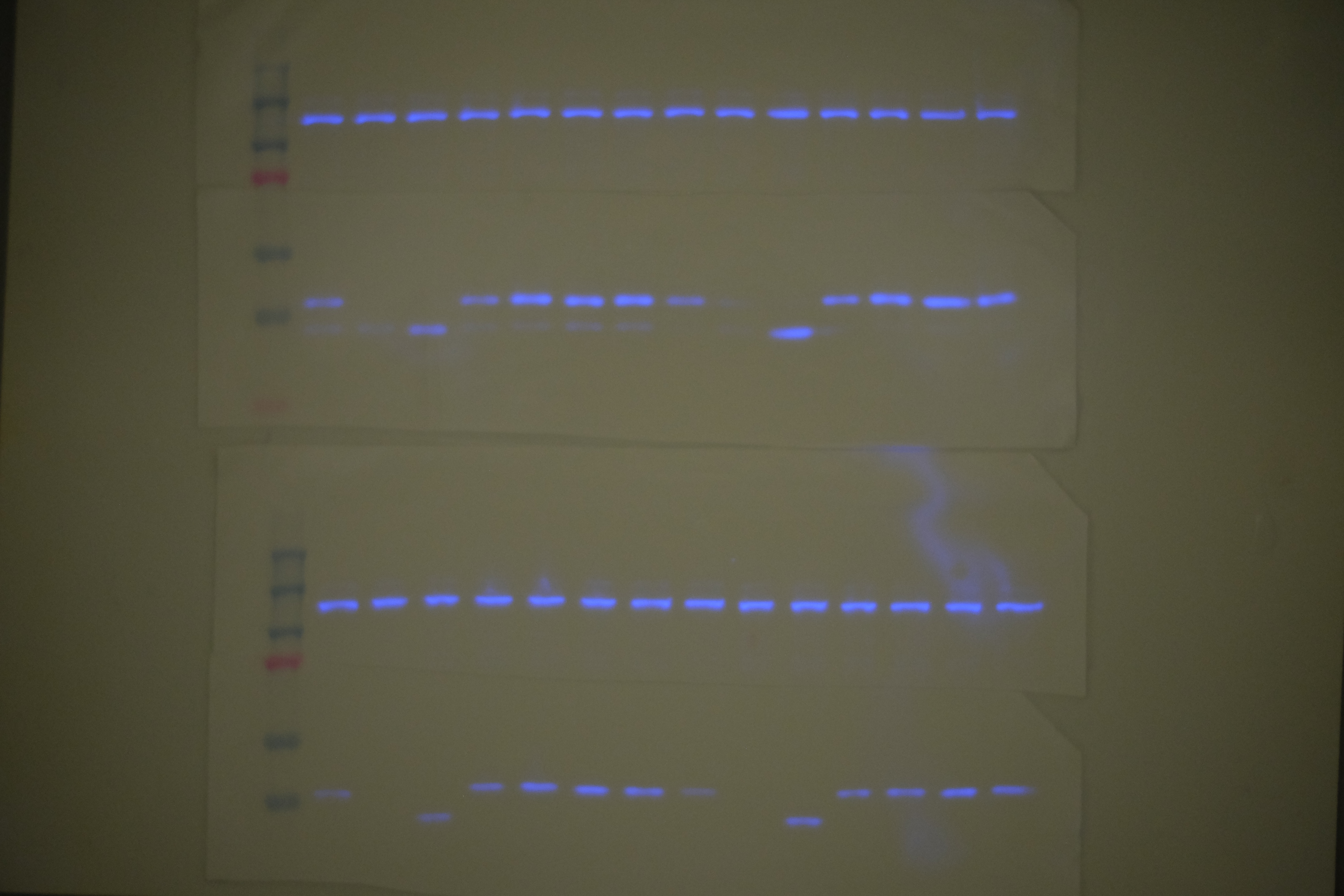

Supplement: Figure 4—source data 1. [file elife-78163-fig4-data1.zip › Figure 4-source data 1/DSCF0587.JPG]

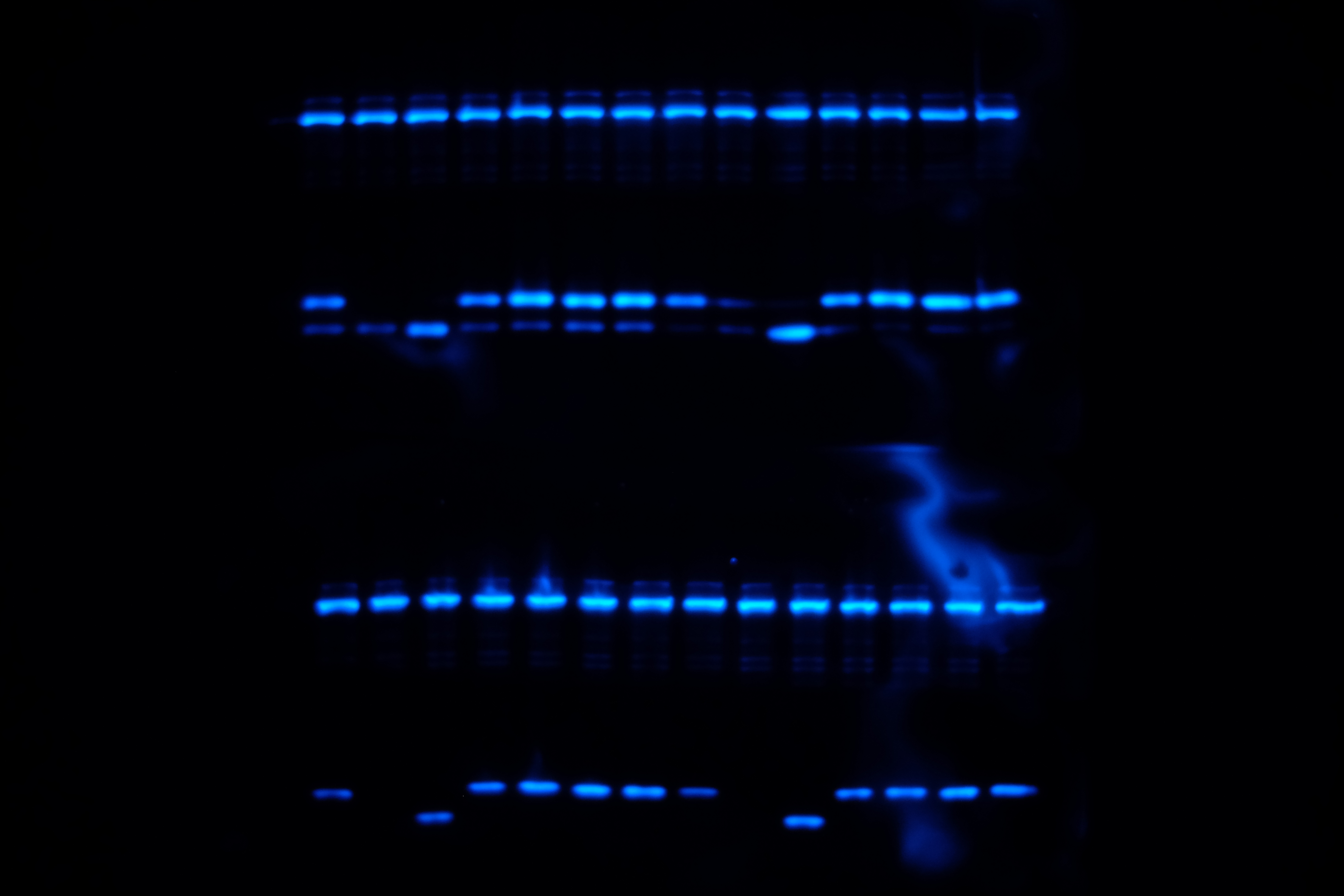

Supplement: Figure 4—source data 1. [file elife-78163-fig4-data1.zip › Figure 4-source data 1/Fig.4C_Flag.JPG]

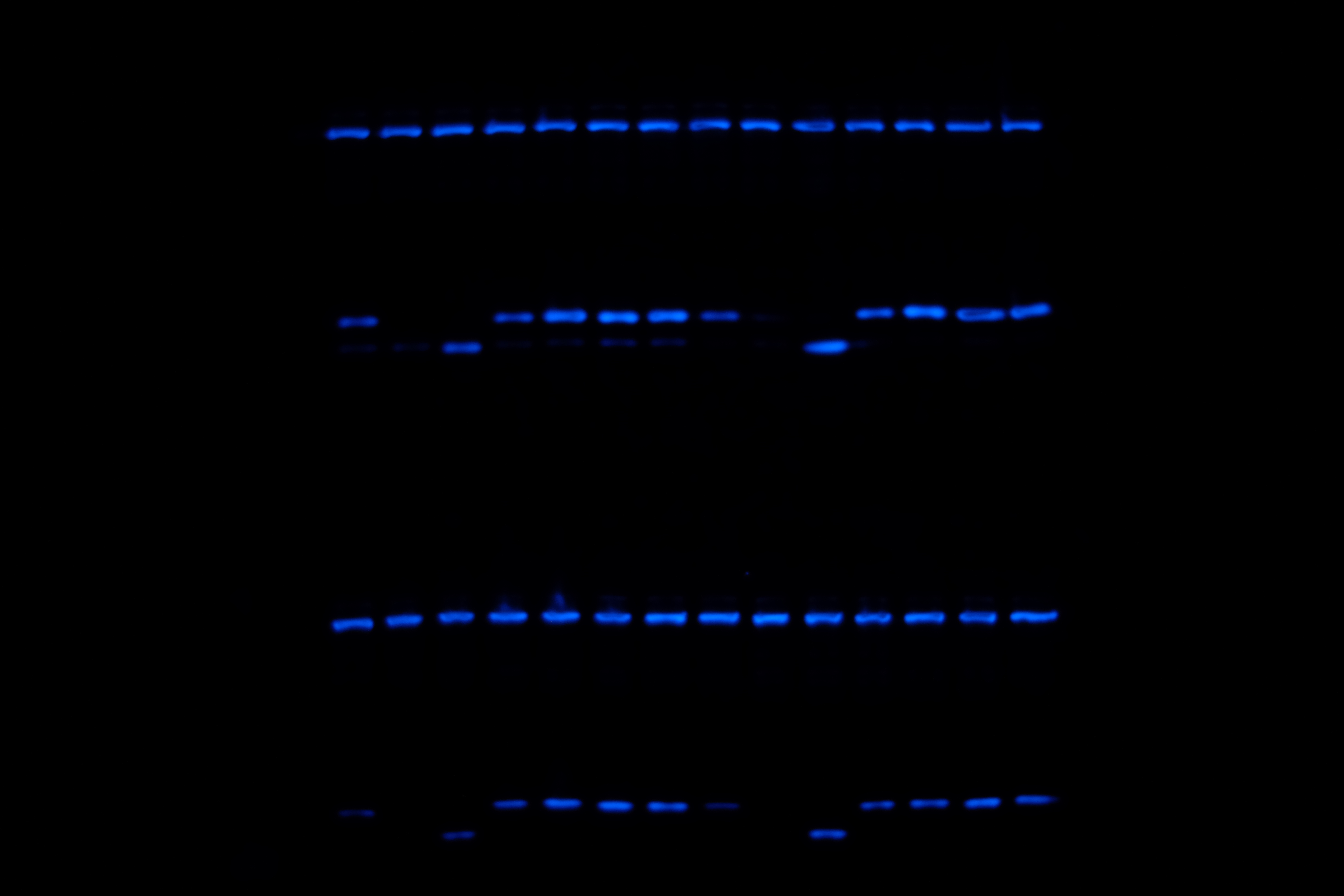

Supplement: Figure 4—source data 1. [file elife-78163-fig4-data1.zip › Figure 4-source data 1/Fig.4C_Vinculin.JPG]

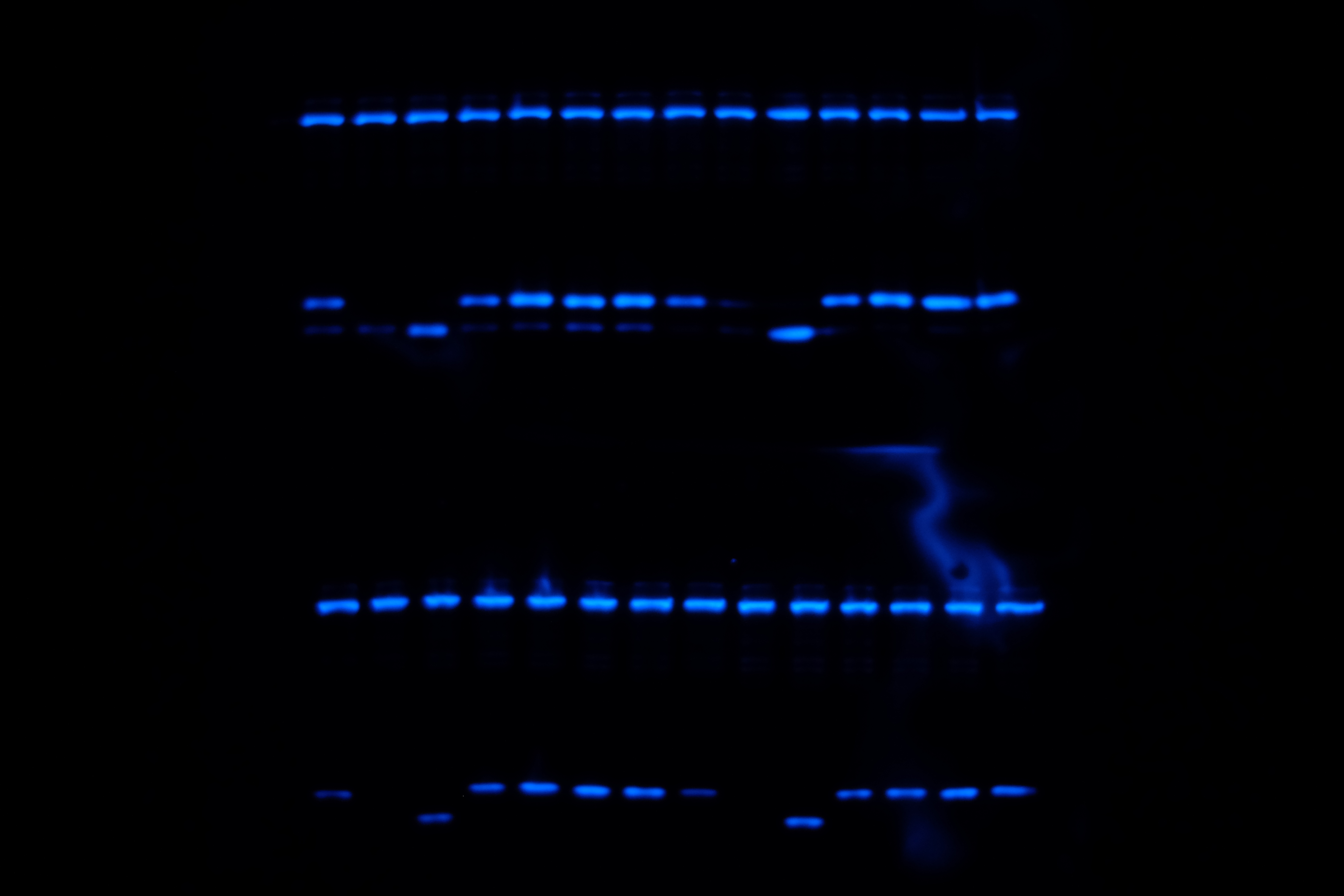

Supplement: Figure 4—source data 1. [file elife-78163-fig4-data1.zip › Figure 4-source data 1/Fig.4C_WDR5.JPG]

## Slide 1
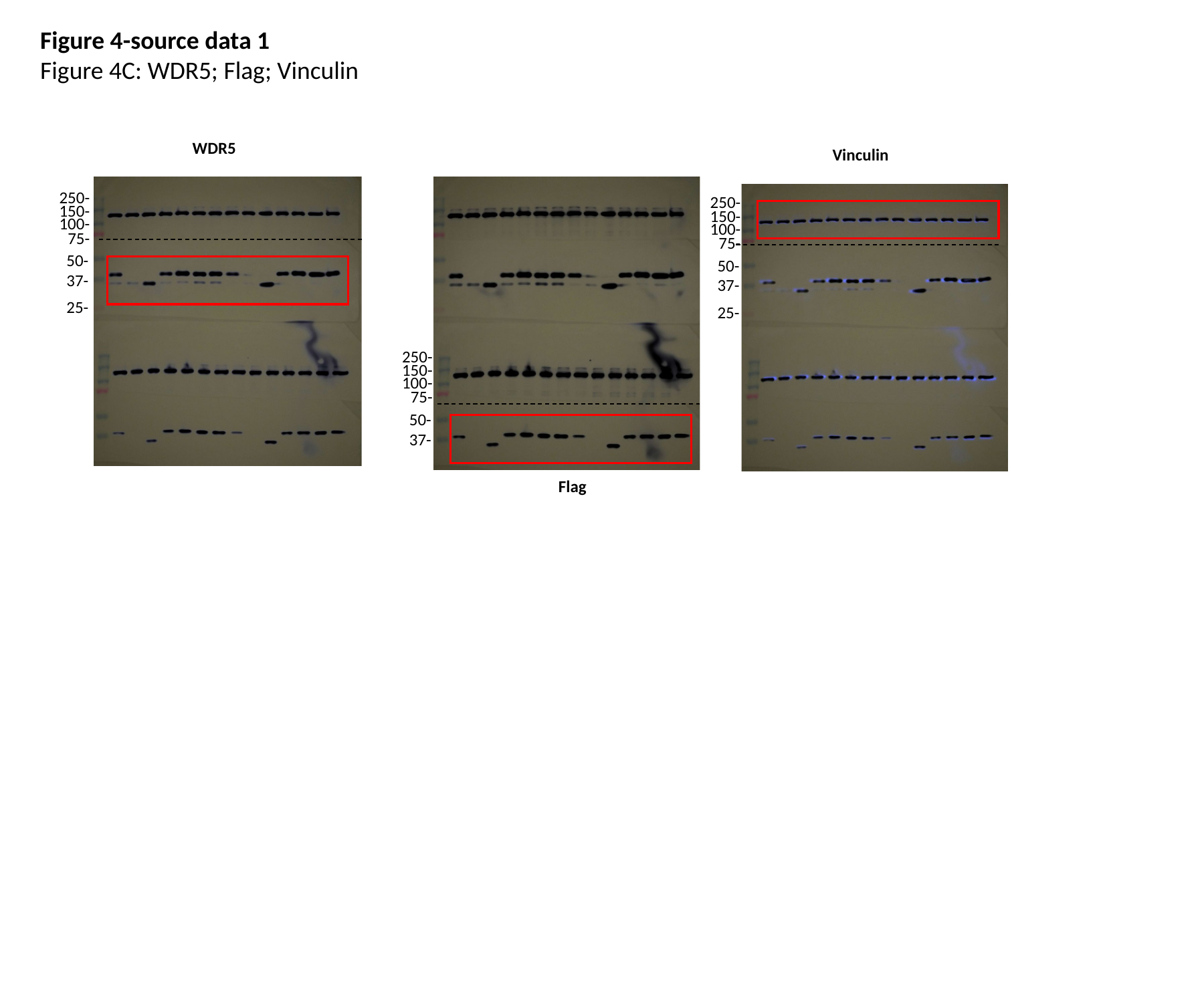

Figure 4-source data 1
Figure 4C: WDR5; Flag; Vinculin
WDR5
Vinculin
250-
250-
150-
150-
100-
100-
75-
75-
50-
50-
37-
37-
25-
25-
250-
150-
100-
75-
50-
37-
Flag

Supplement: Figure 4—source data 1. [file elife-78163-fig4-data1.zip › Figure 4-source data 1/Figure 4-source data 1_labeled images.pptx]

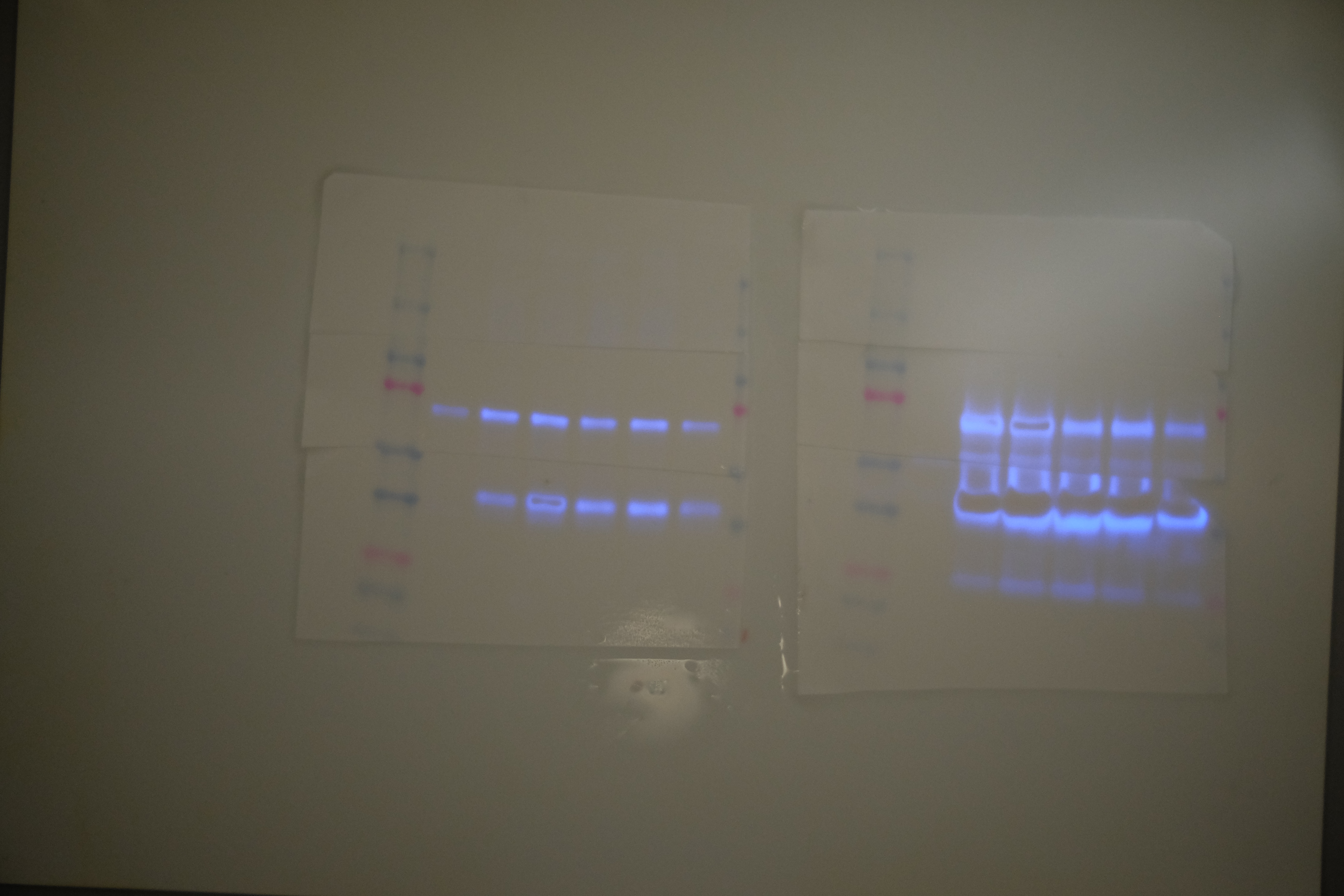

Supplement: Figure 4—source data 2. [file elife-78163-fig4-data2.zip › Figure 4-source data 2/DSCF3917.JPG]

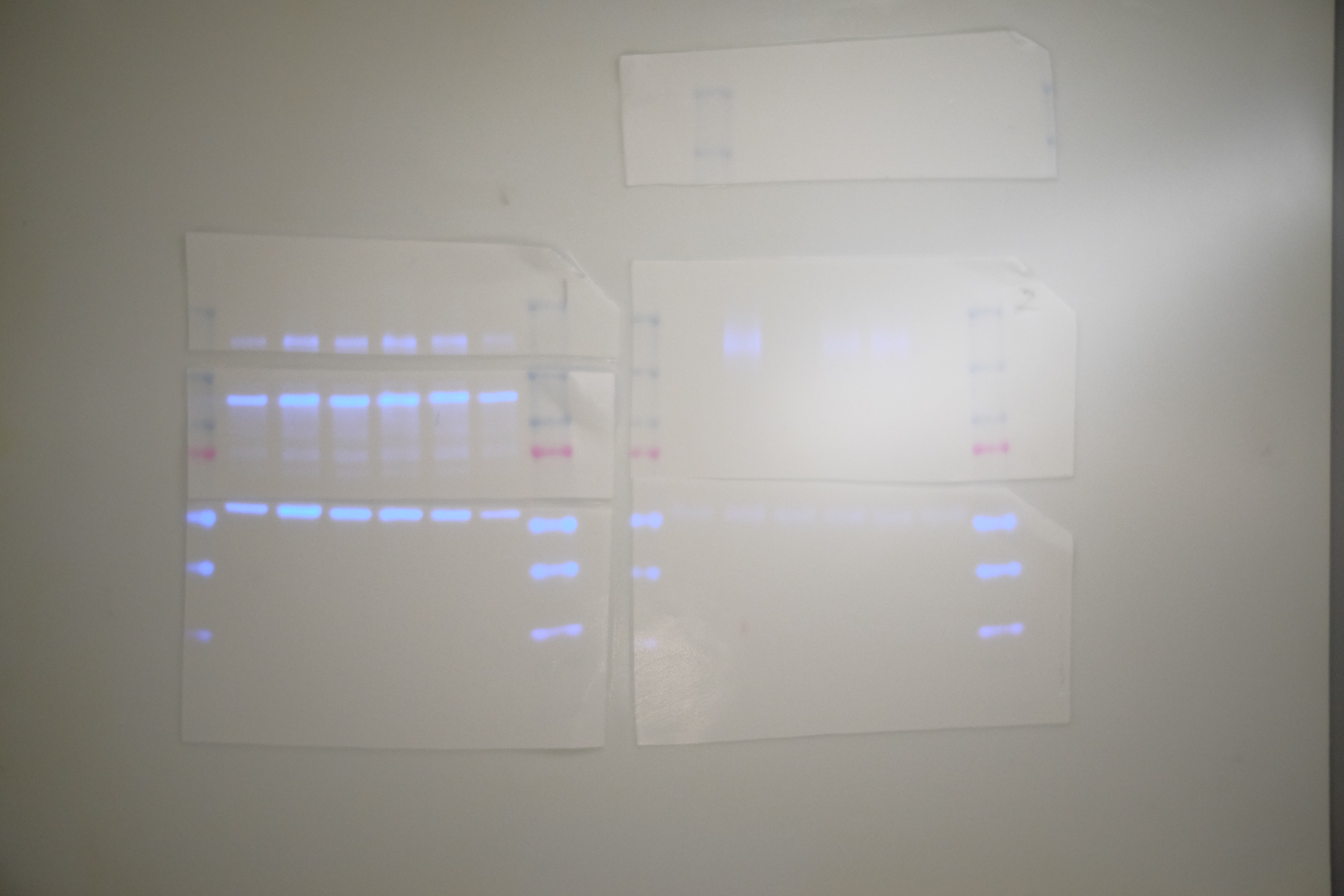

Supplement: Figure 4—source data 2. [file elife-78163-fig4-data2.zip › Figure 4-source data 2/DSCF3924.JPG]

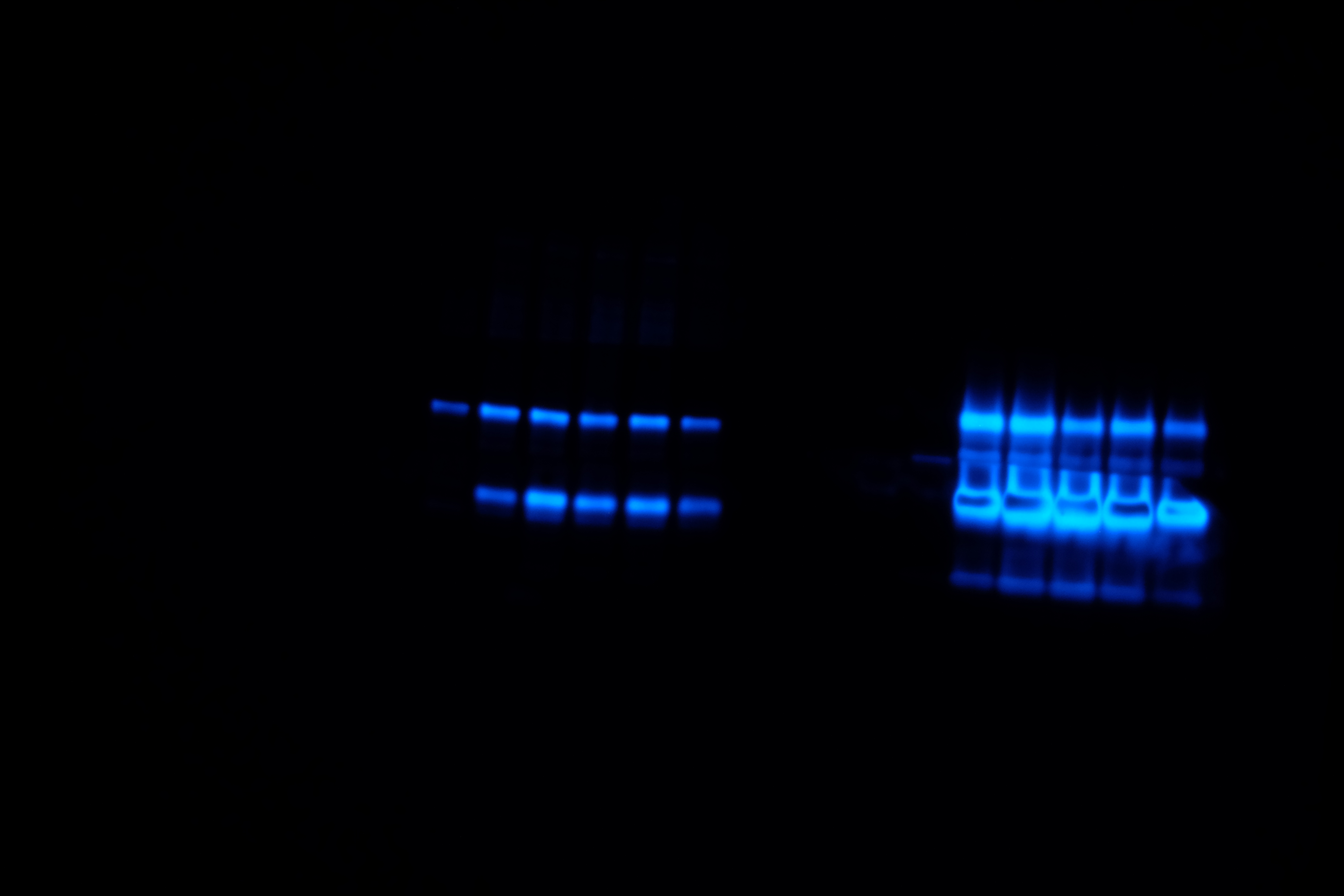

Supplement: Figure 4—source data 2. [file elife-78163-fig4-data2.zip › Figure 4-source data 2/Fig.4D_input_WDR5_RBBP5.JPG]

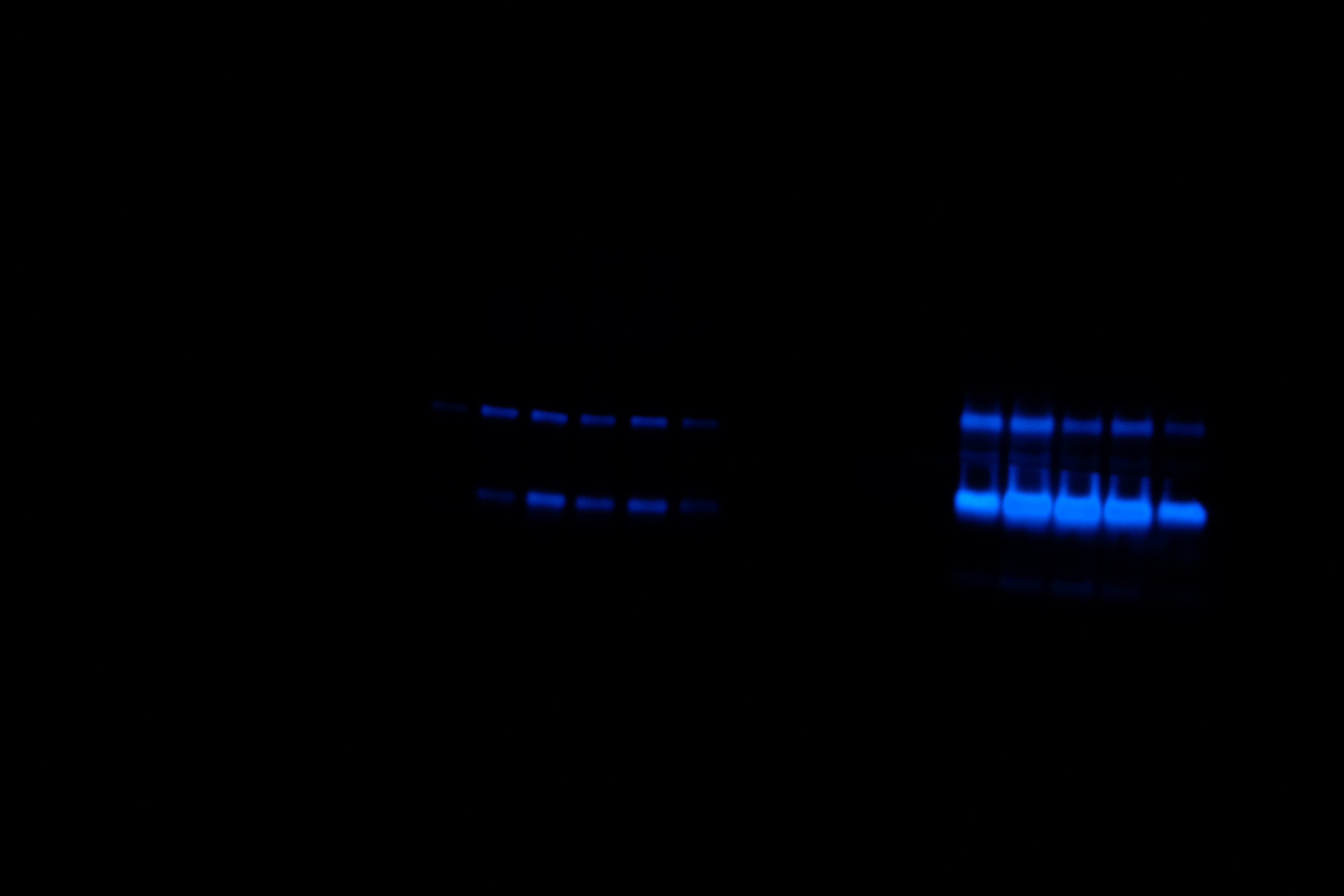

Supplement: Figure 4—source data 2. [file elife-78163-fig4-data2.zip › Figure 4-source data 2/Fig.4D_IP_WDR5_RBBP5.JPG]

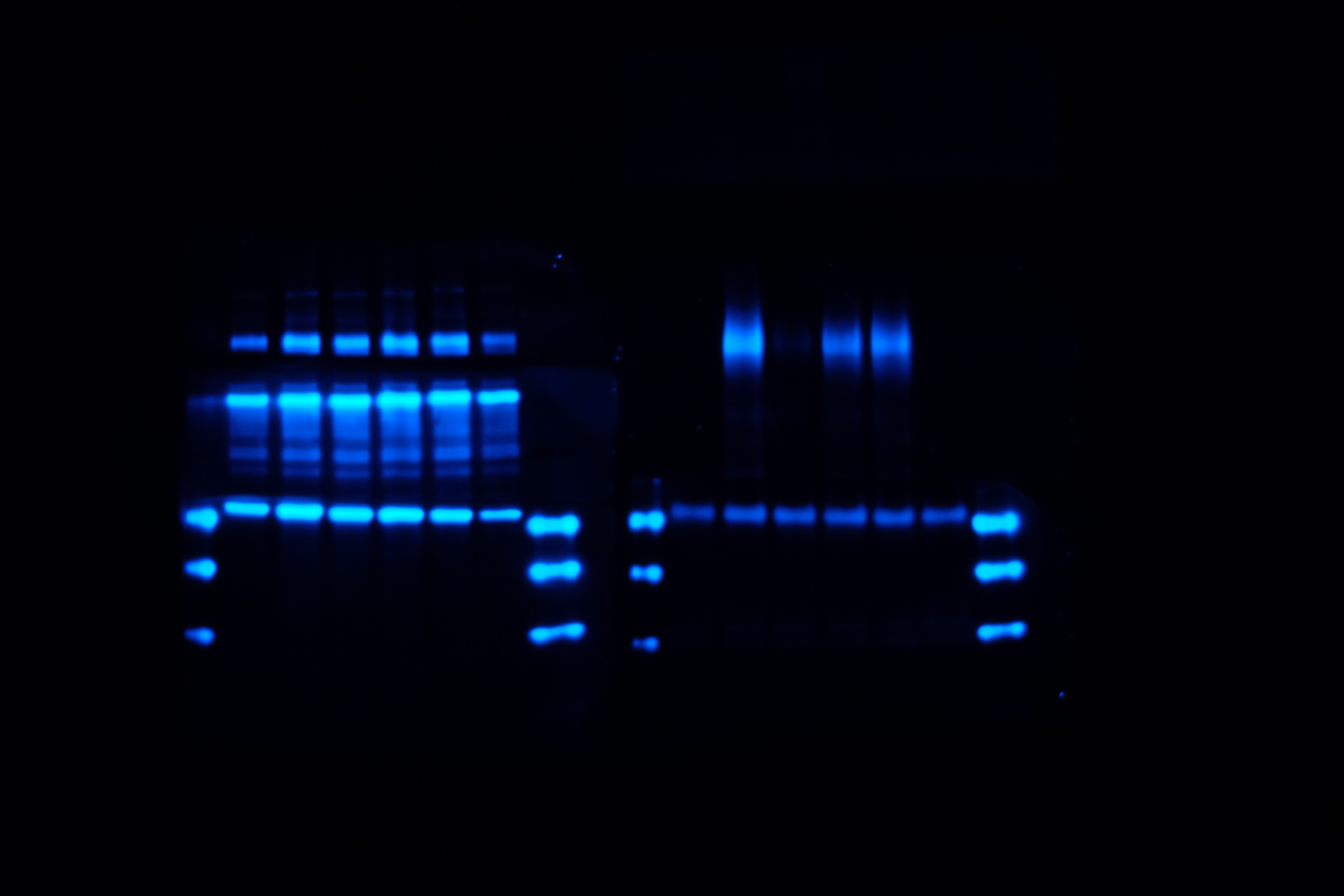

Supplement: Figure 4—source data 2. [file elife-78163-fig4-data2.zip › Figure 4-source data 2/Fig.4D_KMT2A.JPG]

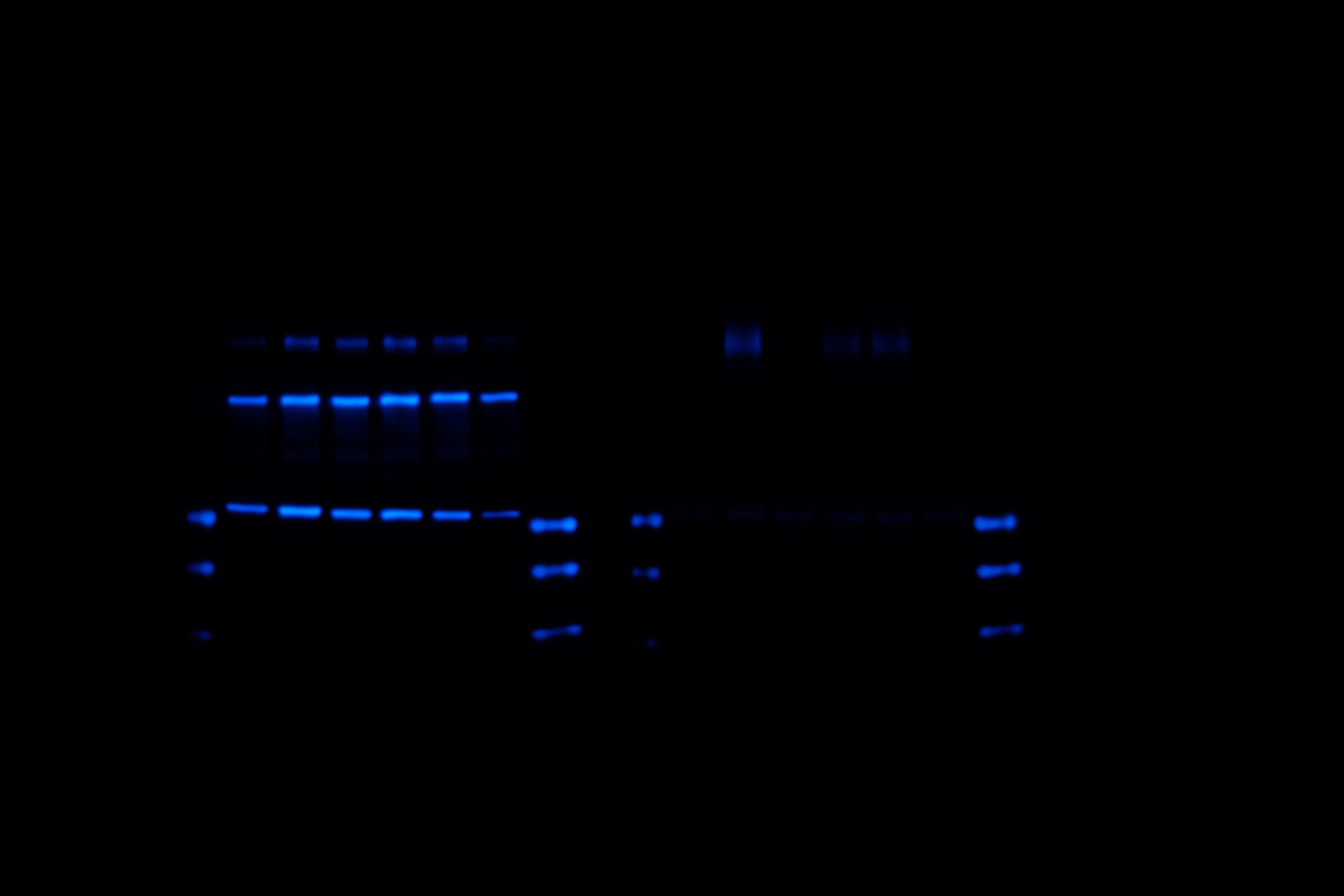

Supplement: Figure 4—source data 2. [file elife-78163-fig4-data2.zip › Figure 4-source data 2/Fig.4D_vinculin.JPG]

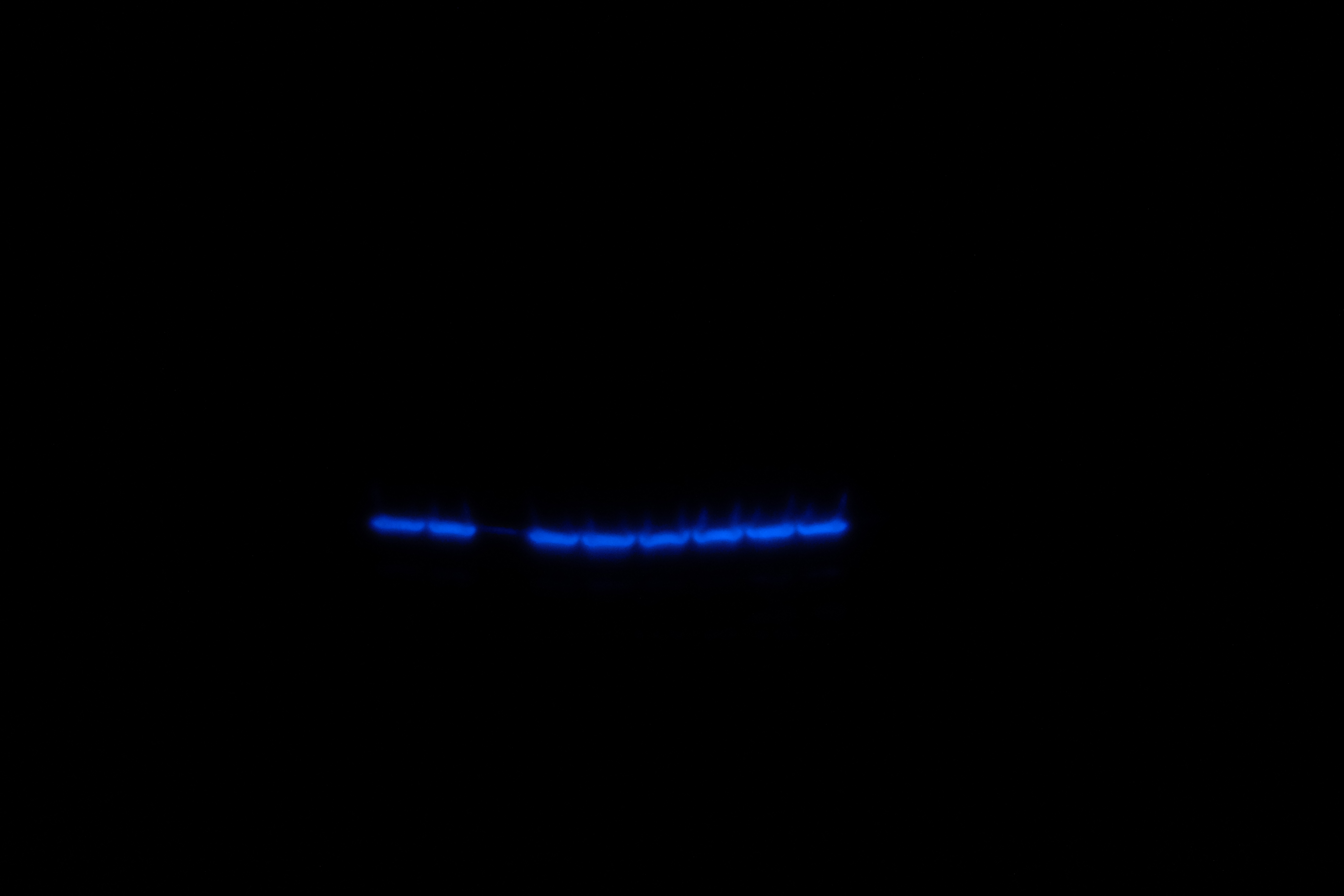

Supplement: Figure 5—source data 1. [file elife-78163-fig5-data1.zip › Figure 5-source data 1/5B_GAPDH_for_shKMT2A.JPG]

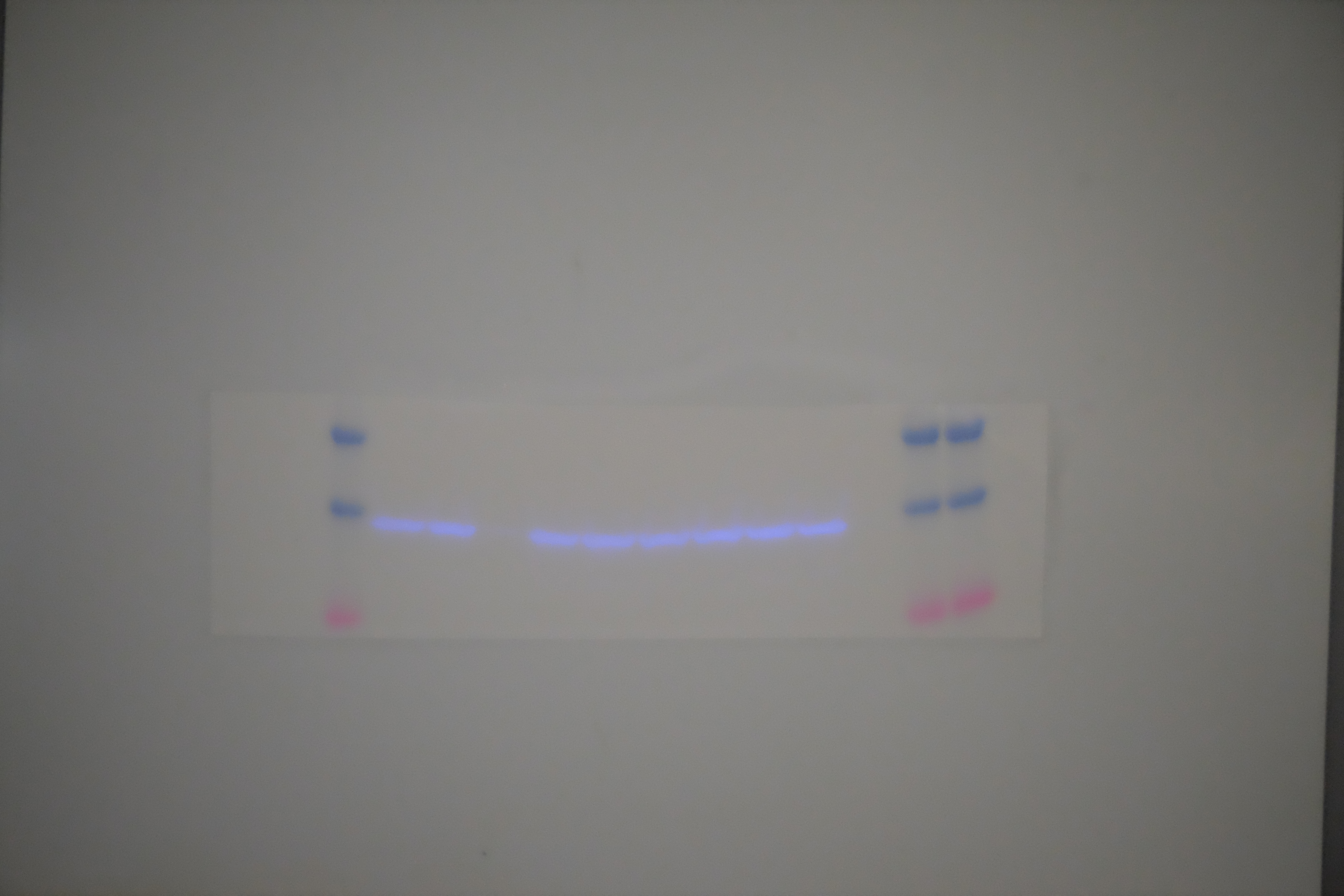

Supplement: Figure 5—source data 1. [file elife-78163-fig5-data1.zip › Figure 5-source data 1/5B_GAPDH_light_for_shKMT2A.JPG]

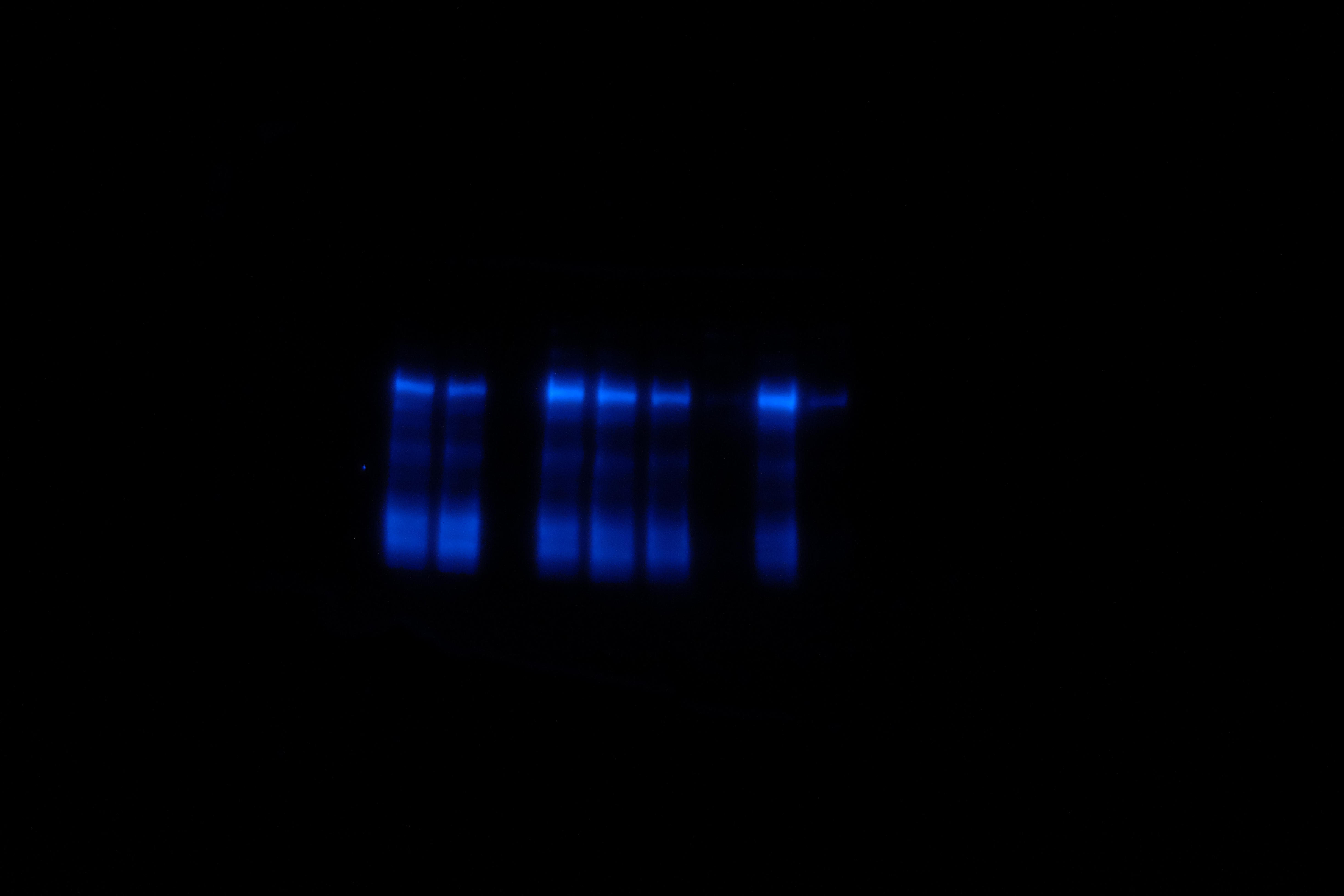

Supplement: Figure 5—source data 1. [file elife-78163-fig5-data1.zip › Figure 5-source data 1/5B_shKMT2A.JPG]

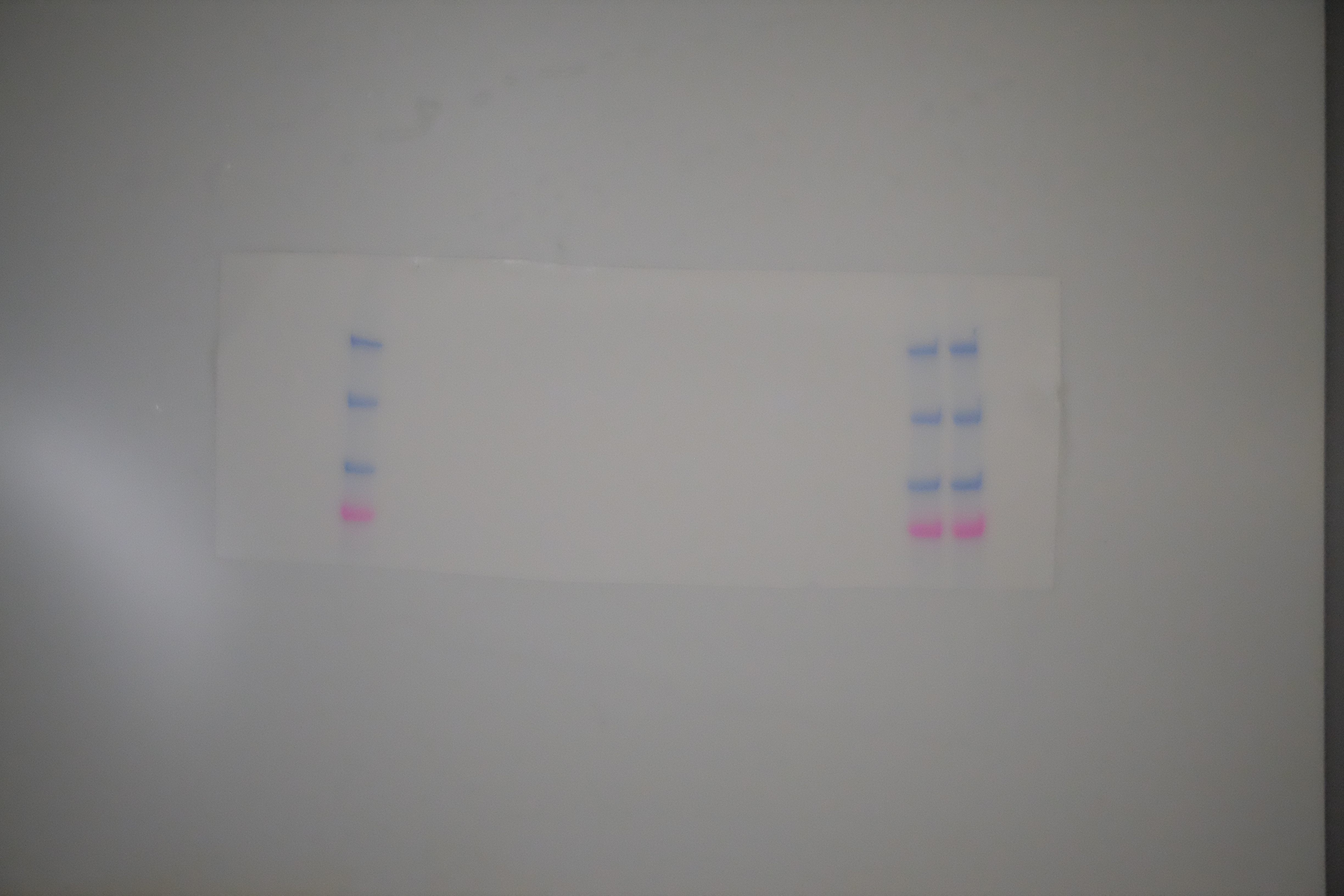

Supplement: Figure 5—source data 1. [file elife-78163-fig5-data1.zip › Figure 5-source data 1/5B_shKMT2A_light.JPG]

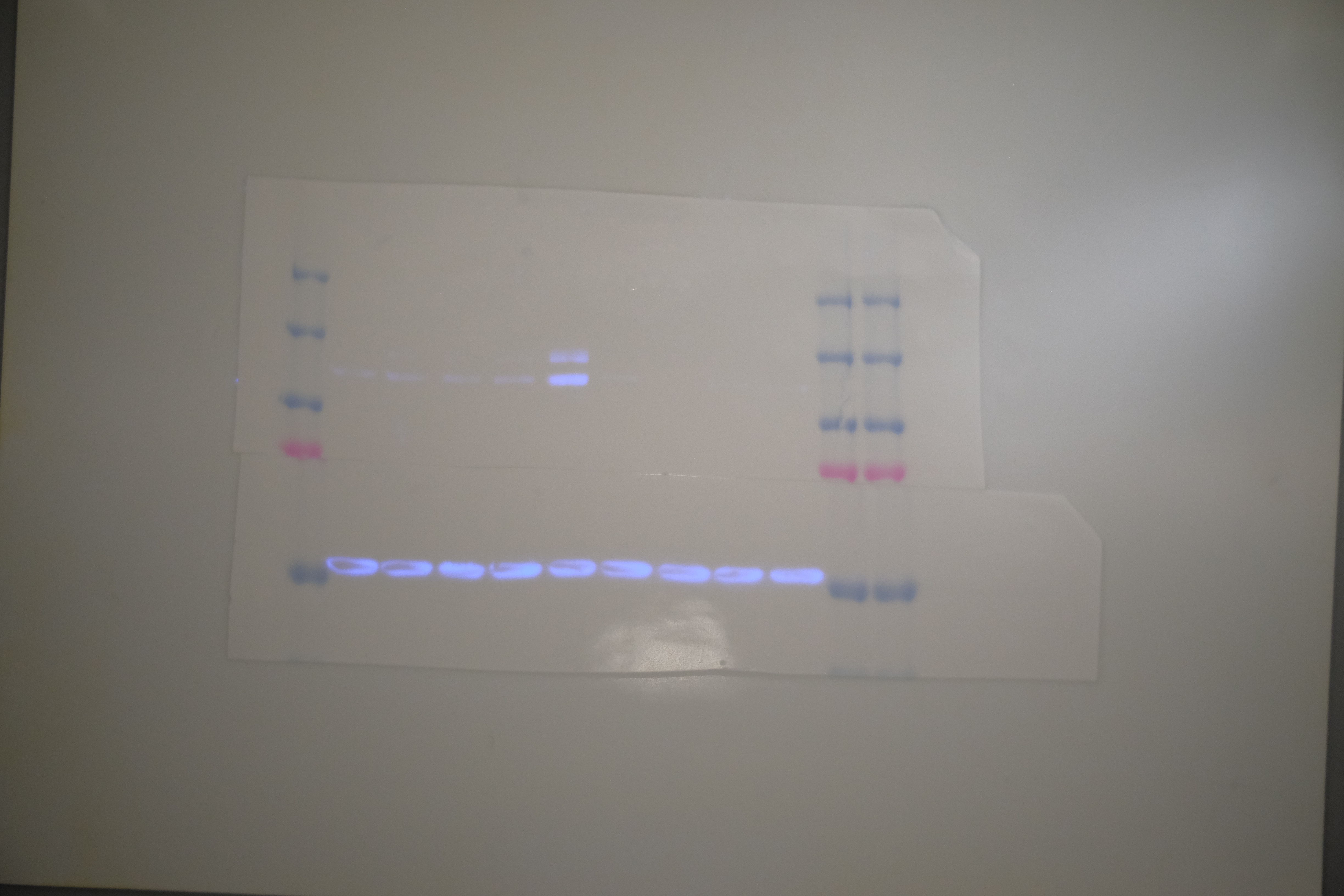

Supplement: Figure 5—source data 1. [file elife-78163-fig5-data1.zip › Figure 5-source data 1/DSCF2763.JPG]

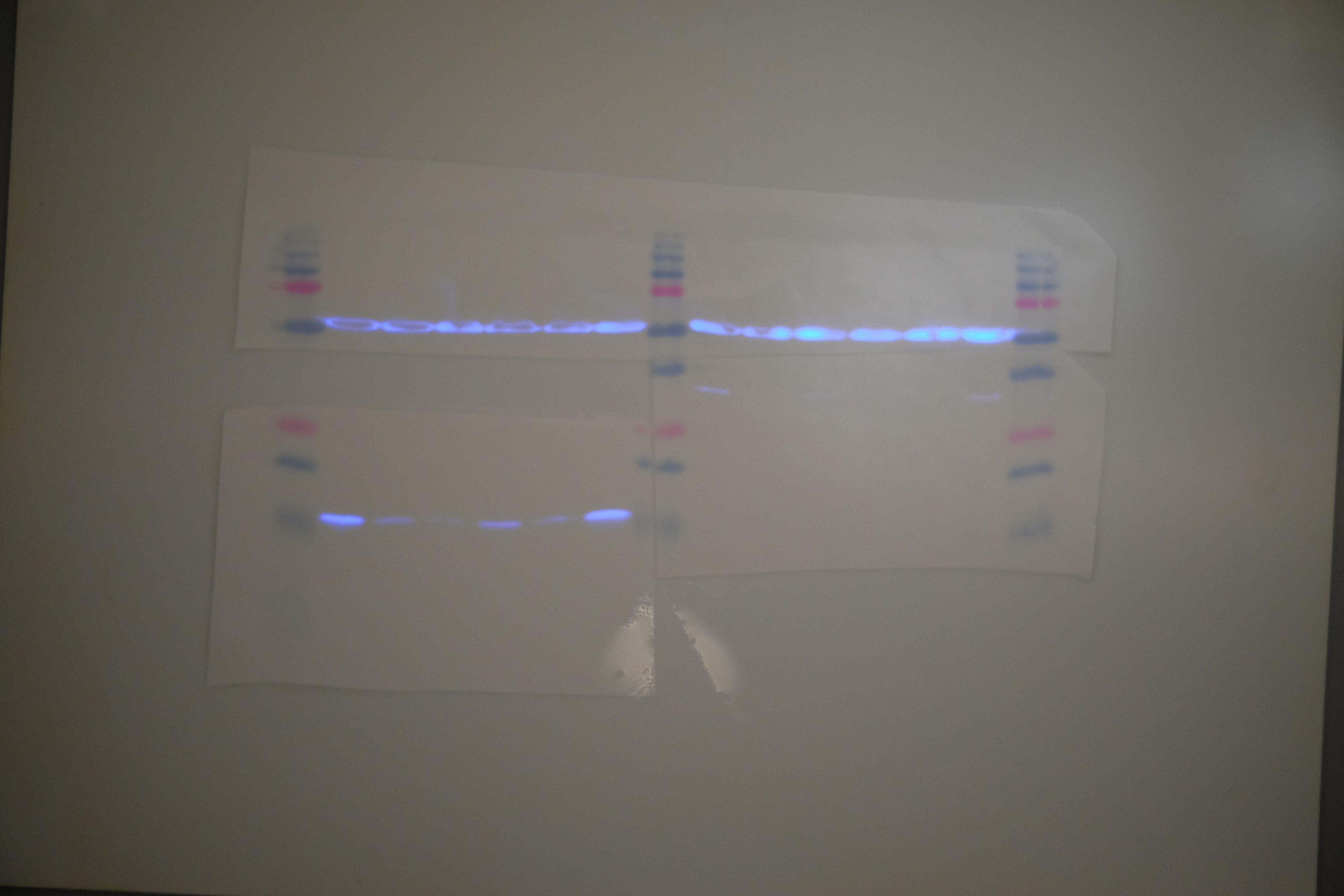

Supplement: Figure 5—source data 1. [file elife-78163-fig5-data1.zip › Figure 5-source data 1/DSCF2767.JPG]

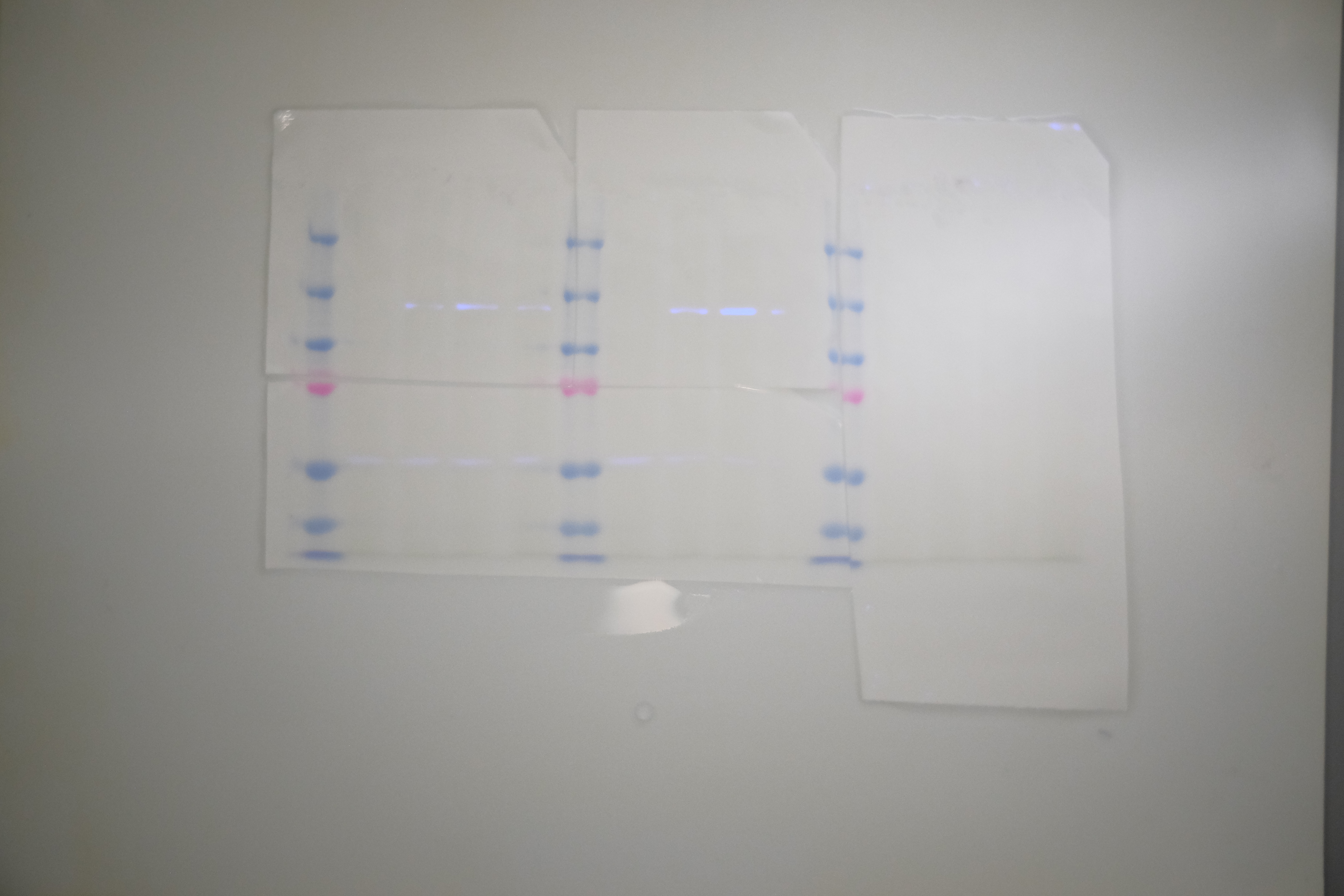

Supplement: Figure 5—source data 1. [file elife-78163-fig5-data1.zip › Figure 5-source data 1/DSCF2831.JPG]

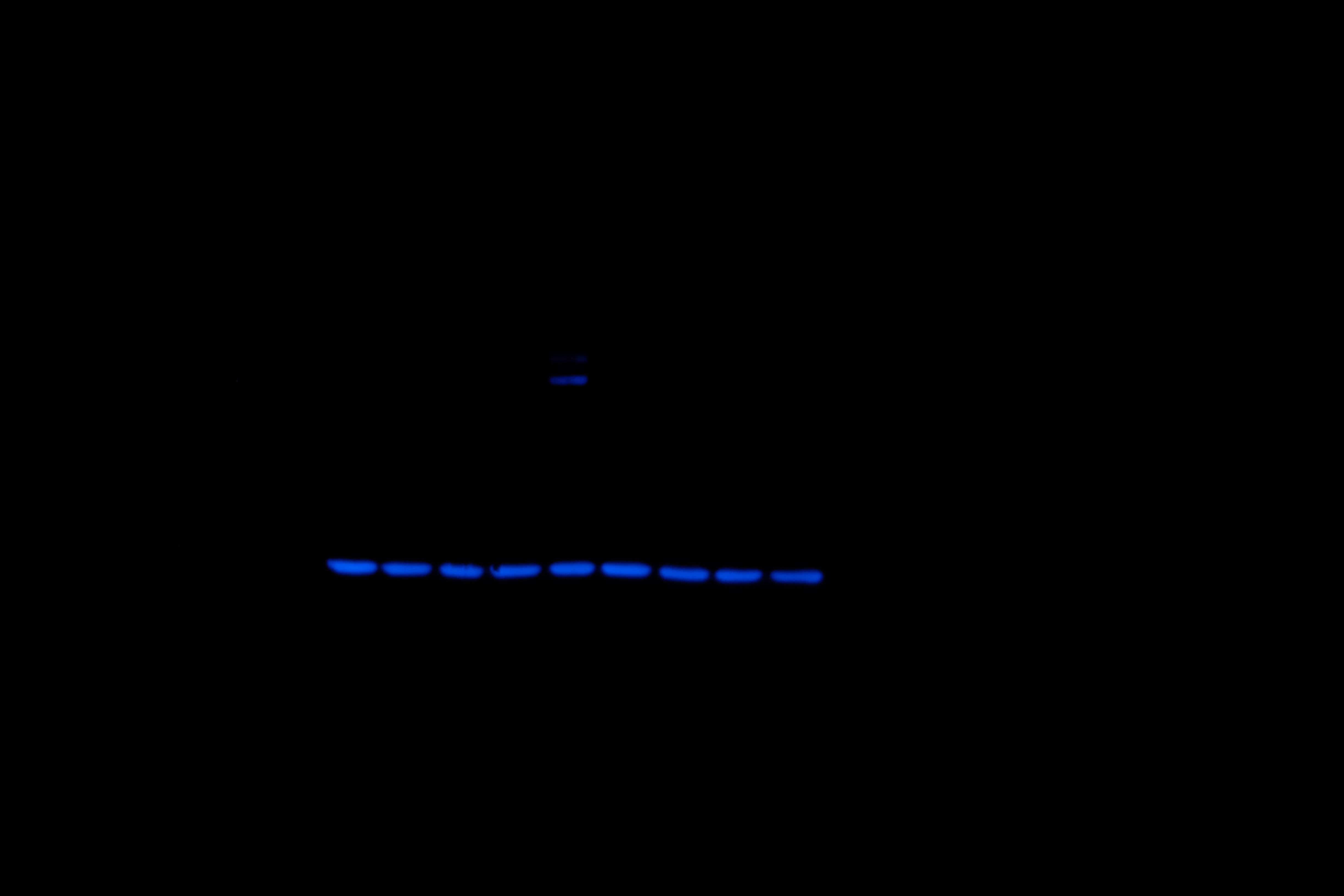

Supplement: Figure 5—source data 1. [file elife-78163-fig5-data1.zip › Figure 5-source data 1/Fig.5B_a-tubulin for HCFC1.JPG]

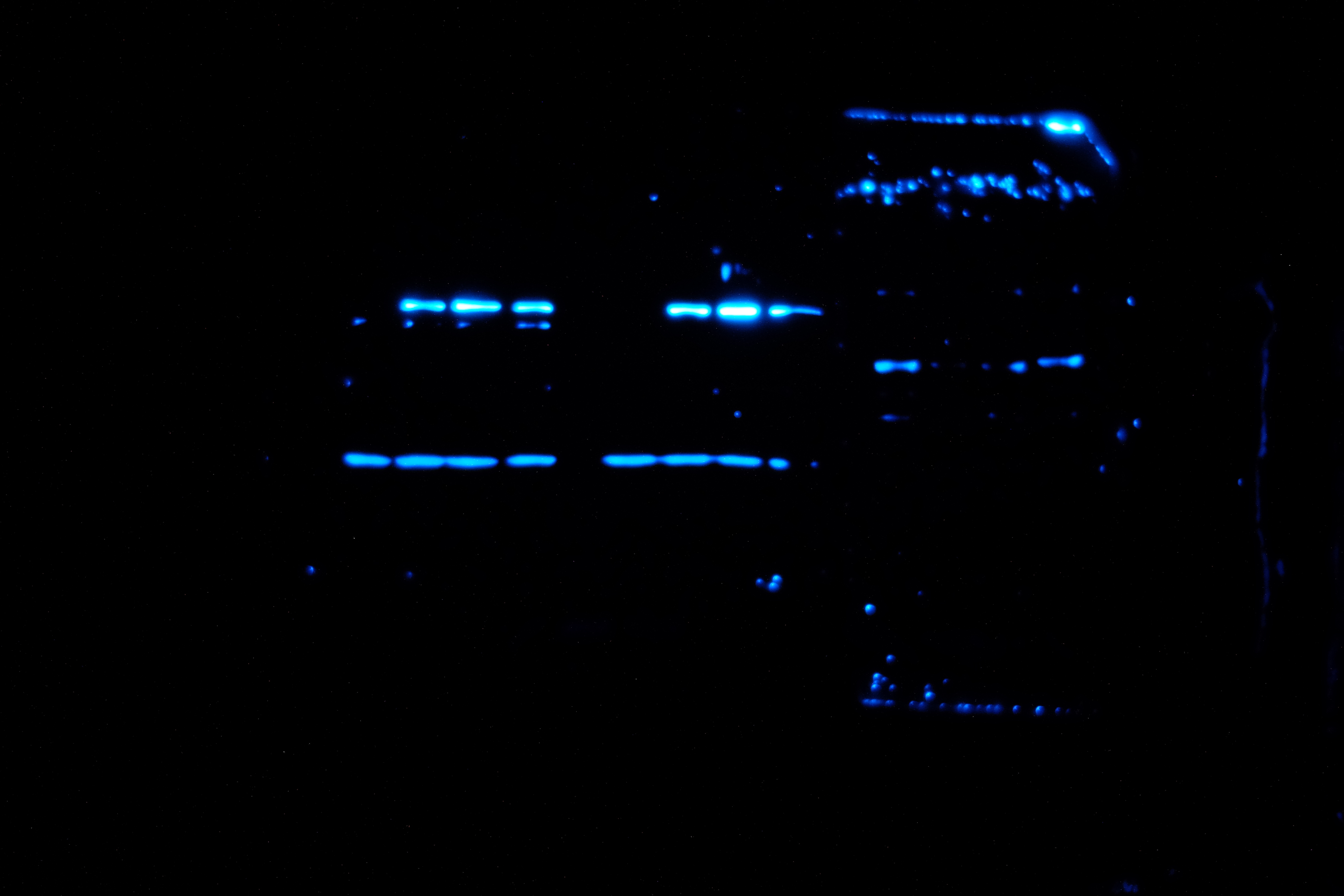

Supplement: Figure 5—source data 1. [file elife-78163-fig5-data1.zip › Figure 5-source data 1/Fig.5B_CXXC1.JPG]

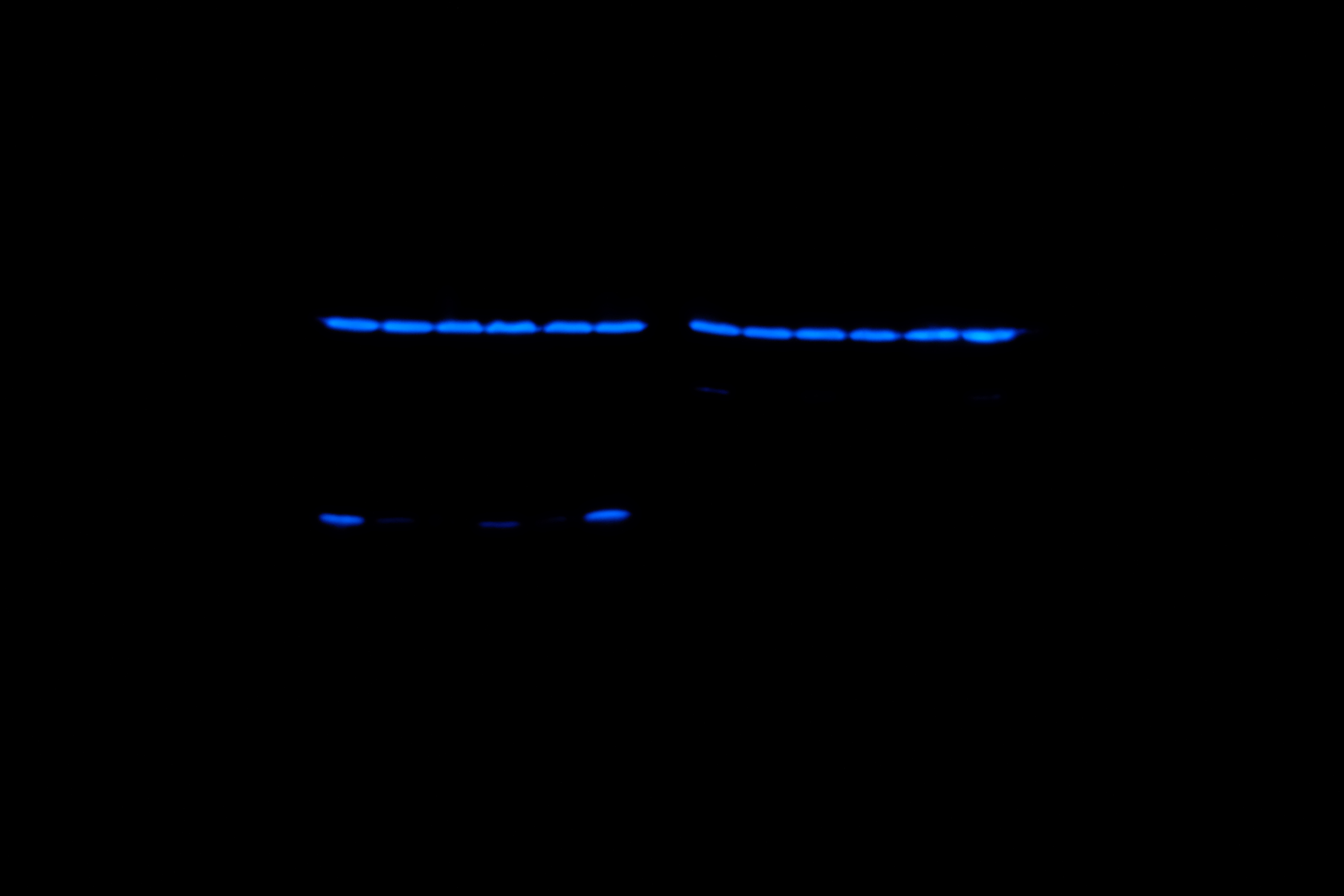

Supplement: Figure 5—source data 1. [file elife-78163-fig5-data1.zip › Figure 5-source data 1/Fig.5B_DPY30_a-tubulin.JPG]

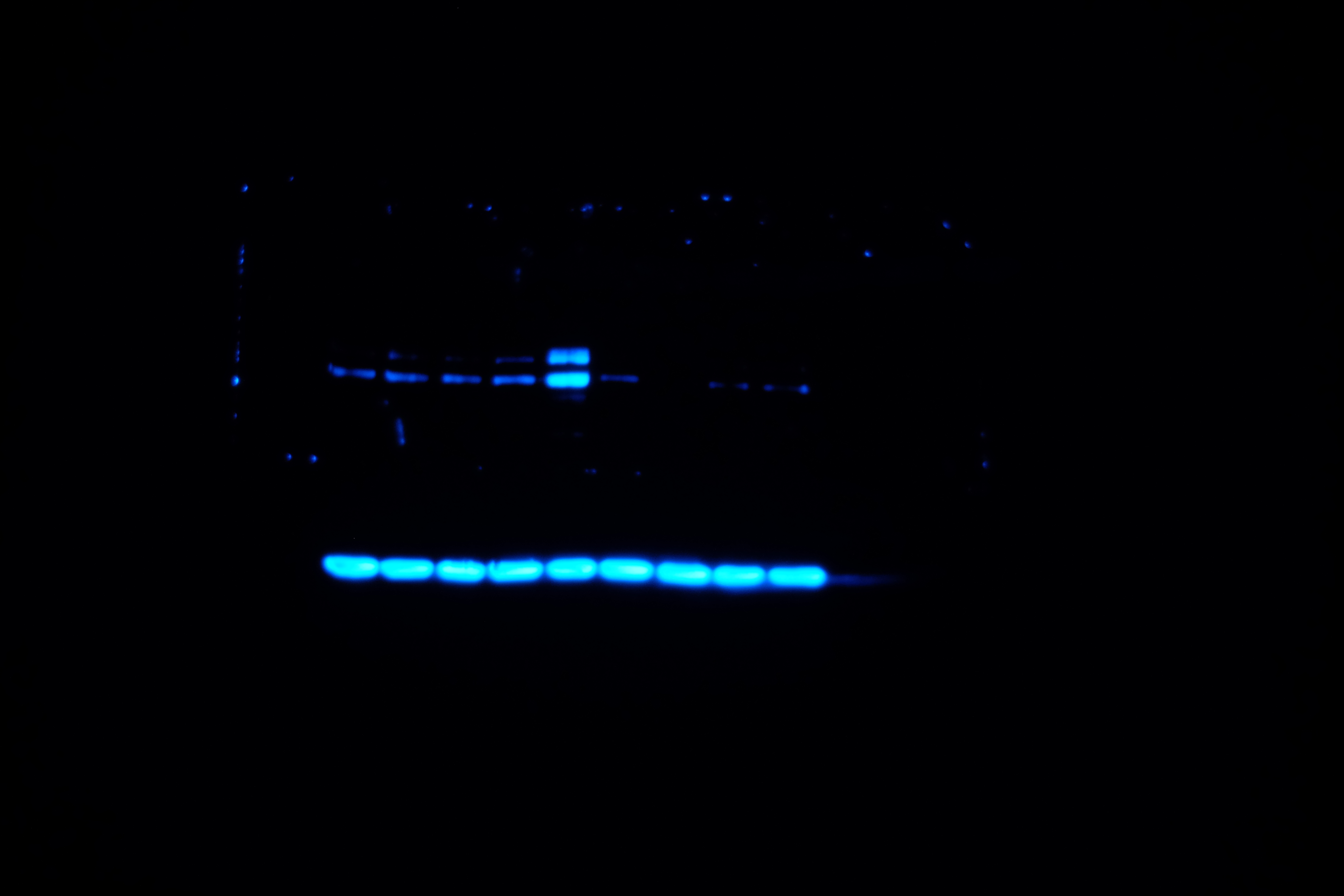

Supplement: Figure 5—source data 1. [file elife-78163-fig5-data1.zip › Figure 5-source data 1/Fig.5B_HCFC1.JPG]

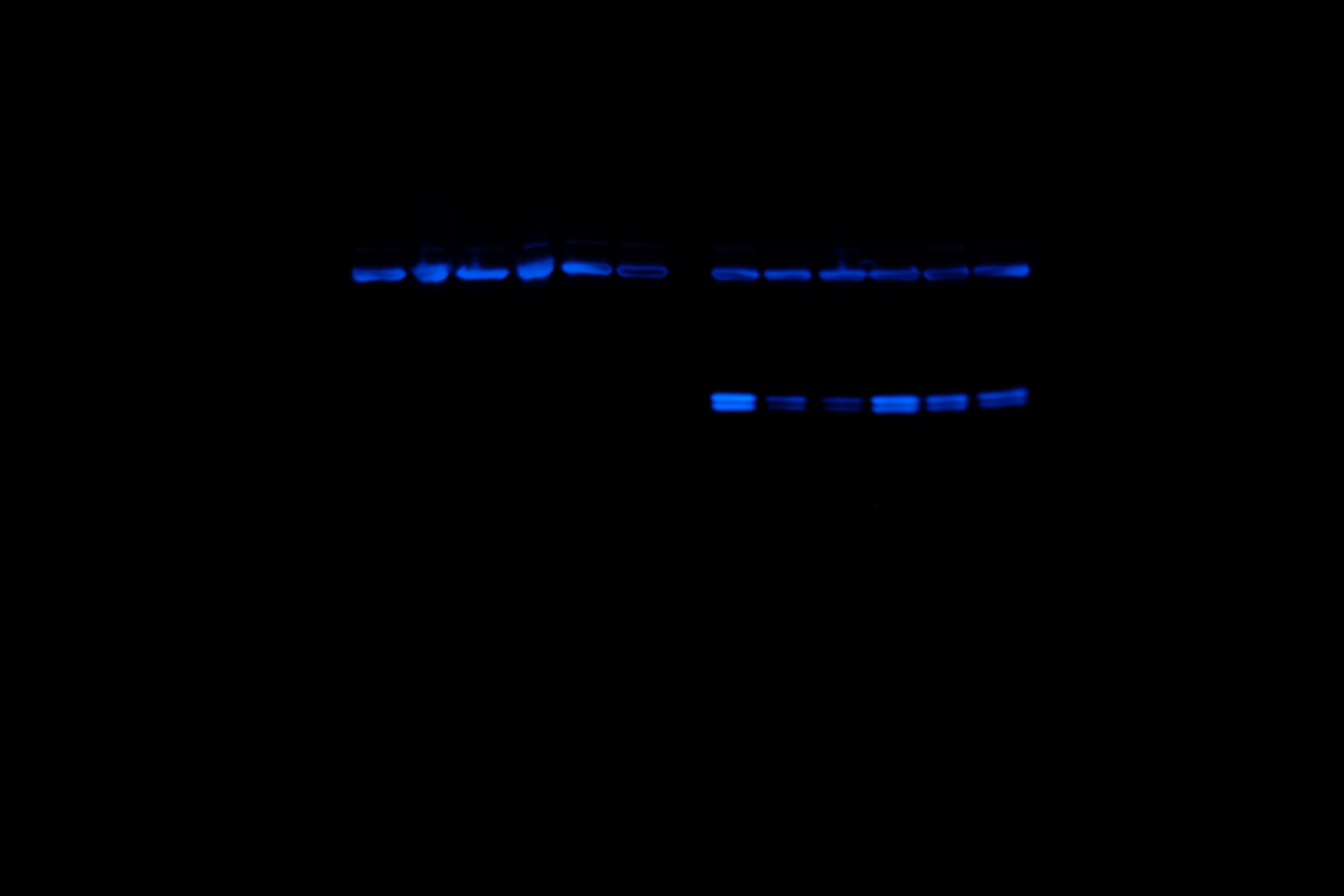

Supplement: Figure 5—source data 1. [file elife-78163-fig5-data1.zip › Figure 5-source data 1/Fig.5B_RBBP5.JPG]

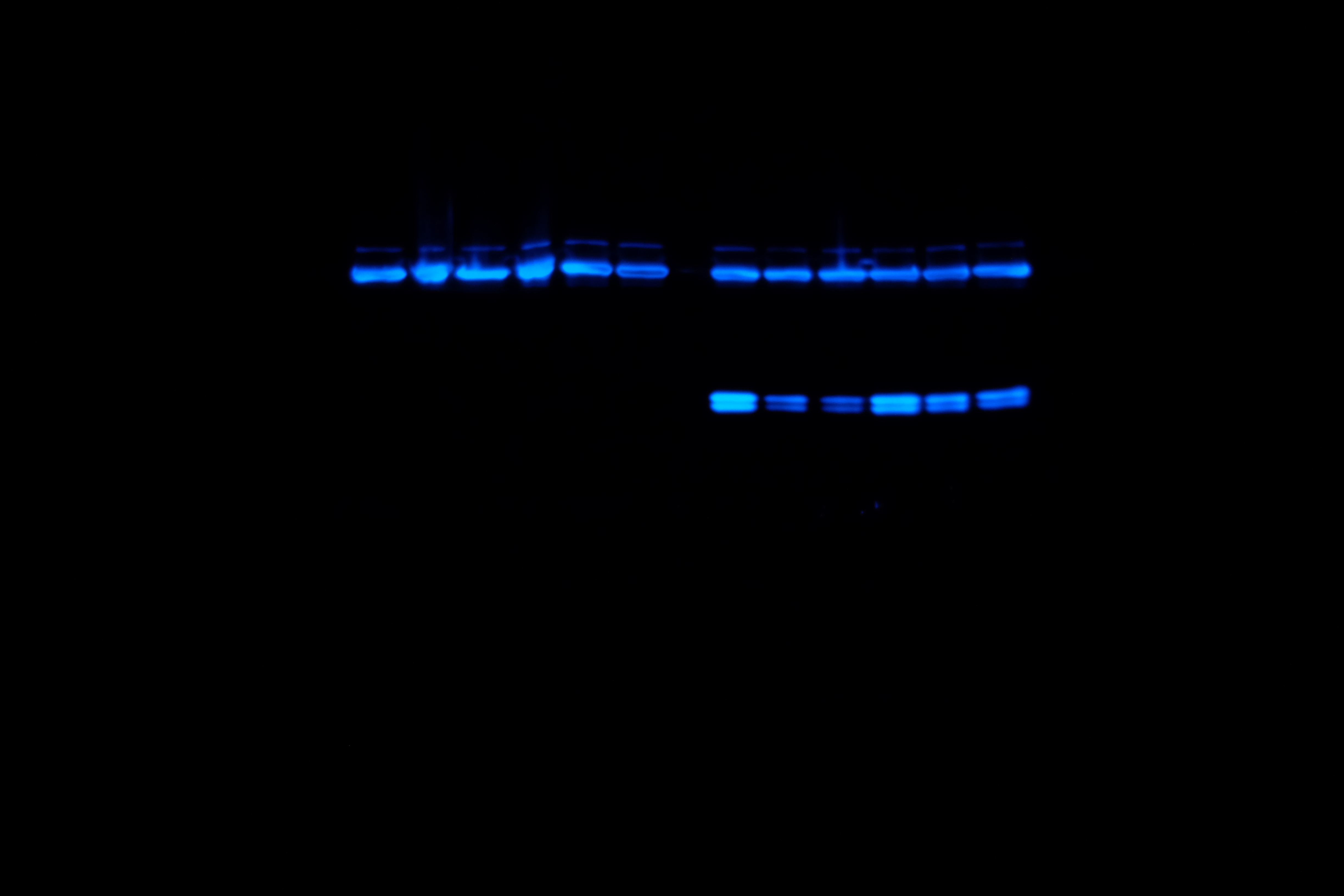

Supplement: Figure 5—source data 1. [file elife-78163-fig5-data1.zip › Figure 5-source data 1/Fig.5B_Vinculin.JPG]

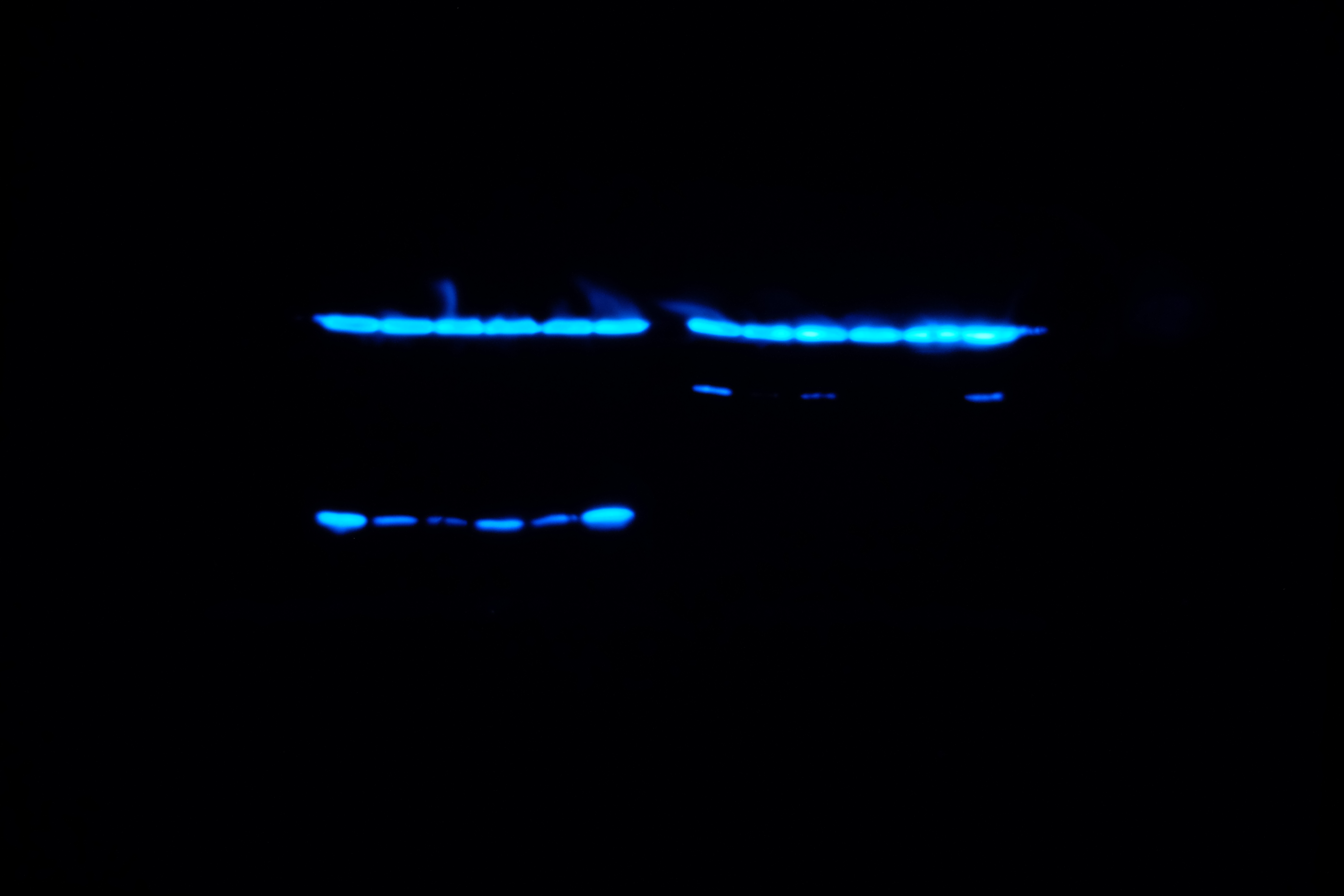

Supplement: Figure 5—source data 1. [file elife-78163-fig5-data1.zip › Figure 5-source data 1/Fig.5B_WDR82.JPG]

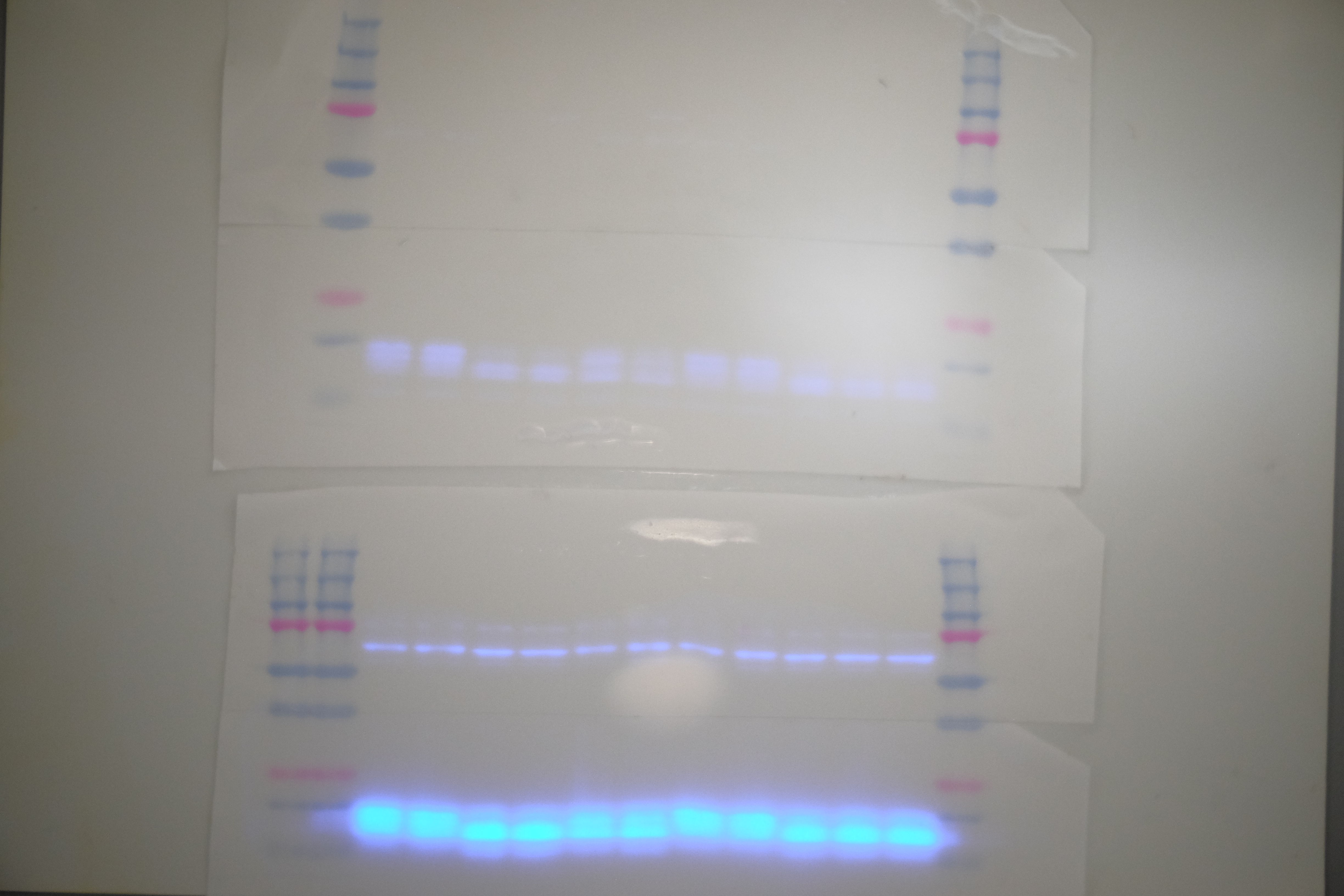

Supplement: Figure 6—source data 1. [file elife-78163-fig6-data1.zip › Figure 6-source data 1/DSCF3471.JPG]

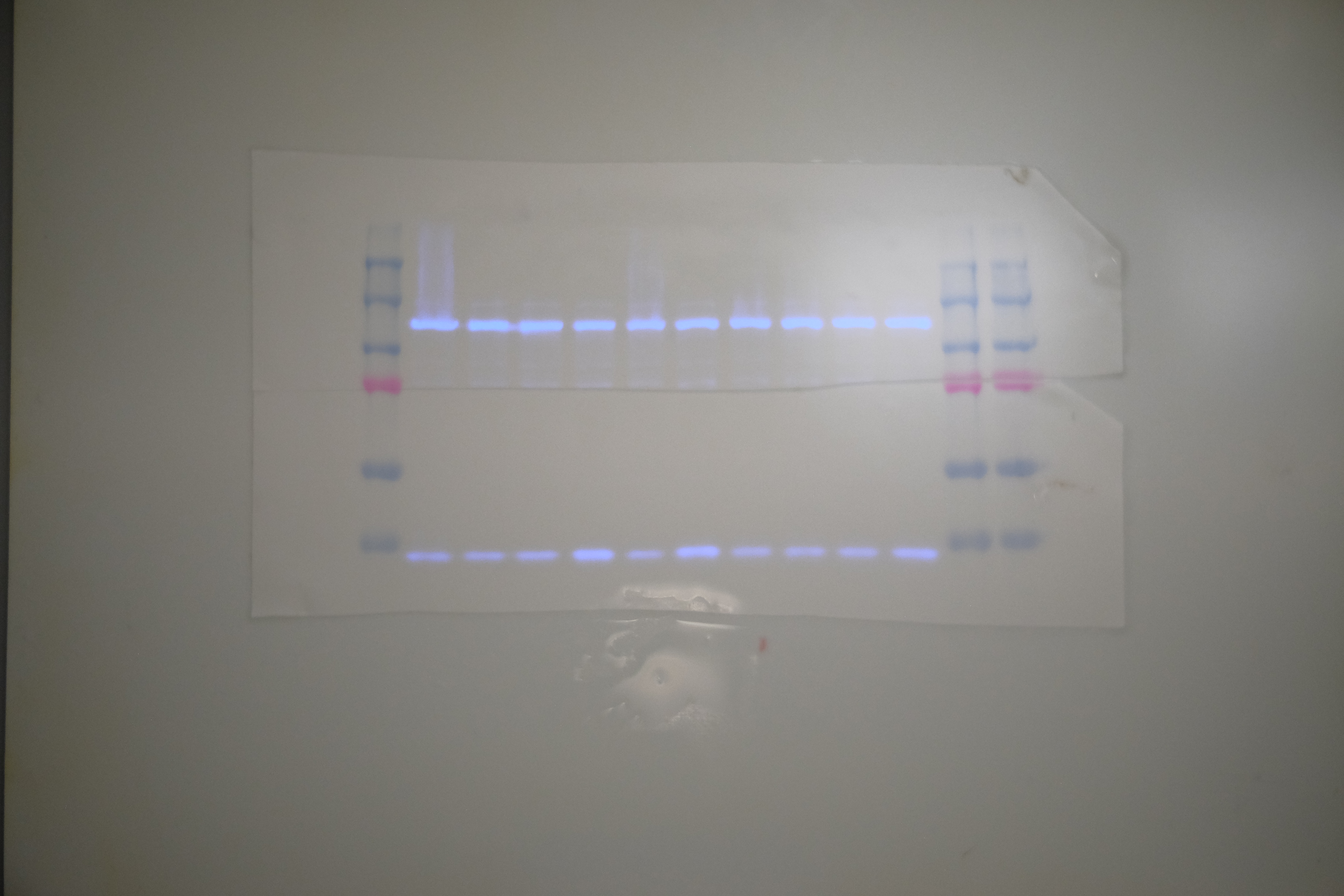

Supplement: Figure 6—source data 1. [file elife-78163-fig6-data1.zip › Figure 6-source data 1/DSCF3475.JPG]

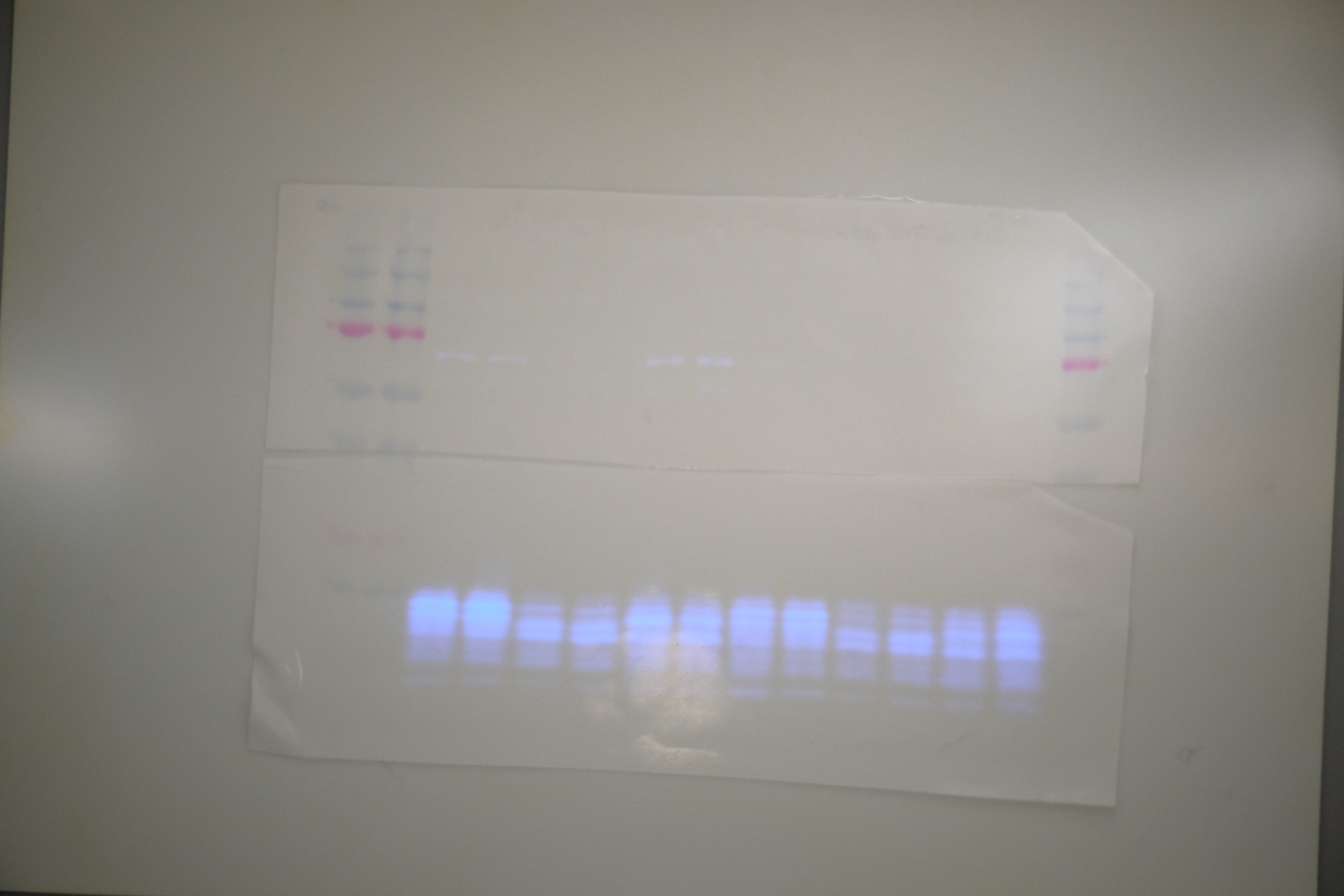

Supplement: Figure 6—source data 1. [file elife-78163-fig6-data1.zip › Figure 6-source data 1/DSCF3538.JPG]

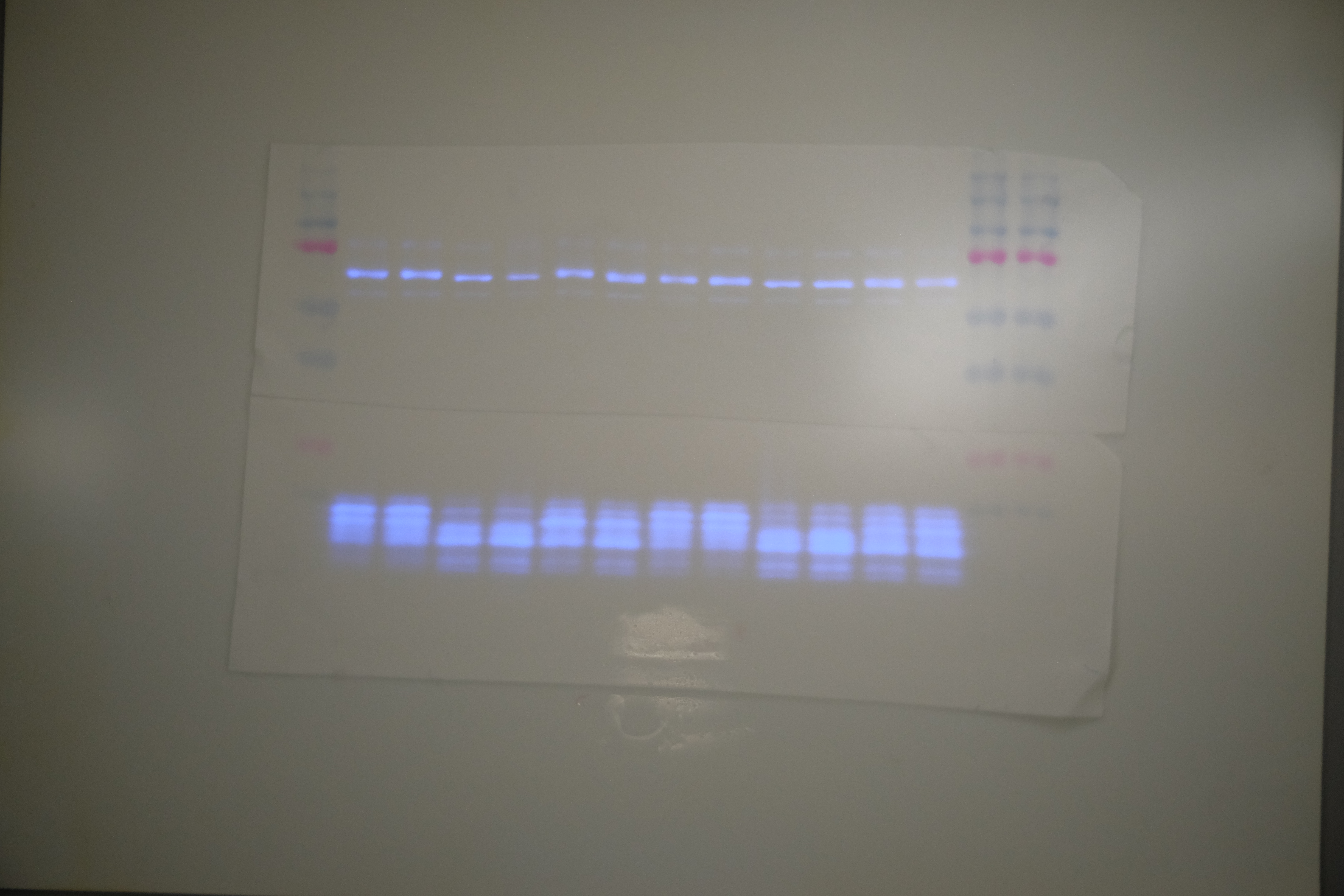

Supplement: Figure 6—source data 1. [file elife-78163-fig6-data1.zip › Figure 6-source data 1/DSCF3543.JPG]

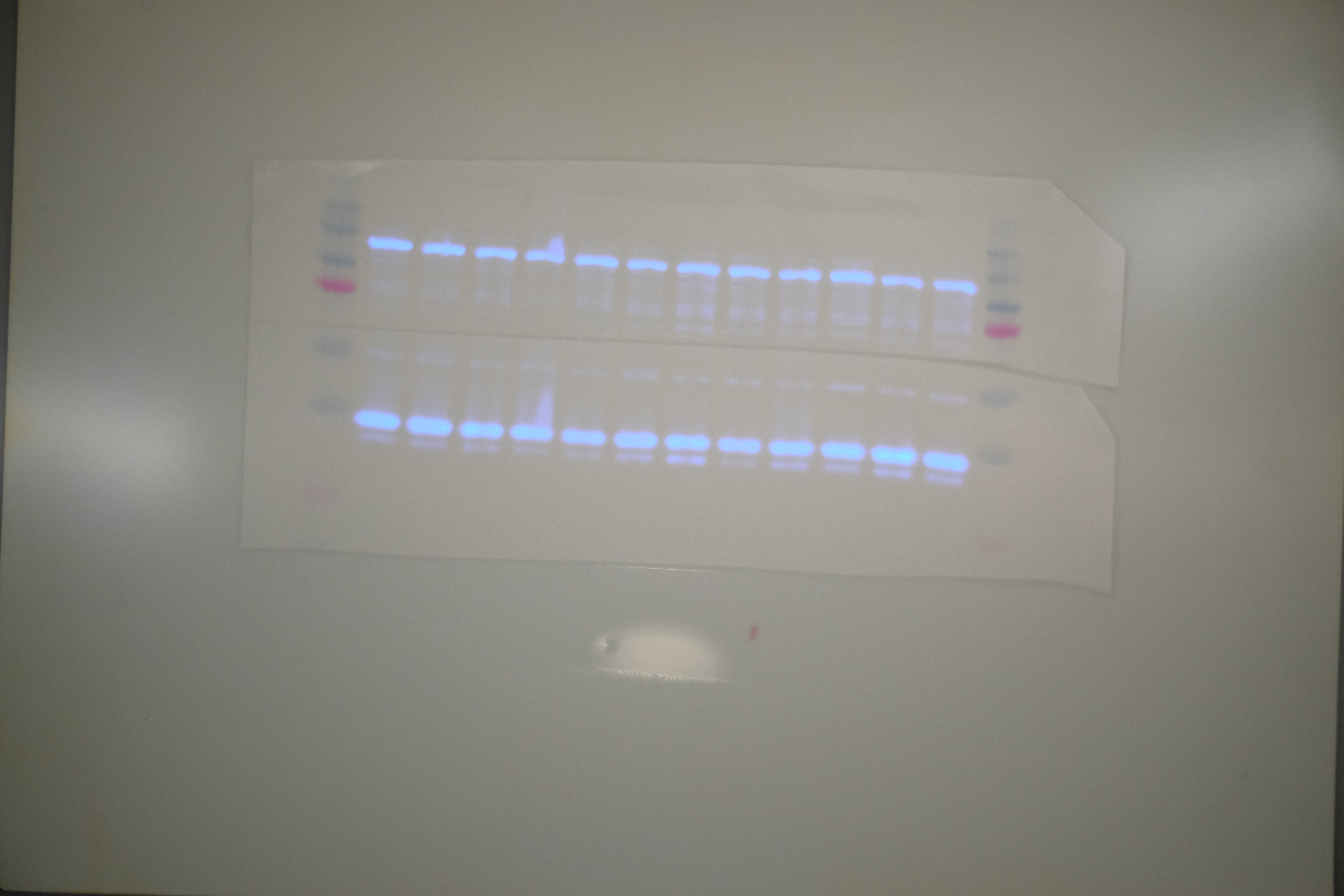

Supplement: Figure 6—source data 1. [file elife-78163-fig6-data1.zip › Figure 6-source data 1/DSCF3548.JPG]

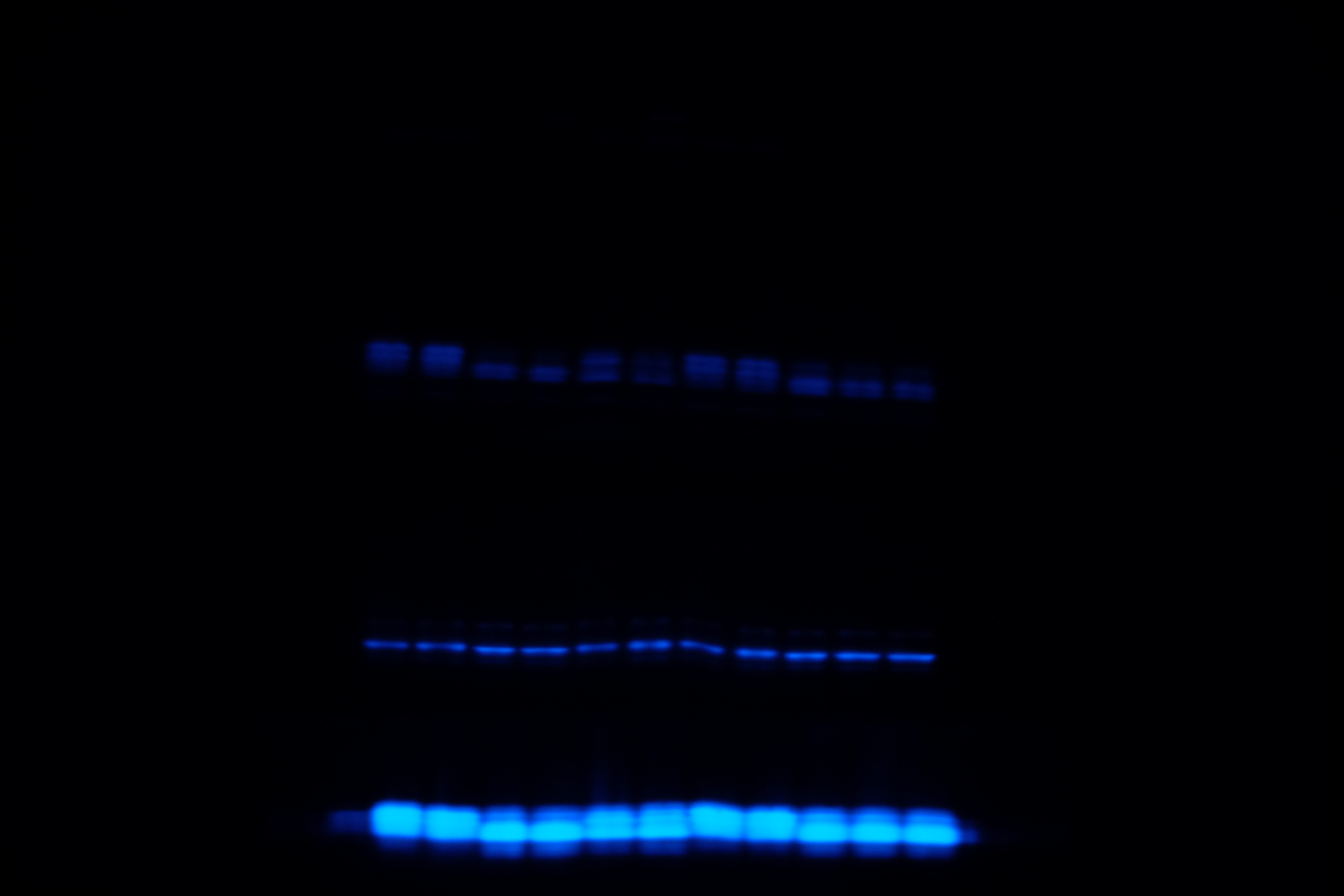

Supplement: Figure 6—source data 1. [file elife-78163-fig6-data1.zip › Figure 6-source data 1/Fig.6A_p-4E-BP1_4E-BP1.JPG]

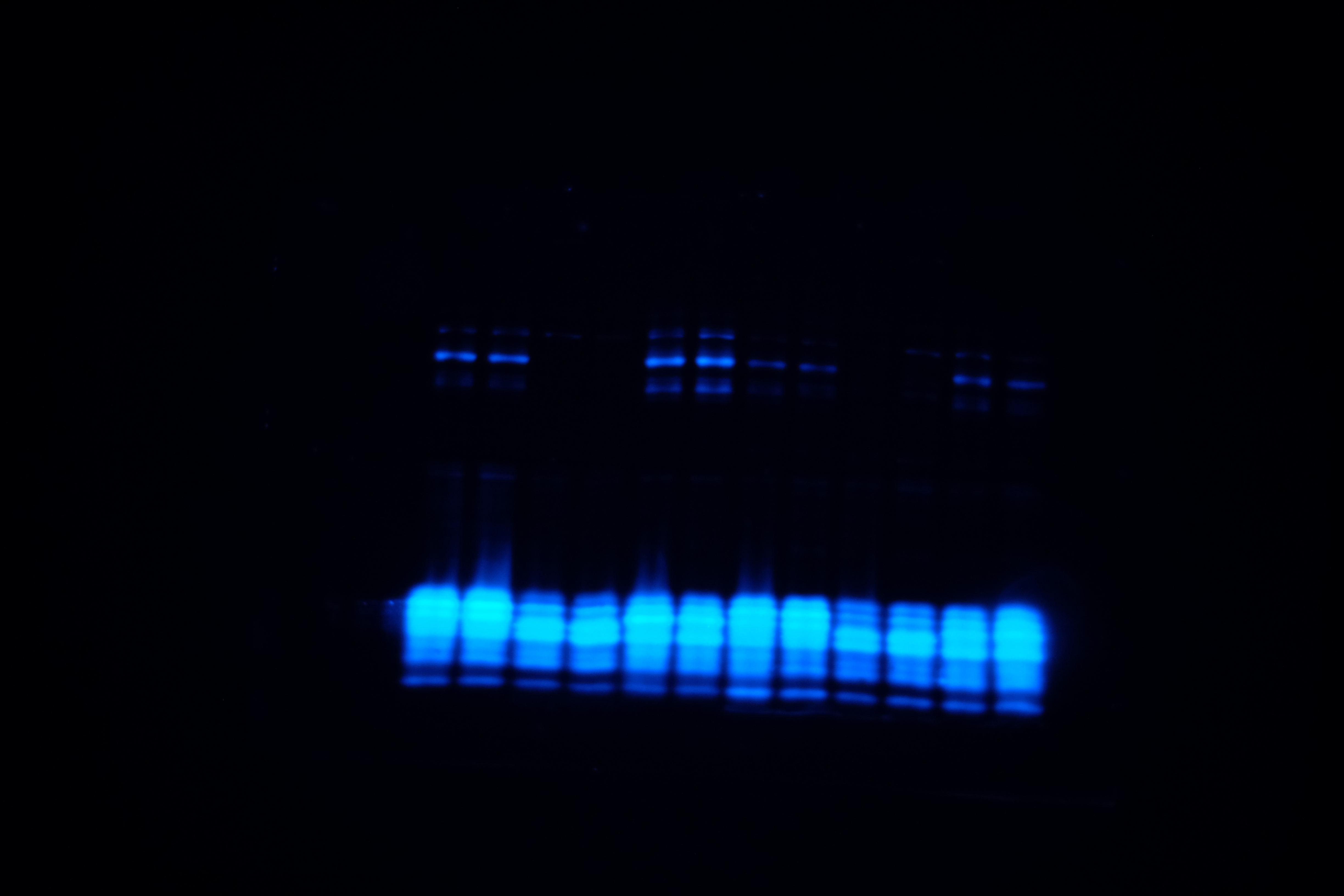

Supplement: Figure 6—source data 1. [file elife-78163-fig6-data1.zip › Figure 6-source data 1/Fig.6A_p-S6K.JPG]

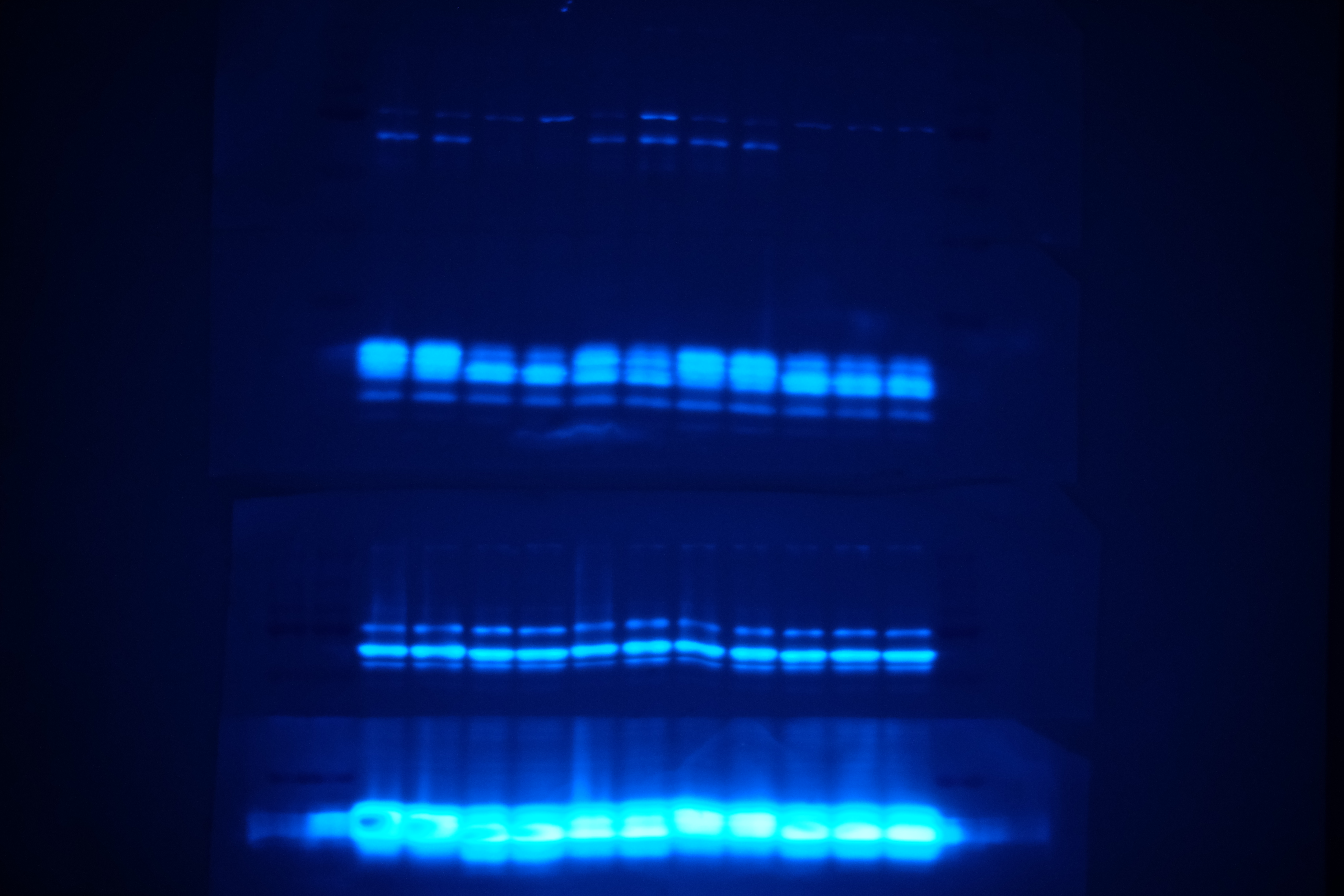

Supplement: Figure 6—source data 1. [file elife-78163-fig6-data1.zip › Figure 6-source data 1/Fig.6A_p-S6K_everolimus.JPG]

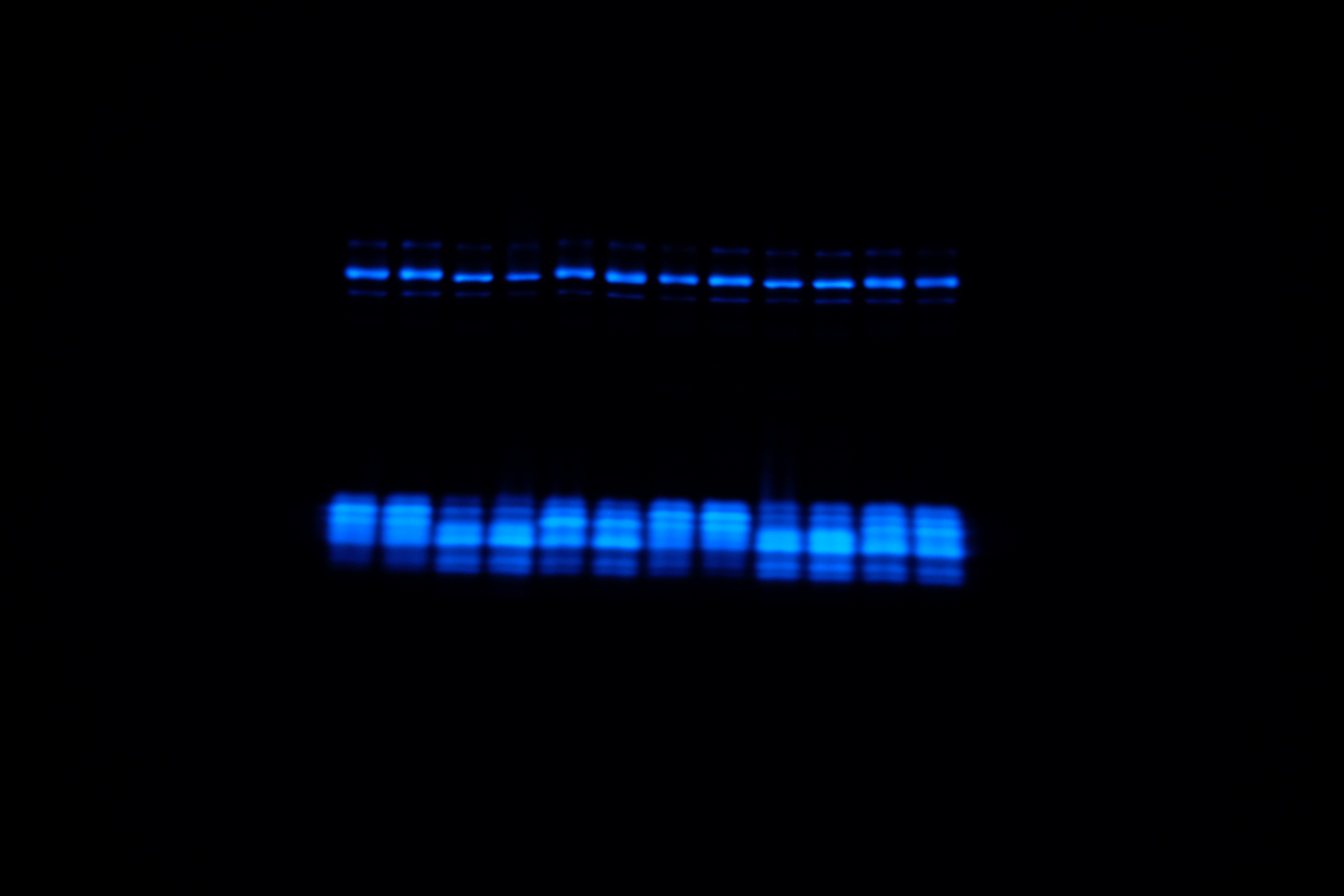

Supplement: Figure 6—source data 1. [file elife-78163-fig6-data1.zip › Figure 6-source data 1/Fig.6A_S6K.JPG]

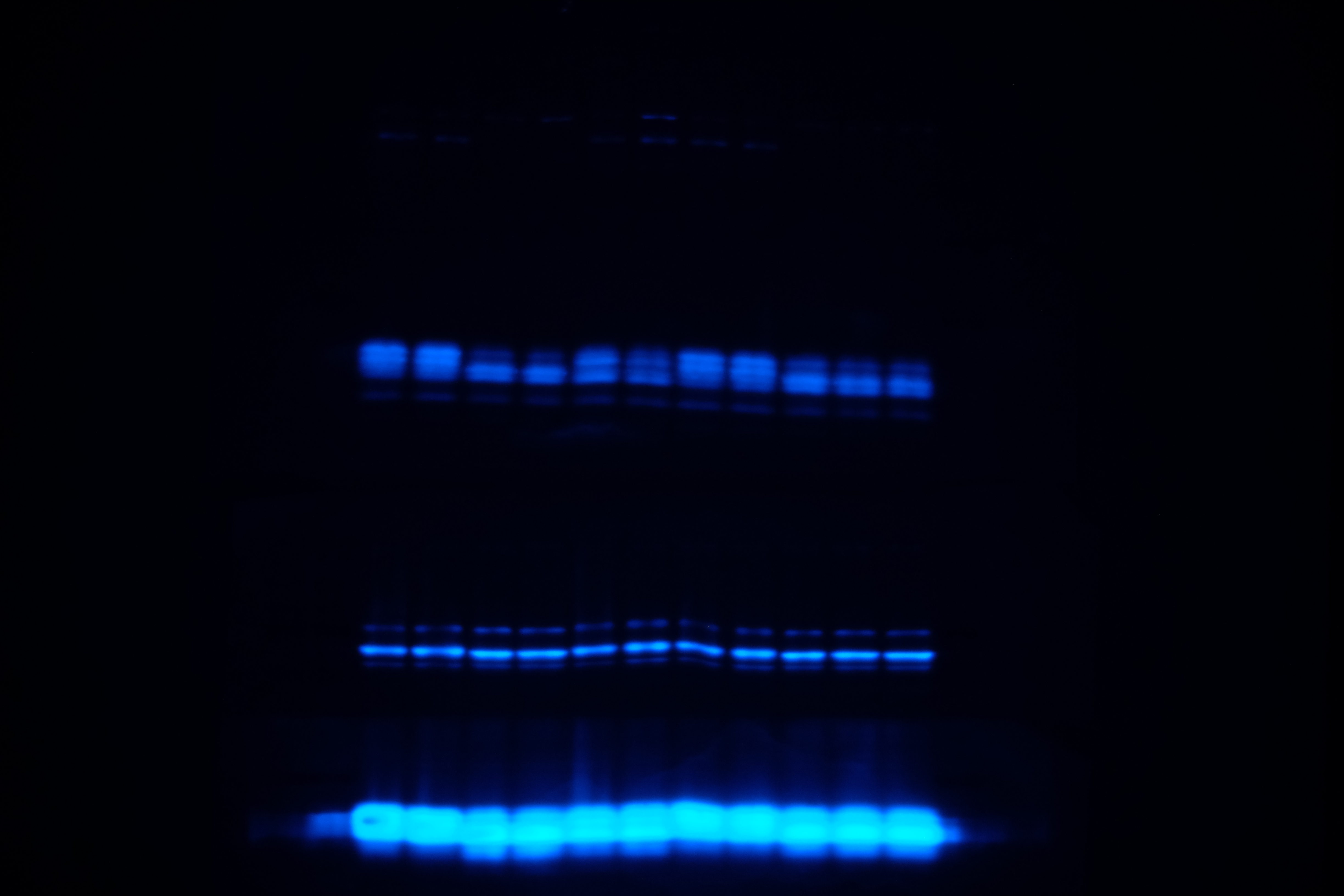

Supplement: Figure 6—source data 1. [file elife-78163-fig6-data1.zip › Figure 6-source data 1/Fig.6A_S6K_everolimus.JPG]

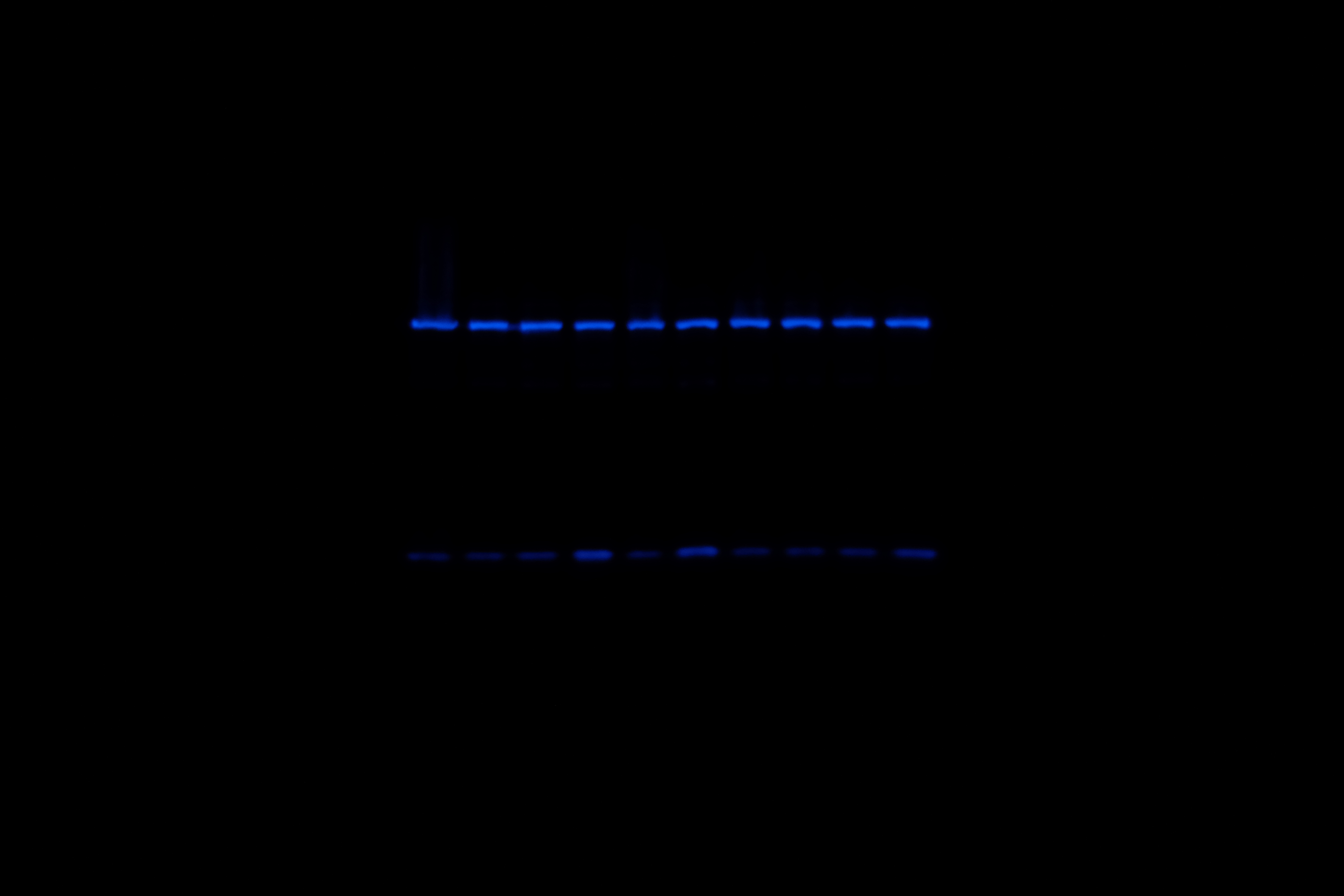

Supplement: Figure 6—source data 1. [file elife-78163-fig6-data1.zip › Figure 6-source data 1/Fig.6A_vinculin.JPG]

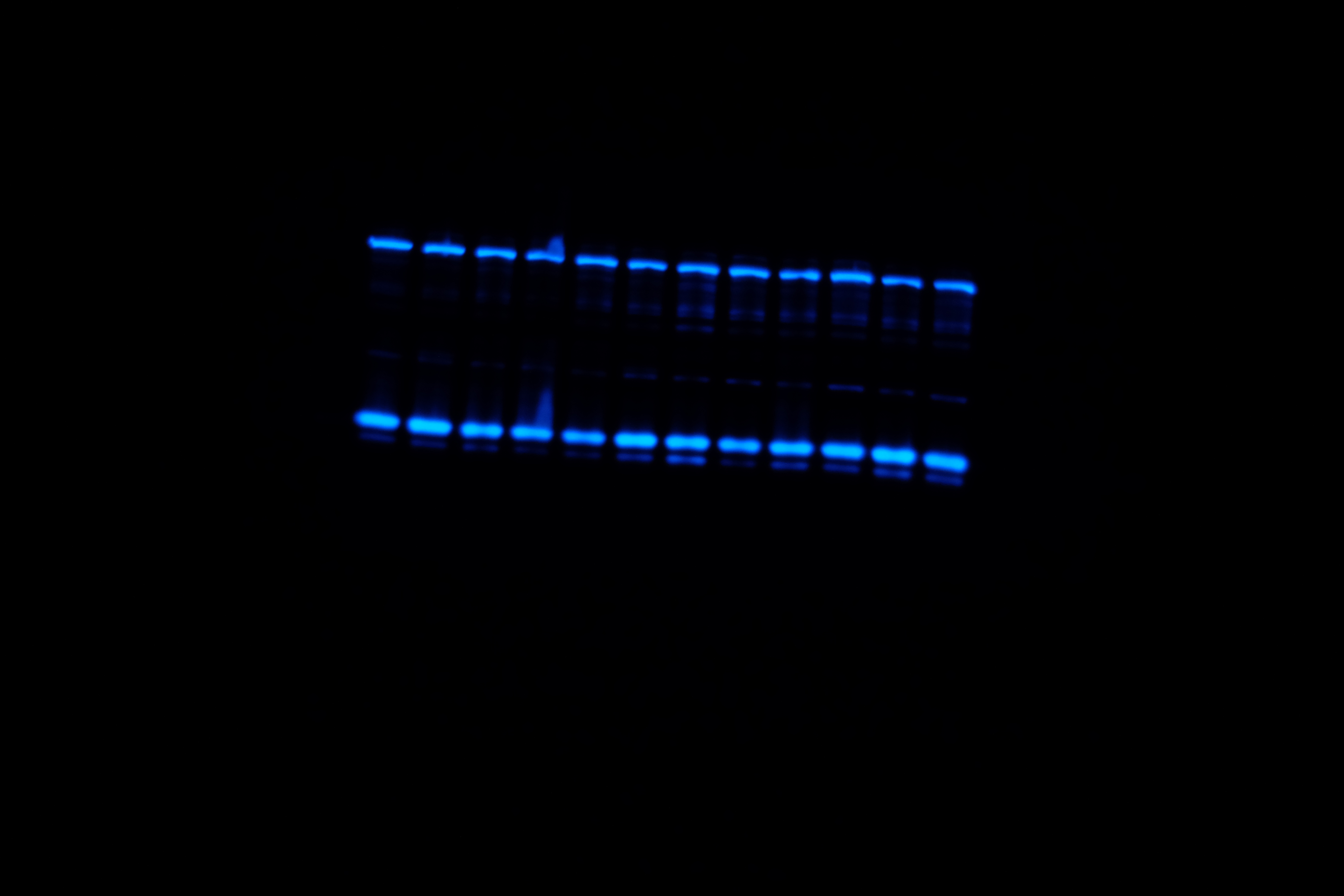

Supplement: Figure 6—source data 1. [file elife-78163-fig6-data1.zip › Figure 6-source data 1/Fig.6A_WDR5.JPG]

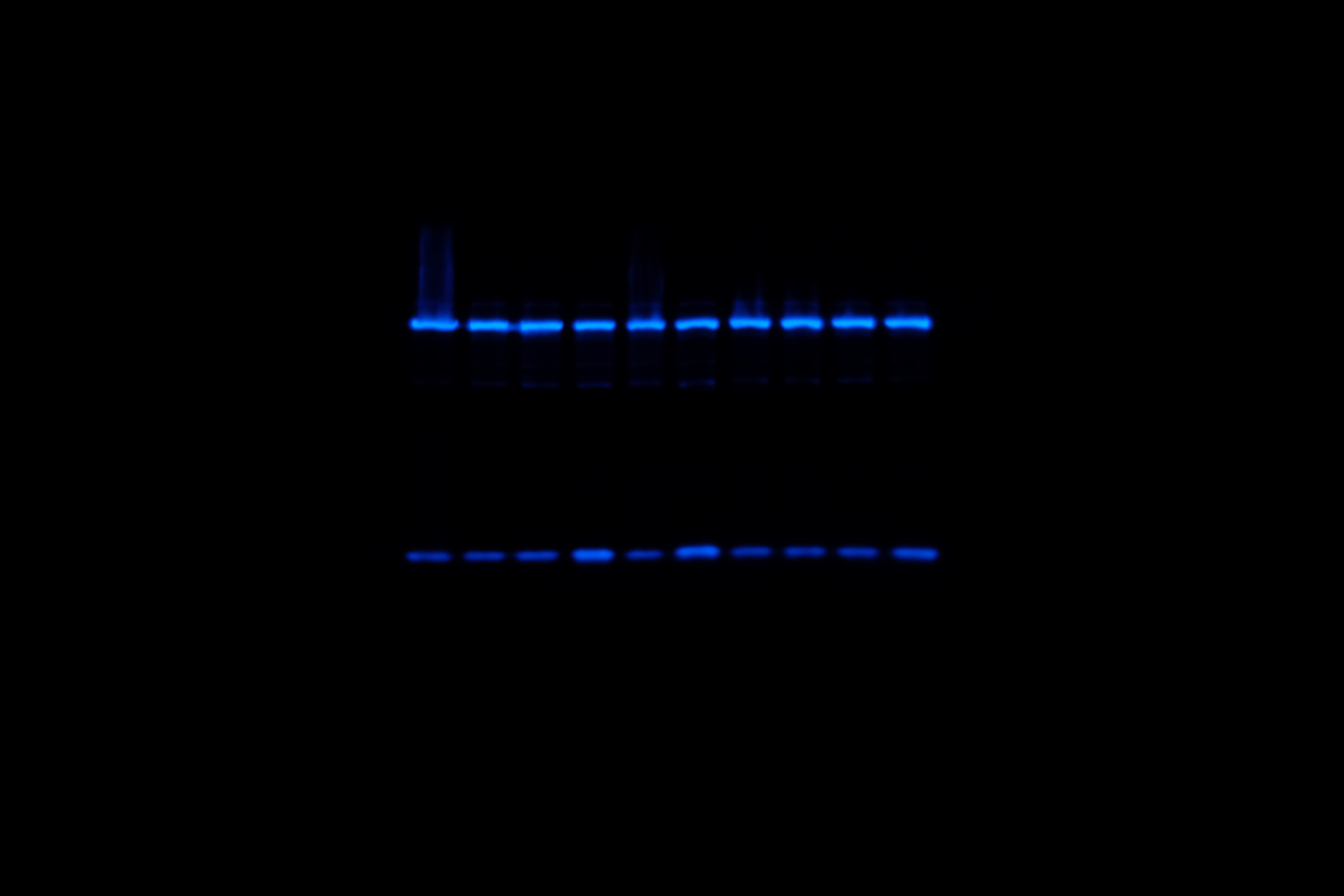

Supplement: Figure 6—source data 1. [file elife-78163-fig6-data1.zip › Figure 6-source data 1/Fig.6A_WDR5_everolimus.JPG]

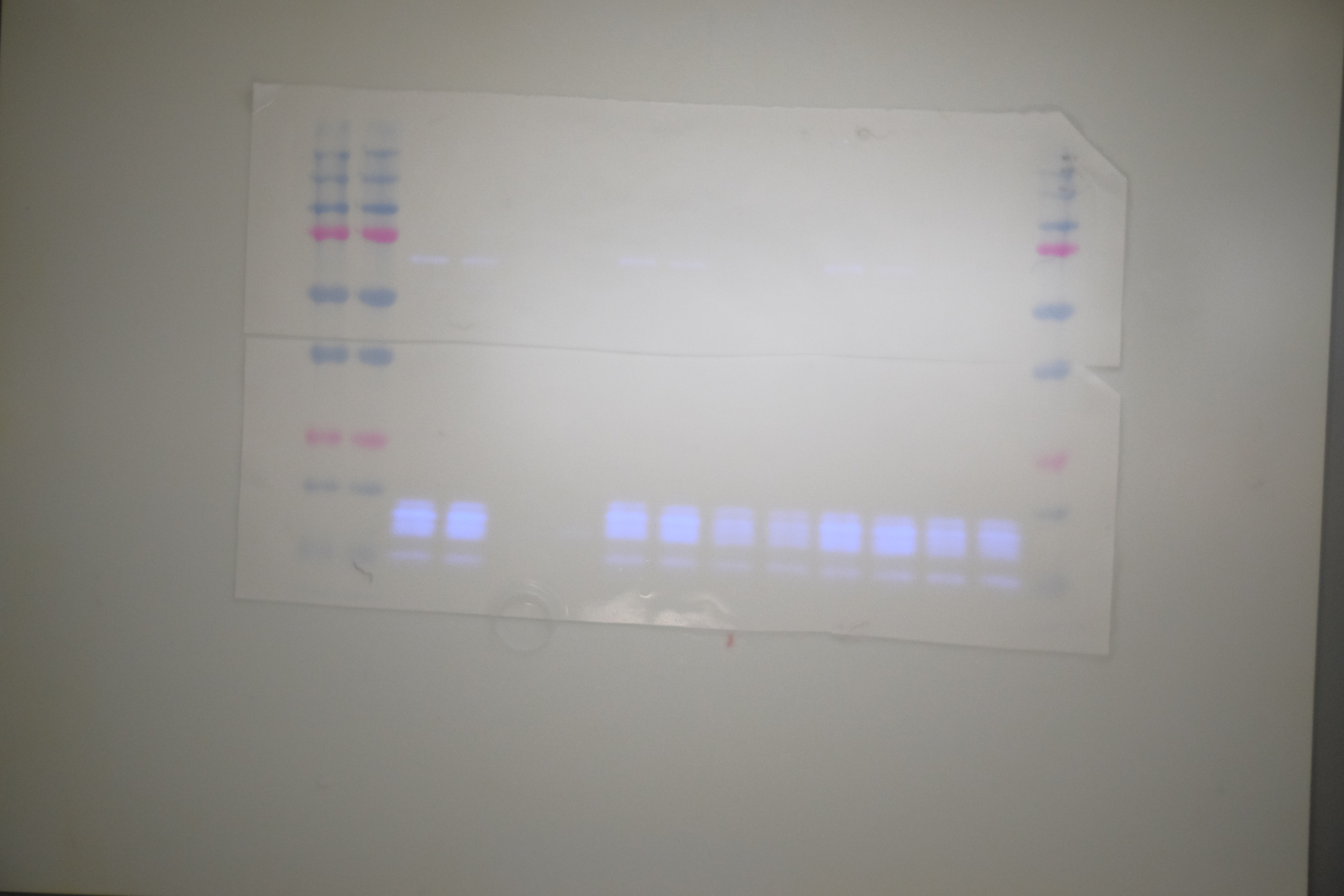

Supplement: Figure 6—source data 2. [file elife-78163-fig6-data2.zip › Figure 6-source data 2/DSCF3640.JPG]

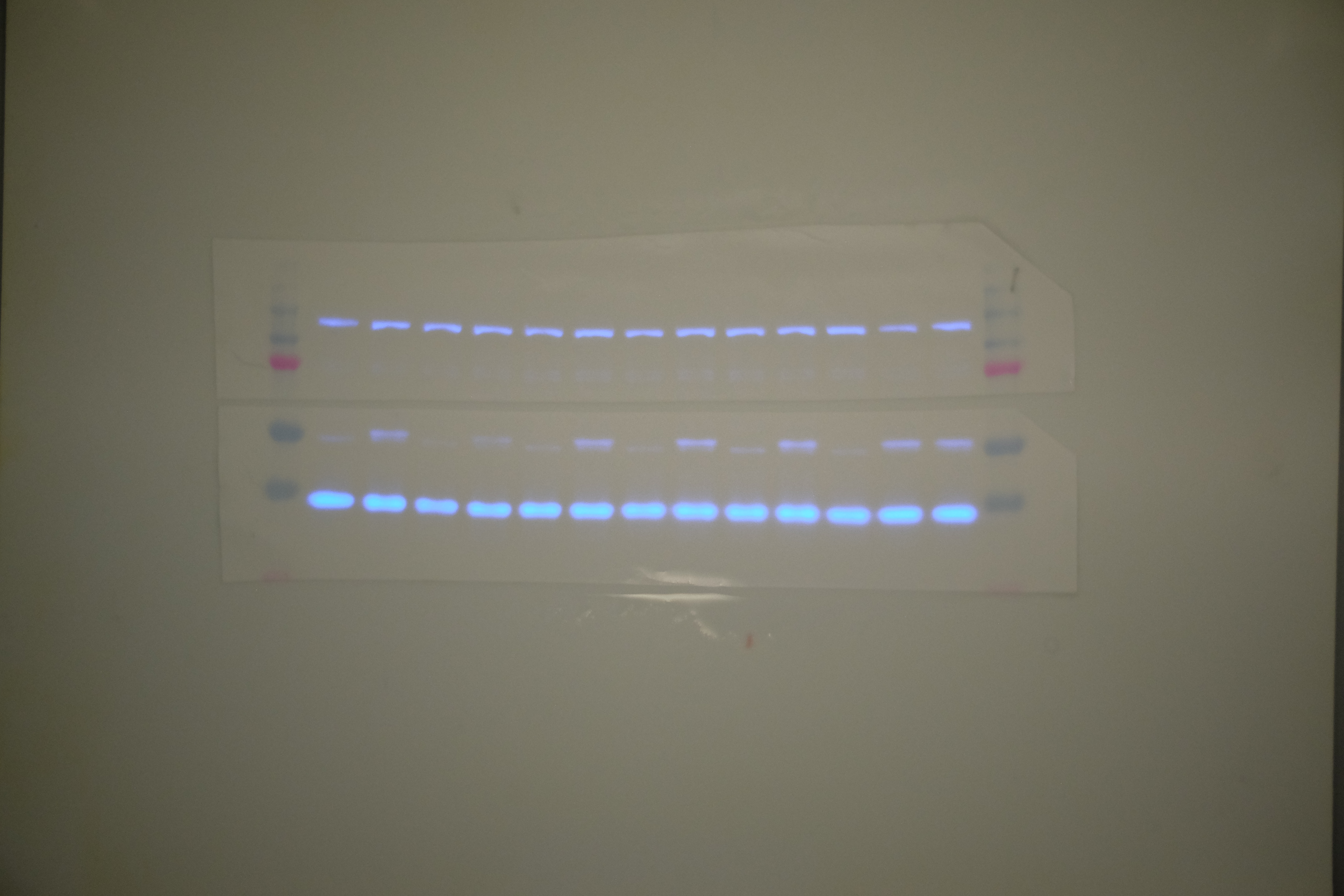

Supplement: Figure 6—source data 2. [file elife-78163-fig6-data2.zip › Figure 6-source data 2/DSCF3643.JPG]

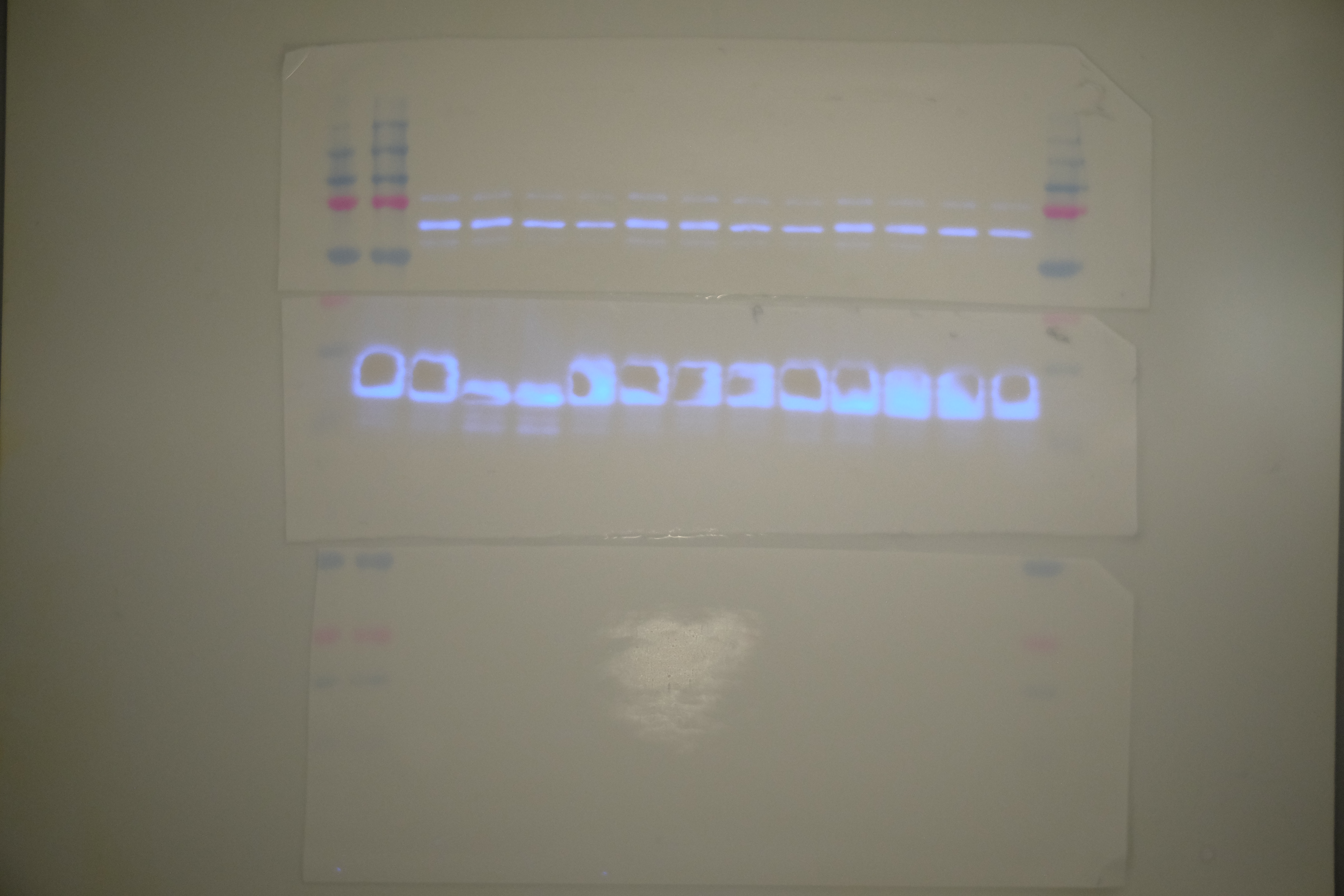

Supplement: Figure 6—source data 2. [file elife-78163-fig6-data2.zip › Figure 6-source data 2/DSCF3648.JPG]

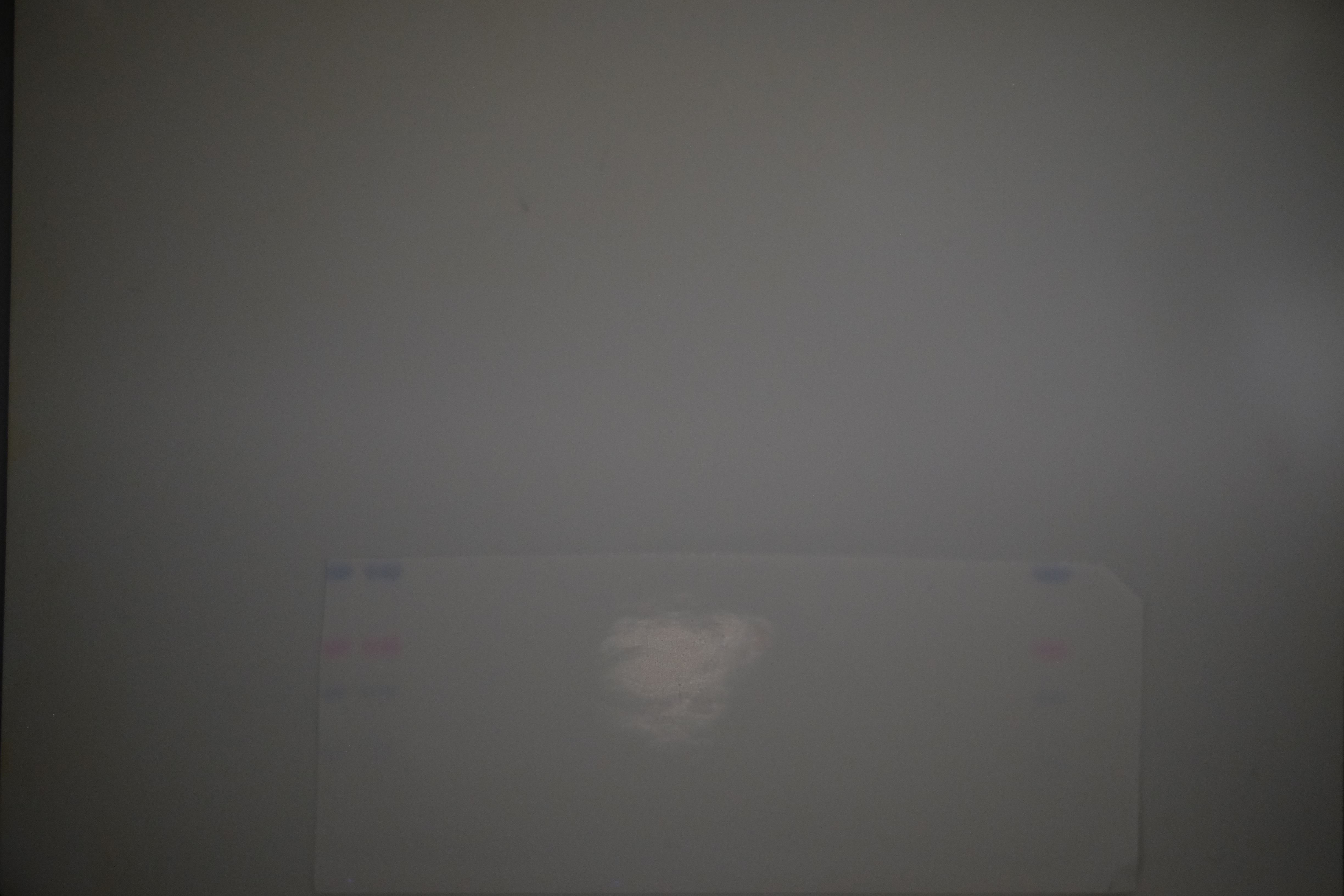

Supplement: Figure 6—source data 2. [file elife-78163-fig6-data2.zip › Figure 6-source data 2/DSCF3651.JPG]

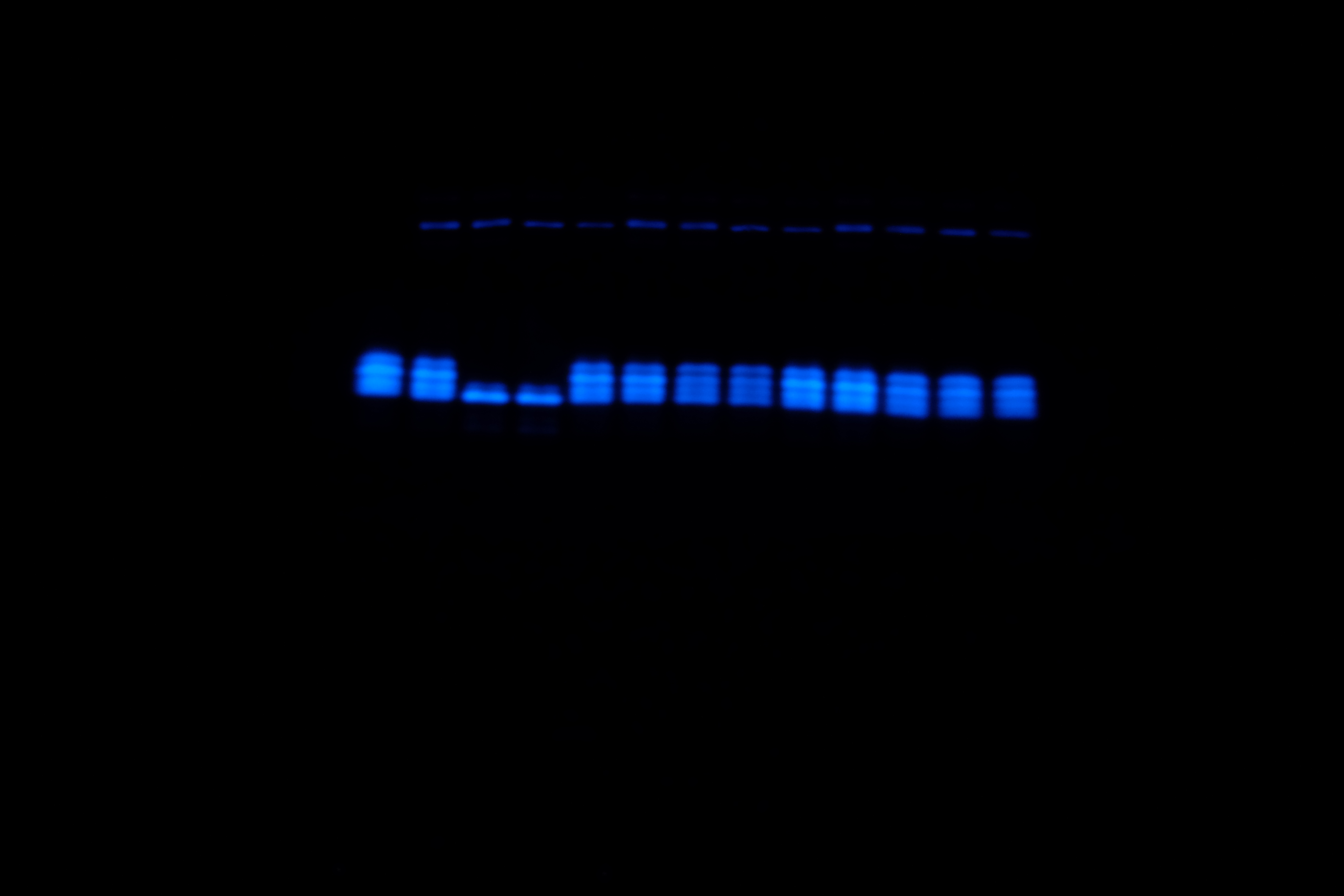

Supplement: Figure 6—source data 2. [file elife-78163-fig6-data2.zip › Figure 6-source data 2/Fig.6B_4E-BP1.JPG]

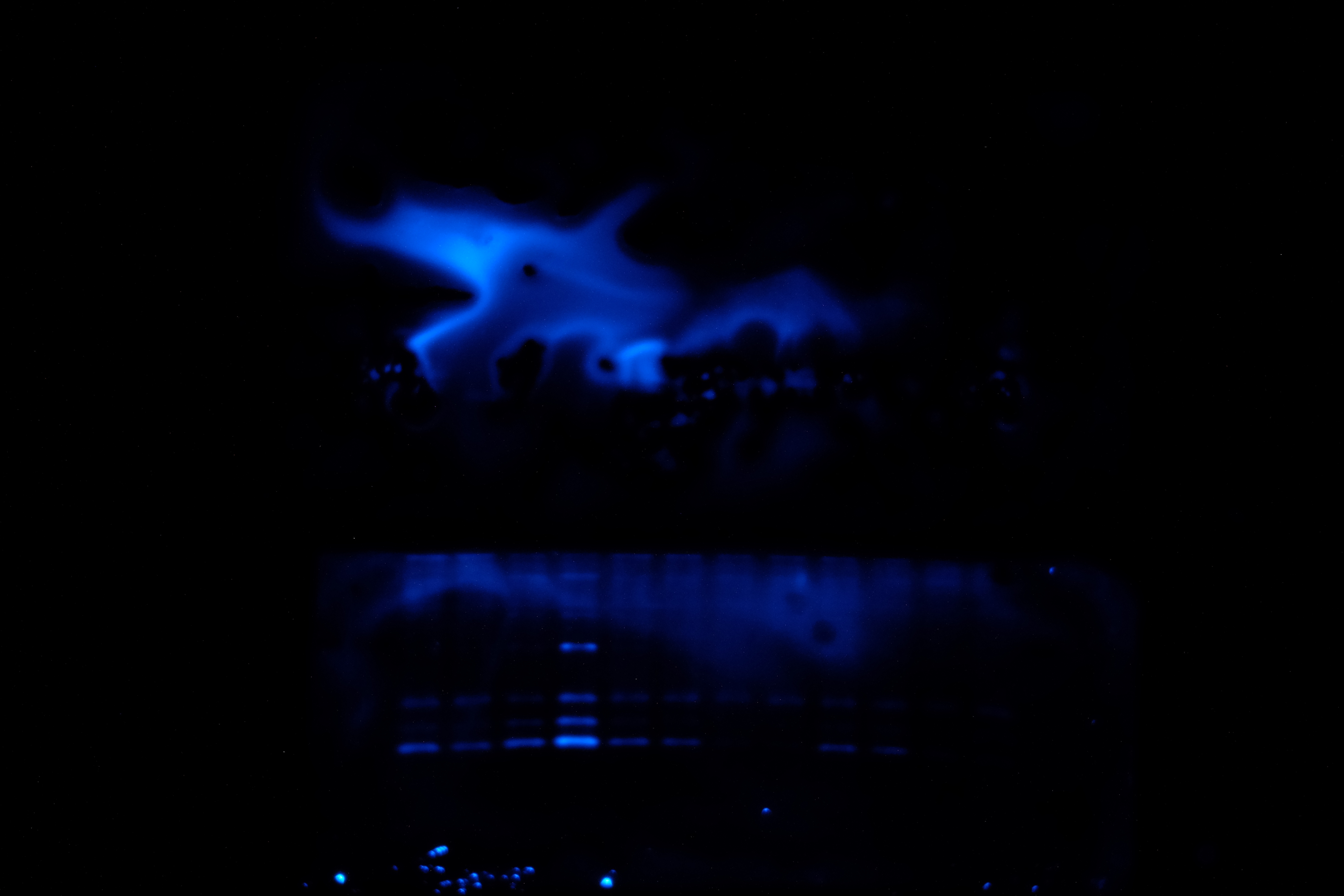

Supplement: Figure 6—source data 2. [file elife-78163-fig6-data2.zip › Figure 6-source data 2/Fig.6B_Cleaved caspase 3.JPG]

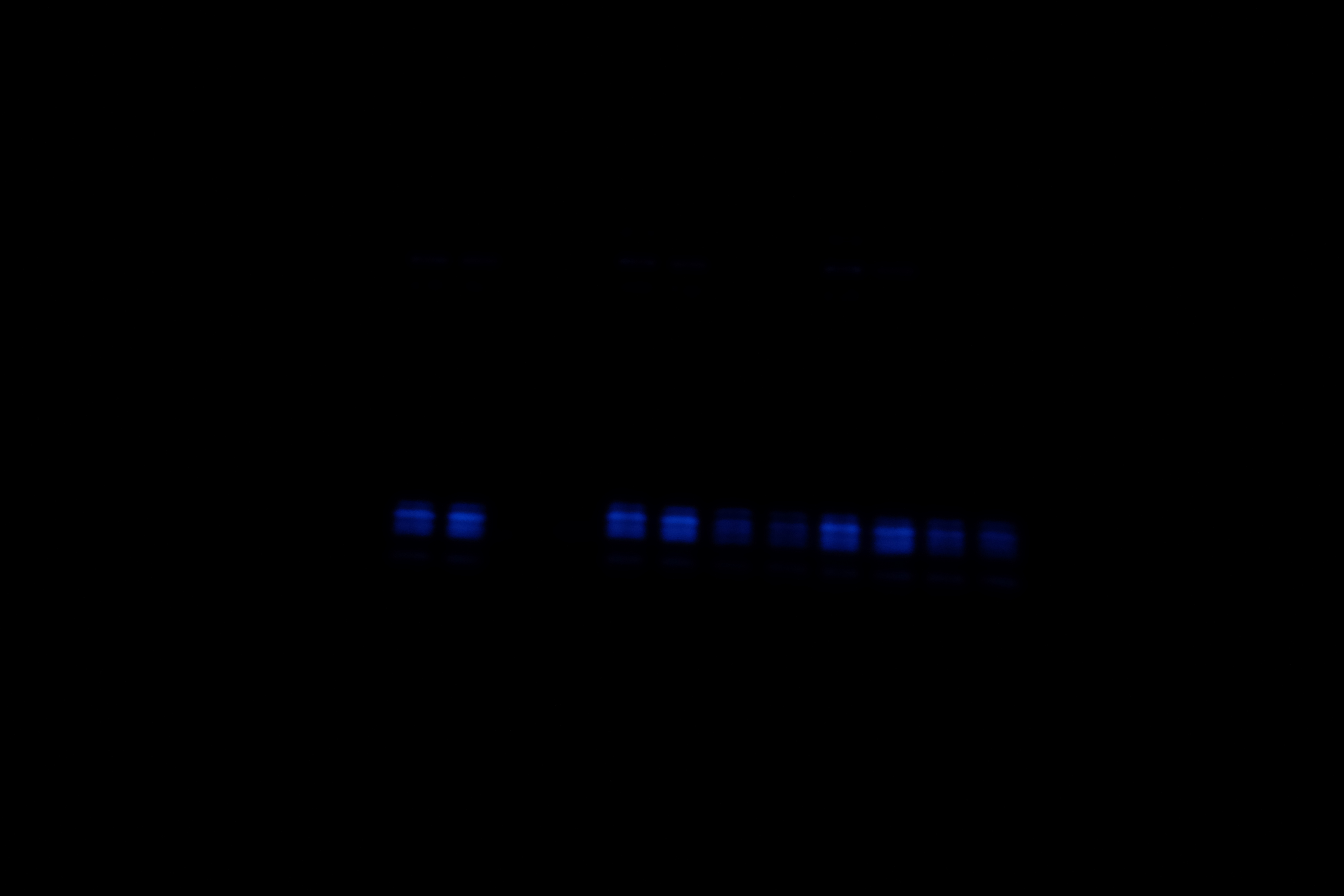

Supplement: Figure 6—source data 2. [file elife-78163-fig6-data2.zip › Figure 6-source data 2/Fig.6B_p-4E-BP1.JPG]

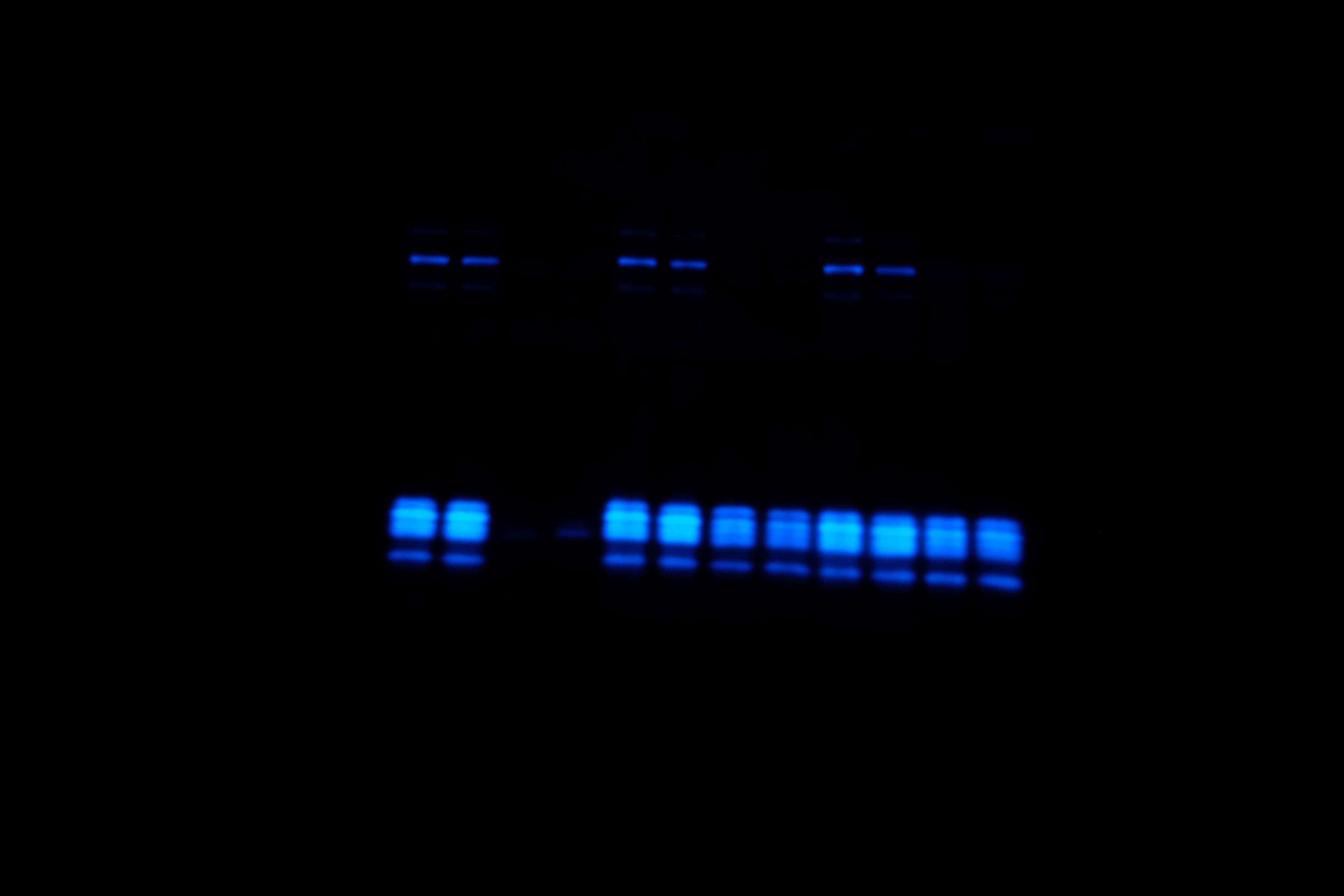

Supplement: Figure 6—source data 2. [file elife-78163-fig6-data2.zip › Figure 6-source data 2/Fig.6B_p-S6K.JPG]

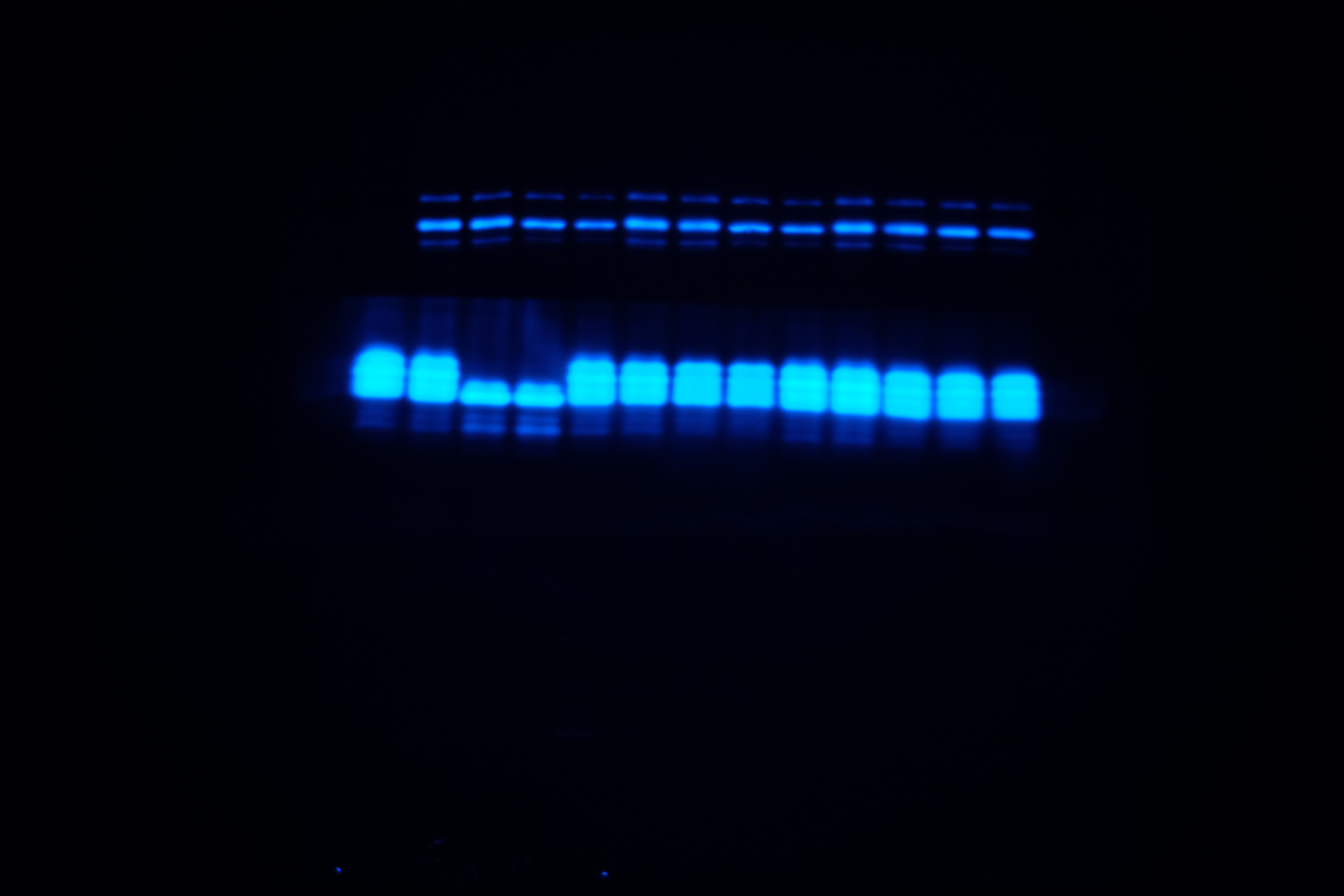

Supplement: Figure 6—source data 2. [file elife-78163-fig6-data2.zip › Figure 6-source data 2/Fig.6B_S6K.JPG]

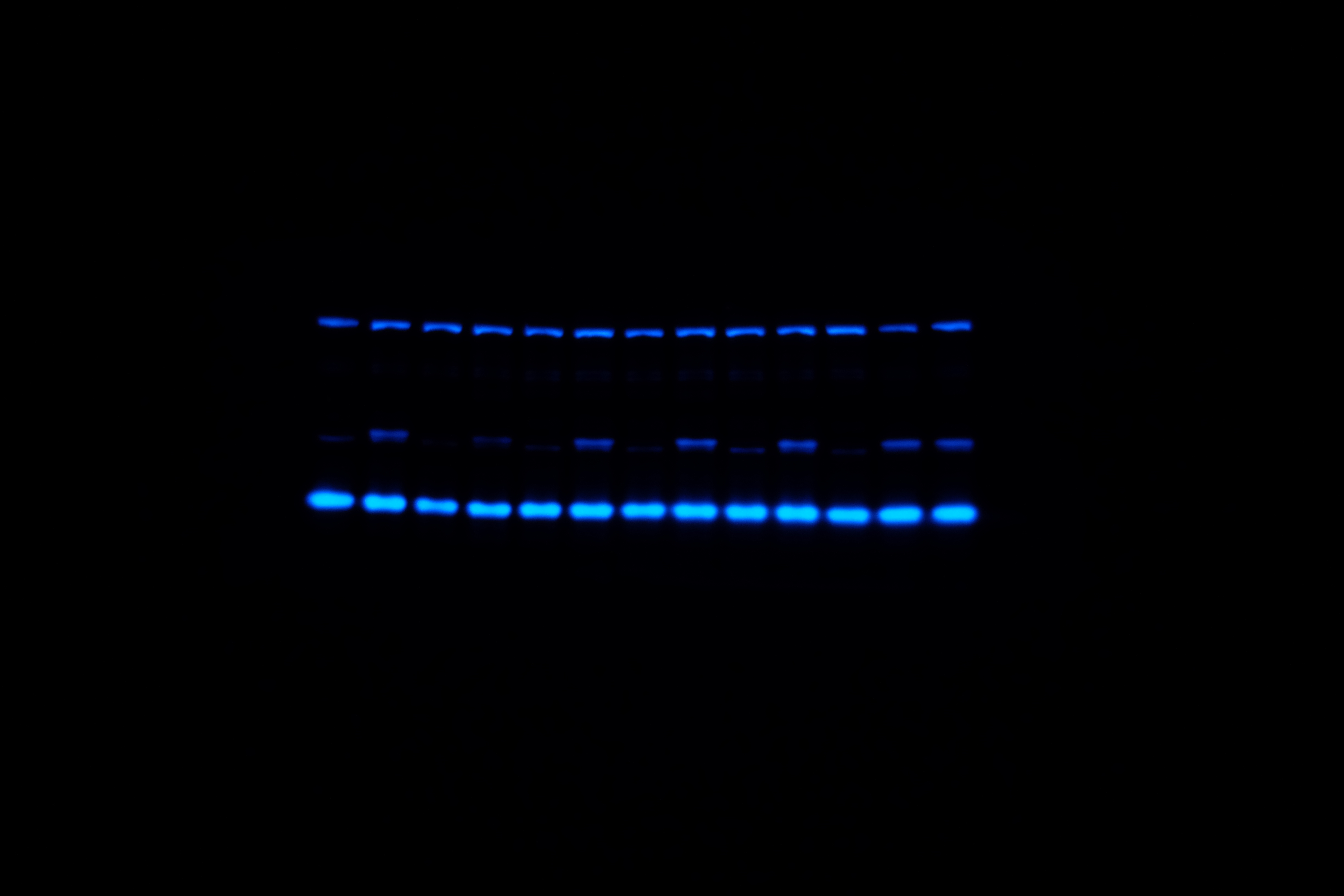

Supplement: Figure 6—source data 2. [file elife-78163-fig6-data2.zip › Figure 6-source data 2/Fig.6B_vinculin.JPG]

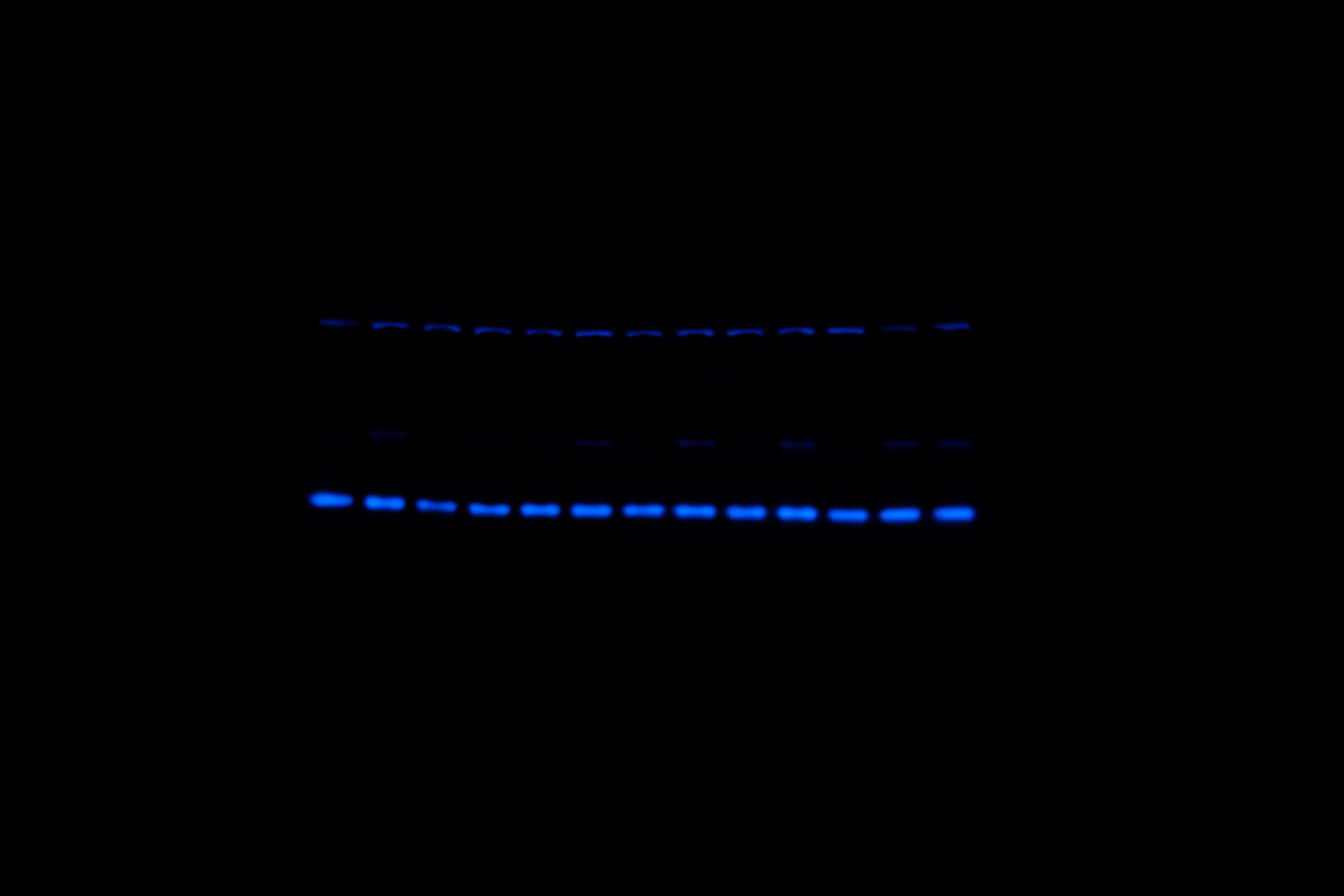

Supplement: Figure 6—source data 2. [file elife-78163-fig6-data2.zip › Figure 6-source data 2/Fig.6B_WDR5.JPG]

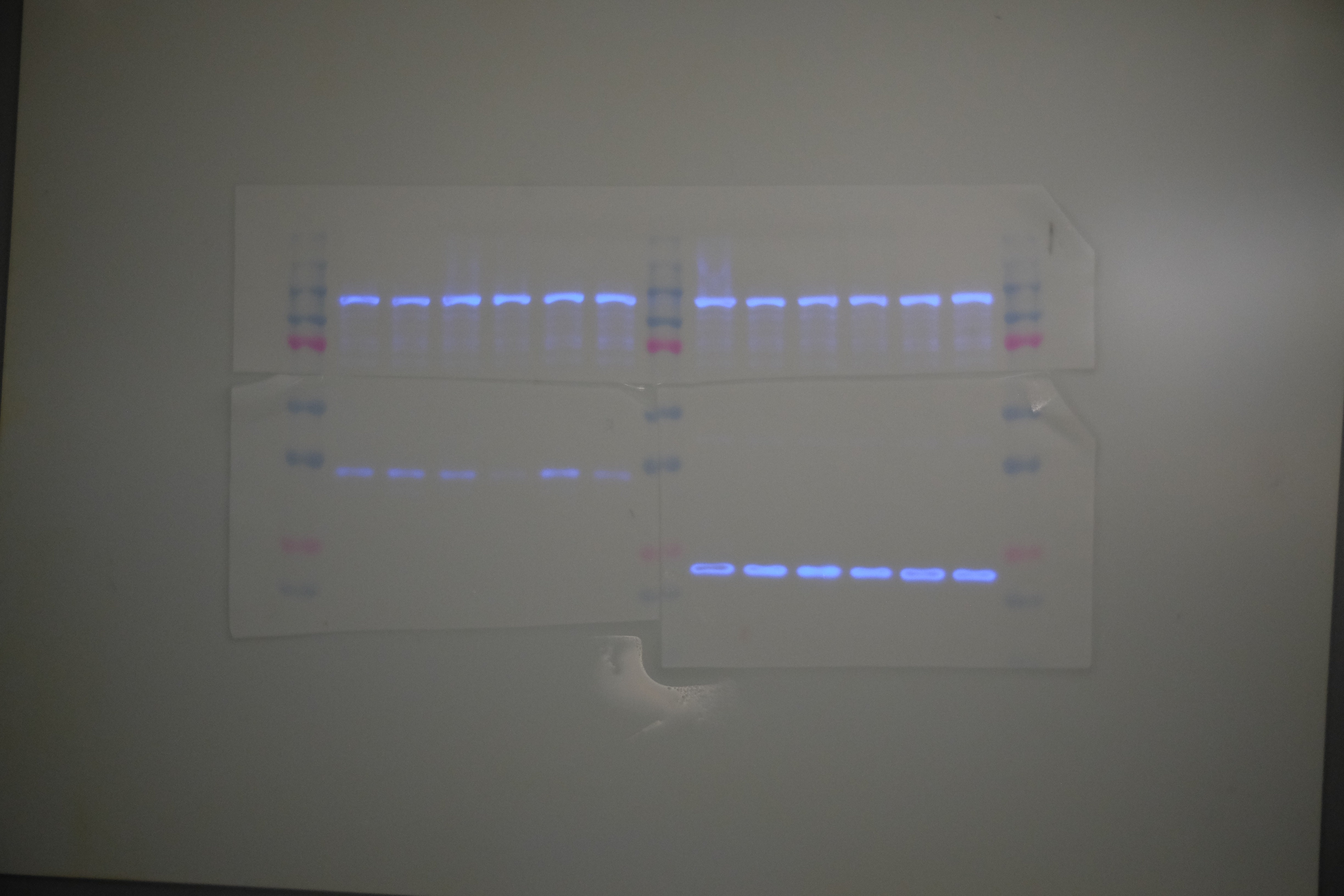

Supplement: Figure 6—figure supplement 1—source data 1. [file elife-78163-fig6-figsupp1-data1.zip › Figure 6-figure supplement 1-source data 1/DSCF3851.JPG]

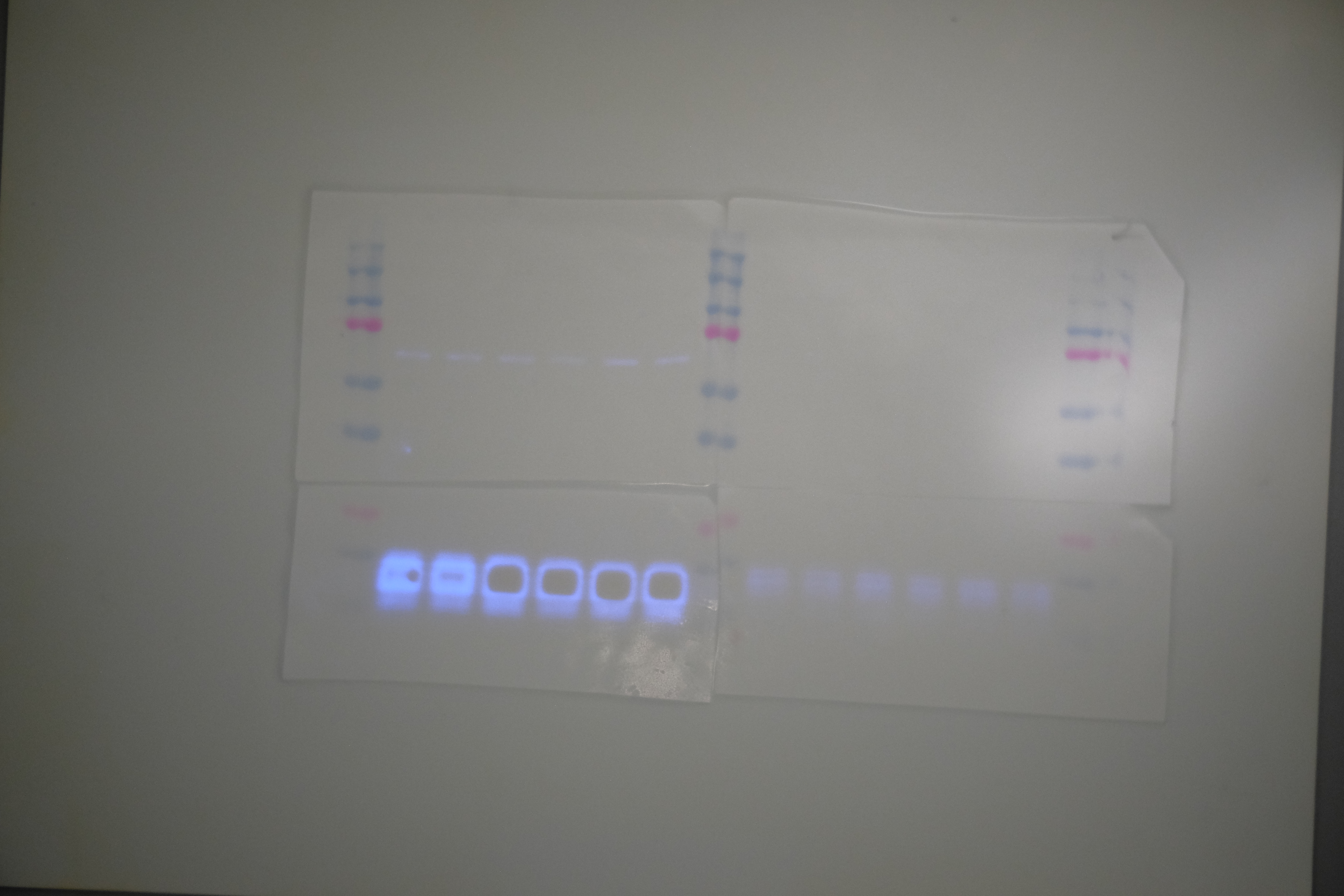

Supplement: Figure 6—figure supplement 1—source data 1. [file elife-78163-fig6-figsupp1-data1.zip › Figure 6-figure supplement 1-source data 1/DSCF3856.JPG]

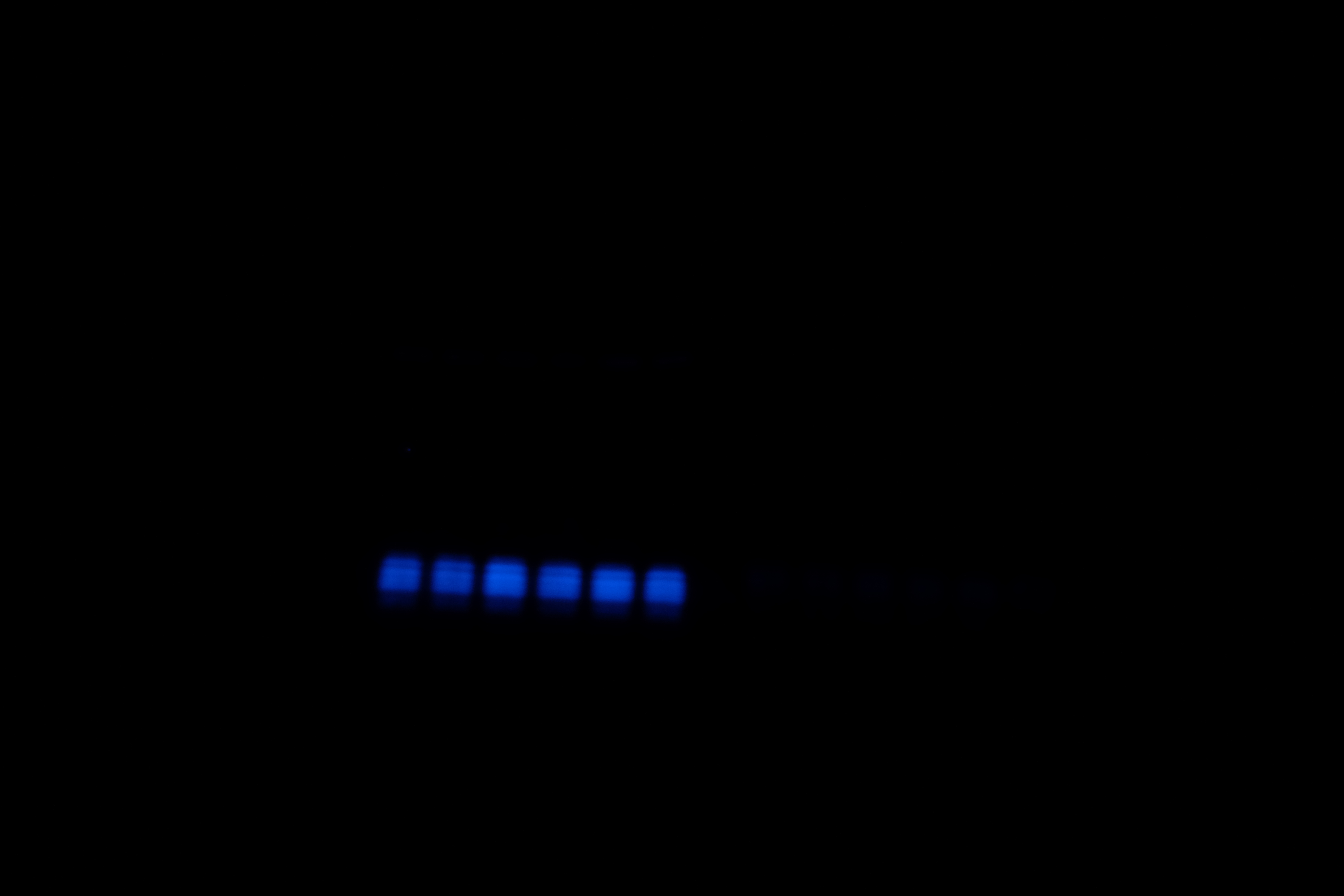

Supplement: Figure 6—figure supplement 1—source data 1. [file elife-78163-fig6-figsupp1-data1.zip › Figure 6-figure supplement 1-source data 1/Fig.6-S1E_4E-BP1.JPG]

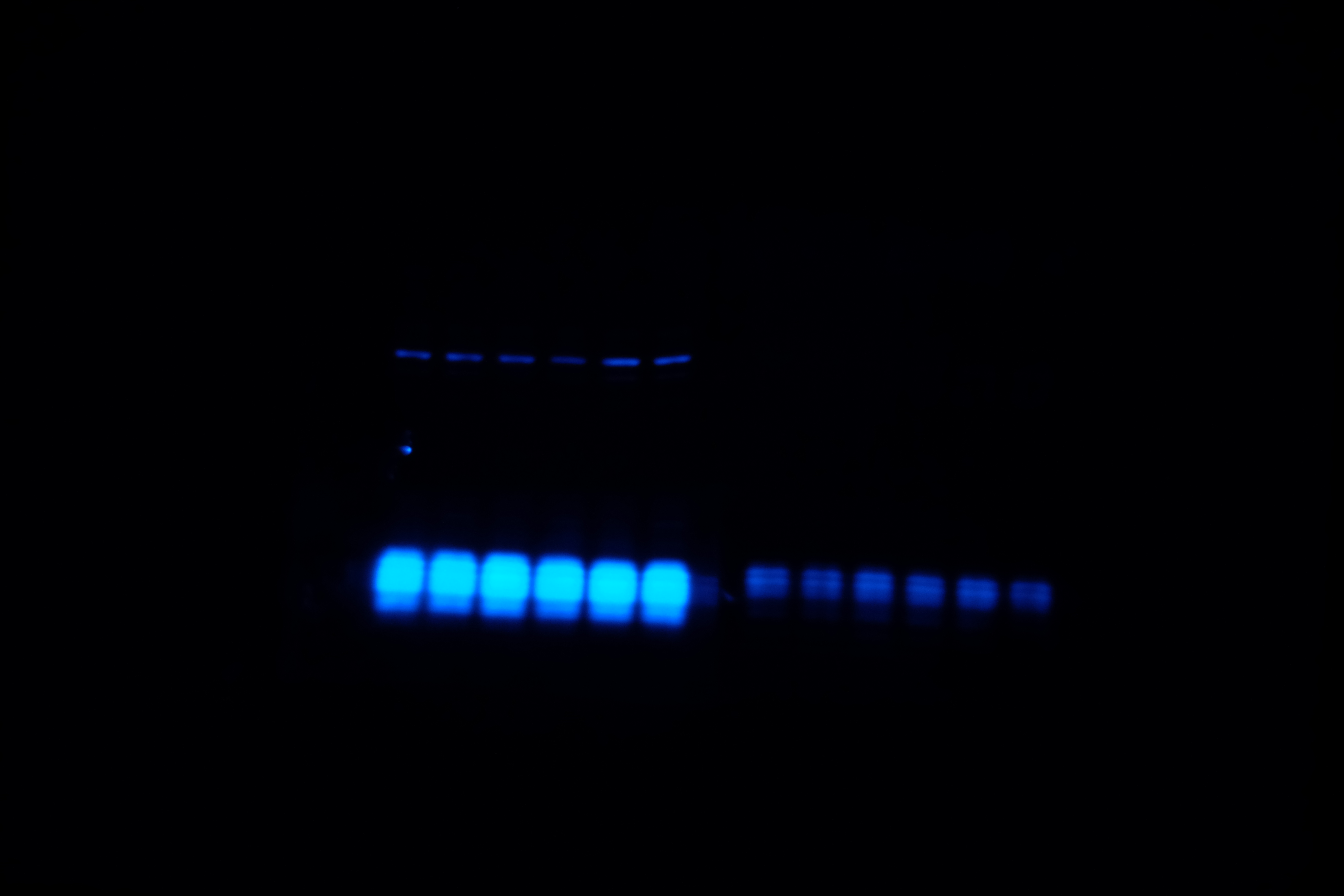

Supplement: Figure 6—figure supplement 1—source data 1. [file elife-78163-fig6-figsupp1-data1.zip › Figure 6-figure supplement 1-source data 1/Fig.6-S1E_p-4E-BP1.JPG]

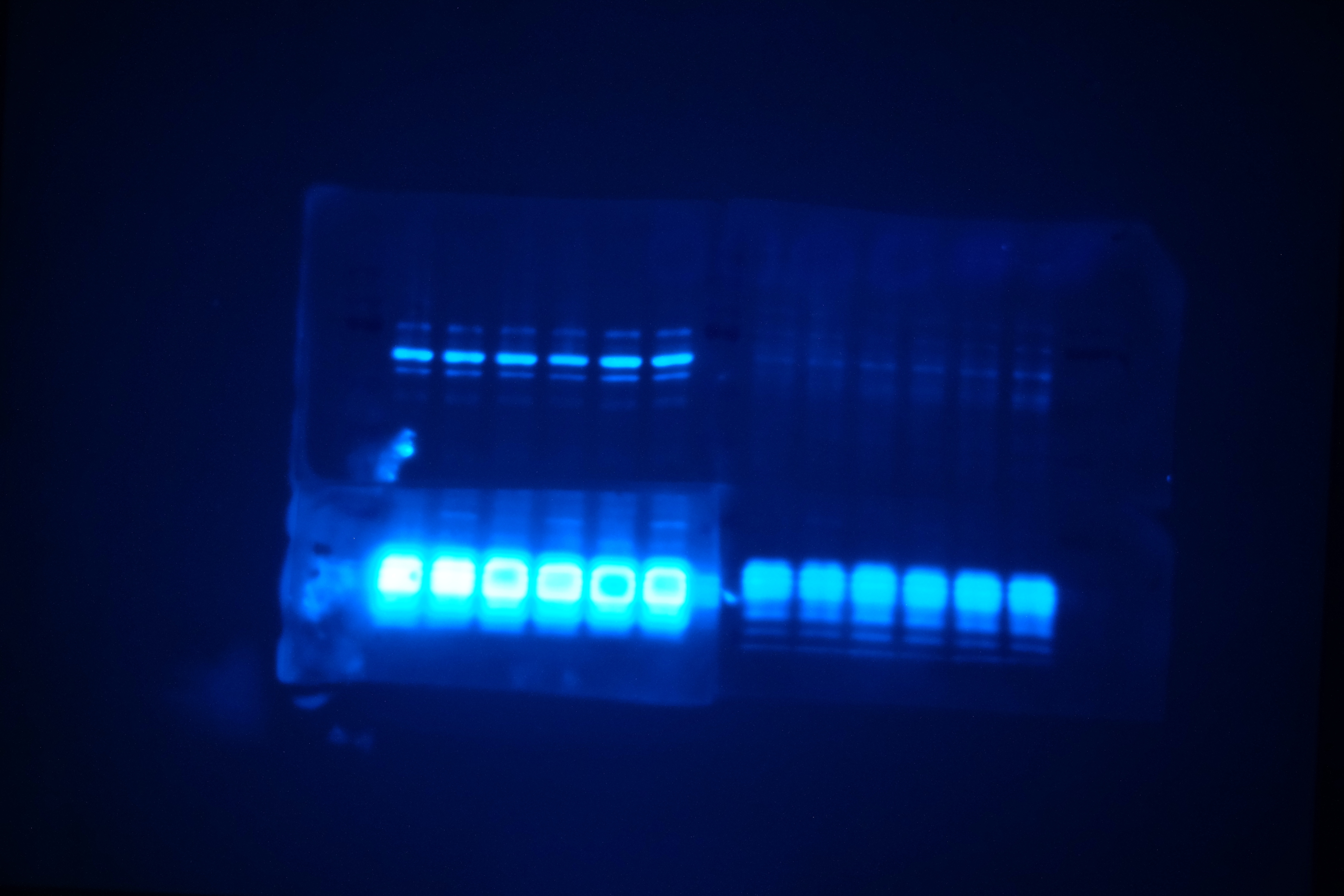

Supplement: Figure 6—figure supplement 1—source data 1. [file elife-78163-fig6-figsupp1-data1.zip › Figure 6-figure supplement 1-source data 1/Fig.6-S1E_p-S6K.JPG]

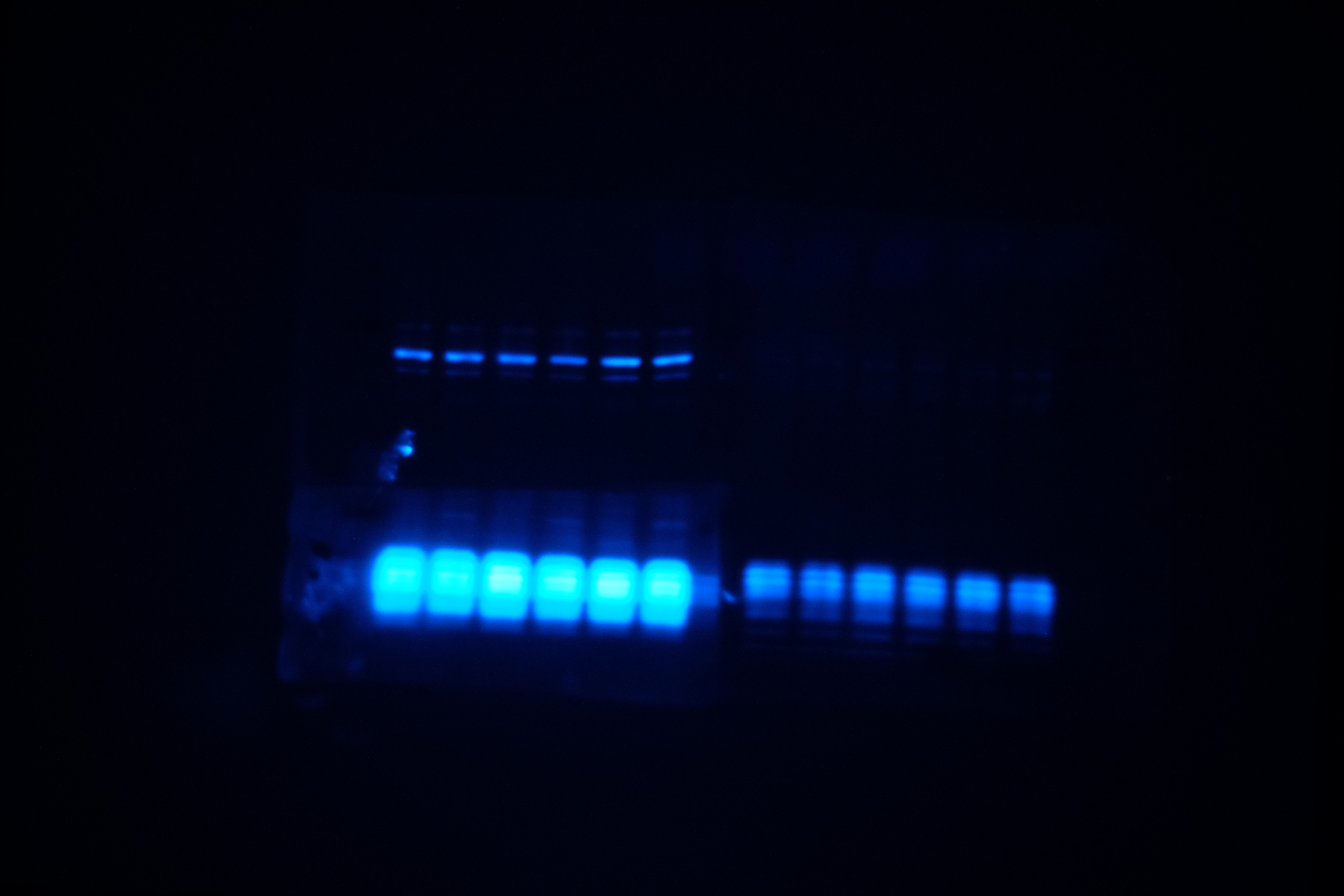

Supplement: Figure 6—figure supplement 1—source data 1. [file elife-78163-fig6-figsupp1-data1.zip › Figure 6-figure supplement 1-source data 1/Fig.6-S1E_S6K.JPG]

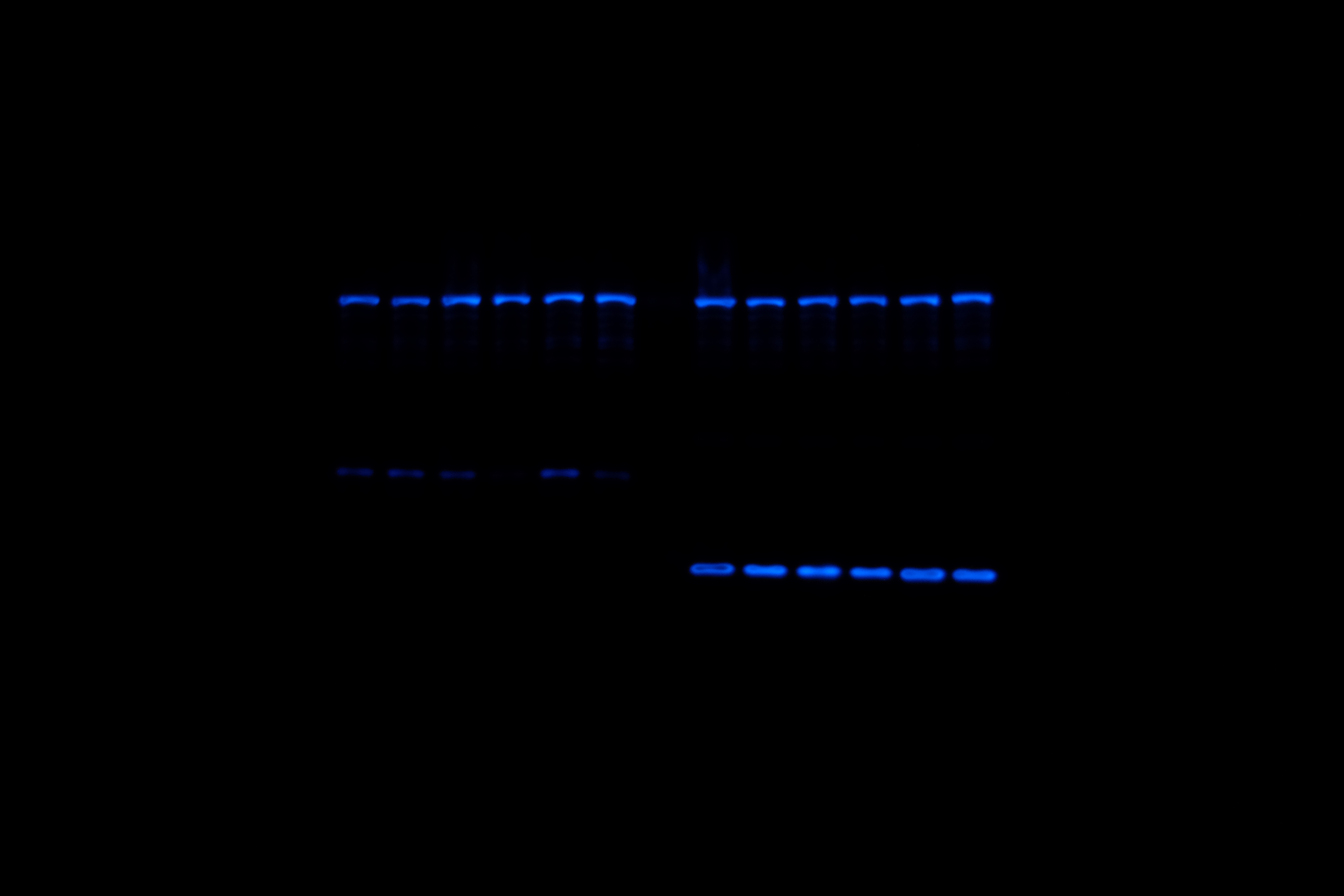

Supplement: Figure 6—figure supplement 1—source data 1. [file elife-78163-fig6-figsupp1-data1.zip › Figure 6-figure supplement 1-source data 1/Fig.6-S1E_vinculin.JPG]

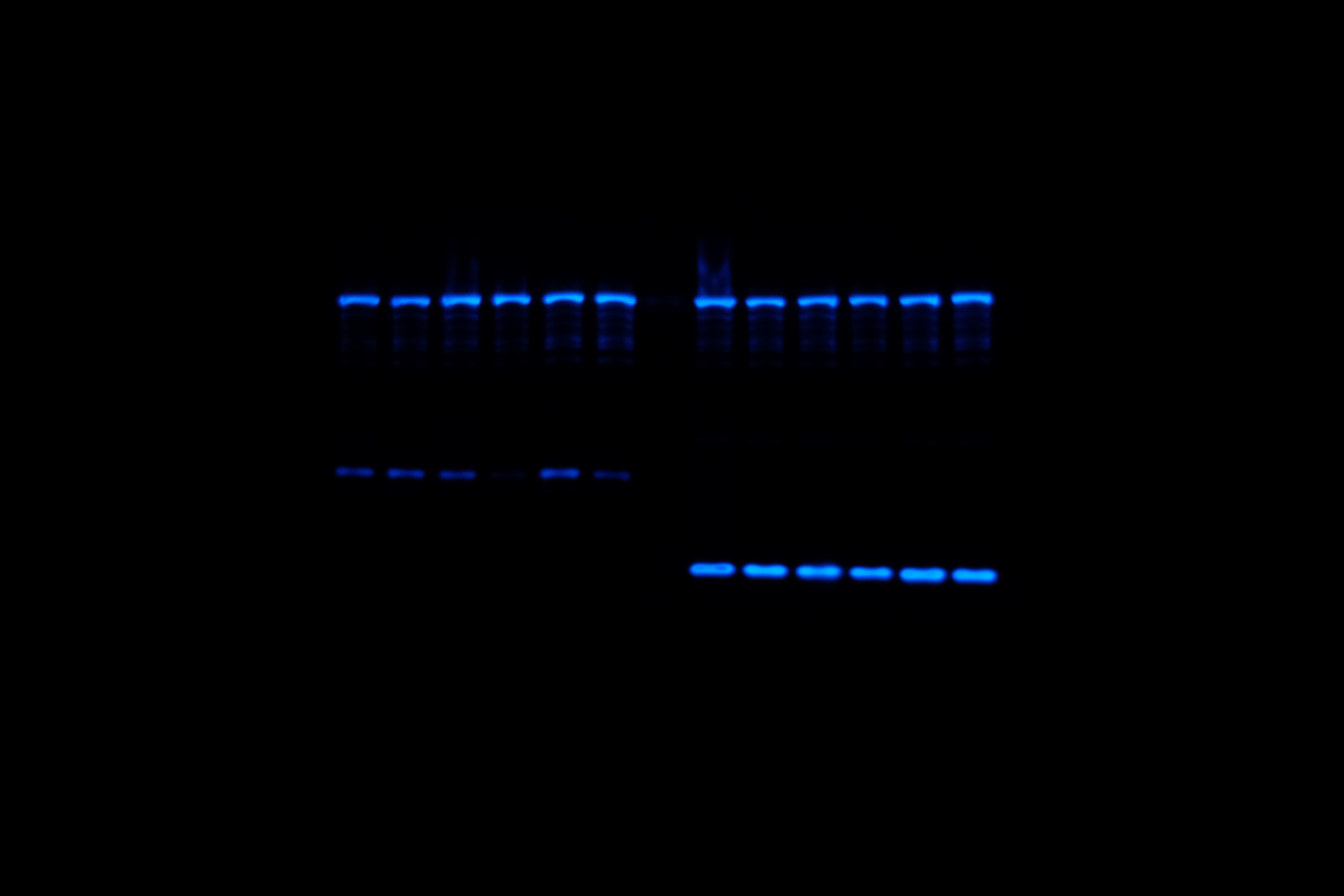

Supplement: Figure 6—figure supplement 1—source data 1. [file elife-78163-fig6-figsupp1-data1.zip › Figure 6-figure supplement 1-source data 1/Fig.6-S1E_WDR5.JPG]

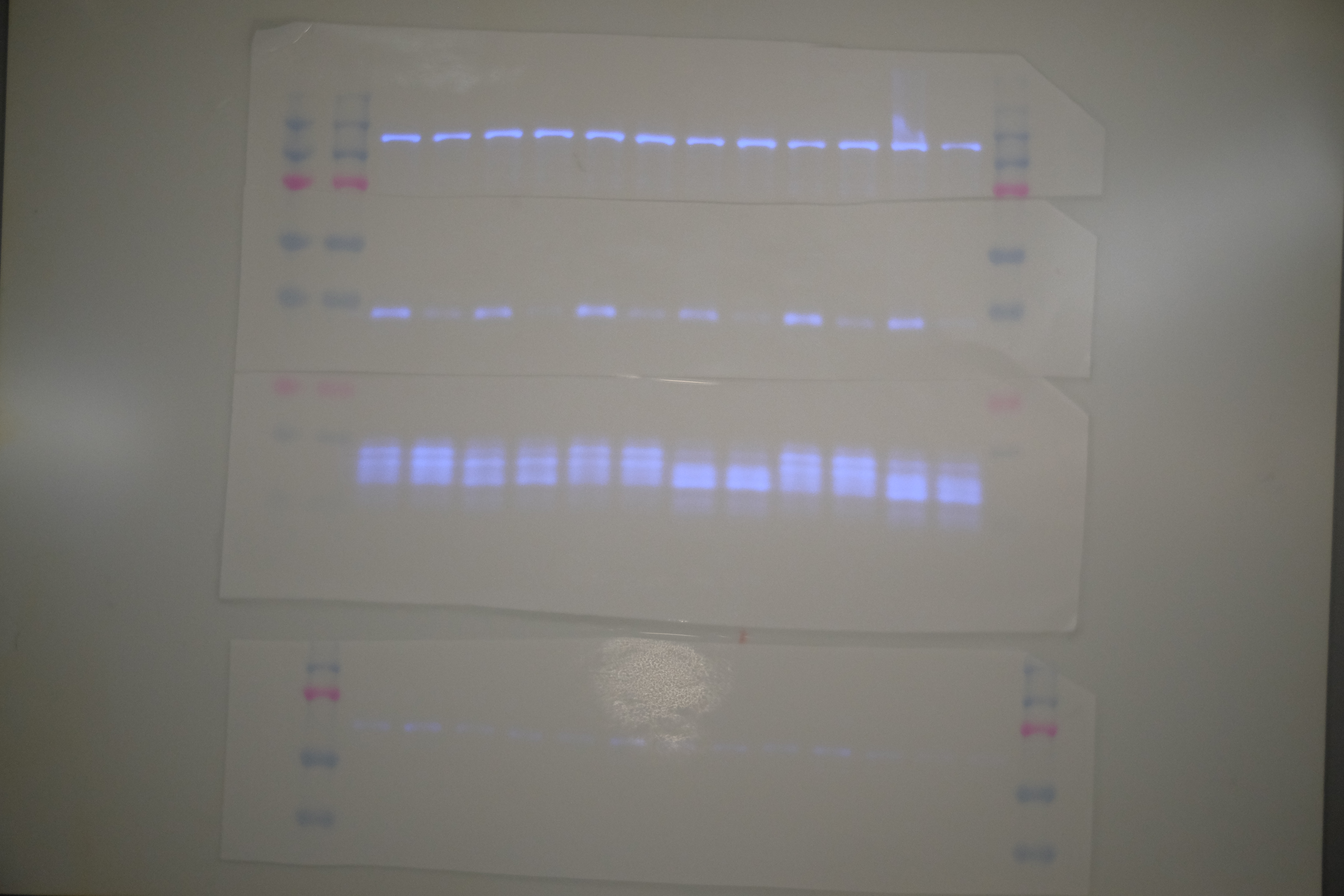

Supplement: Figure 6—figure supplement 1—source data 2. [file elife-78163-fig6-figsupp1-data2.zip › Figure 6-figure supplement 1-source data 2/DSCF3725.JPG]

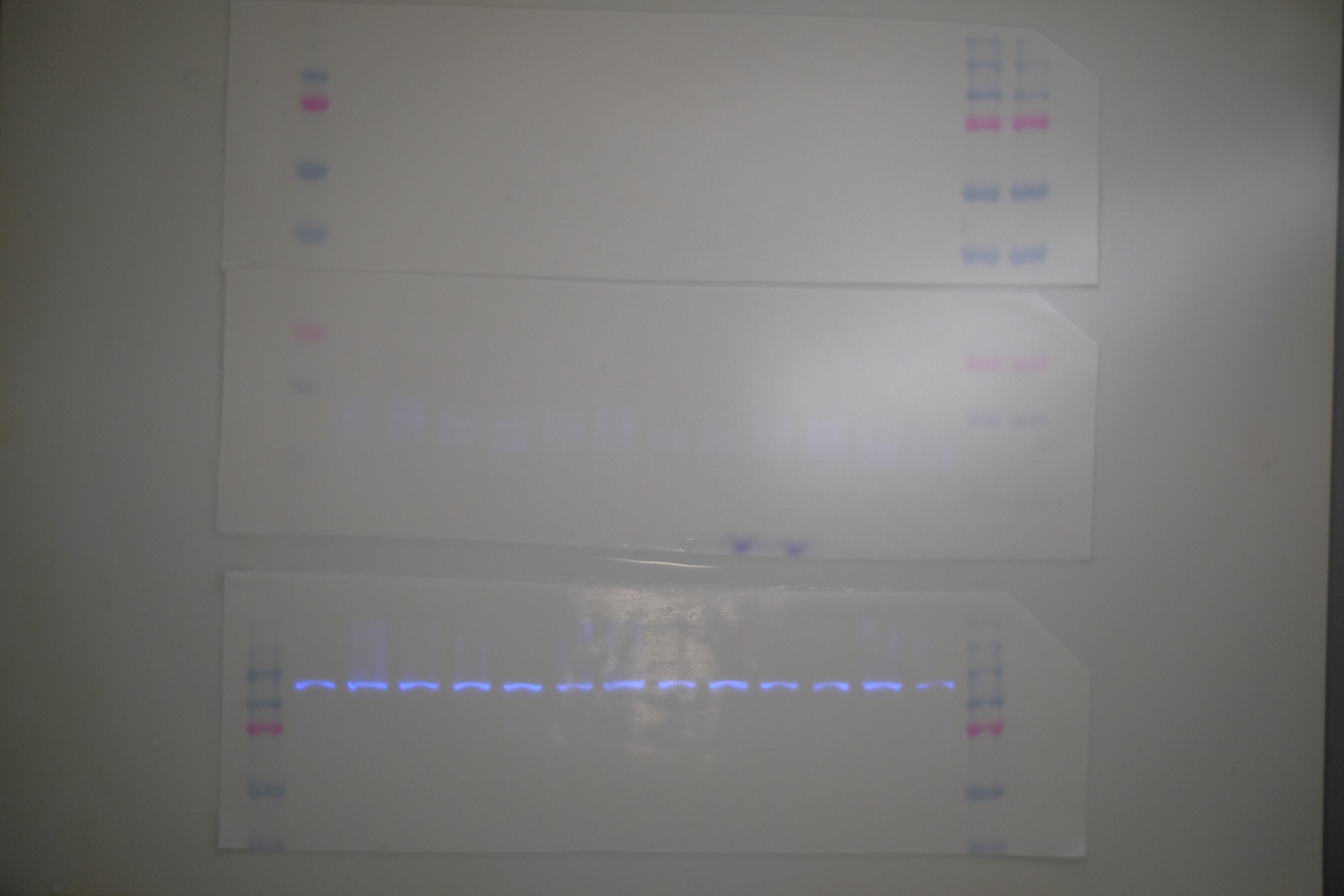

Supplement: Figure 6—figure supplement 1—source data 2. [file elife-78163-fig6-figsupp1-data2.zip › Figure 6-figure supplement 1-source data 2/DSCF3733.JPG]

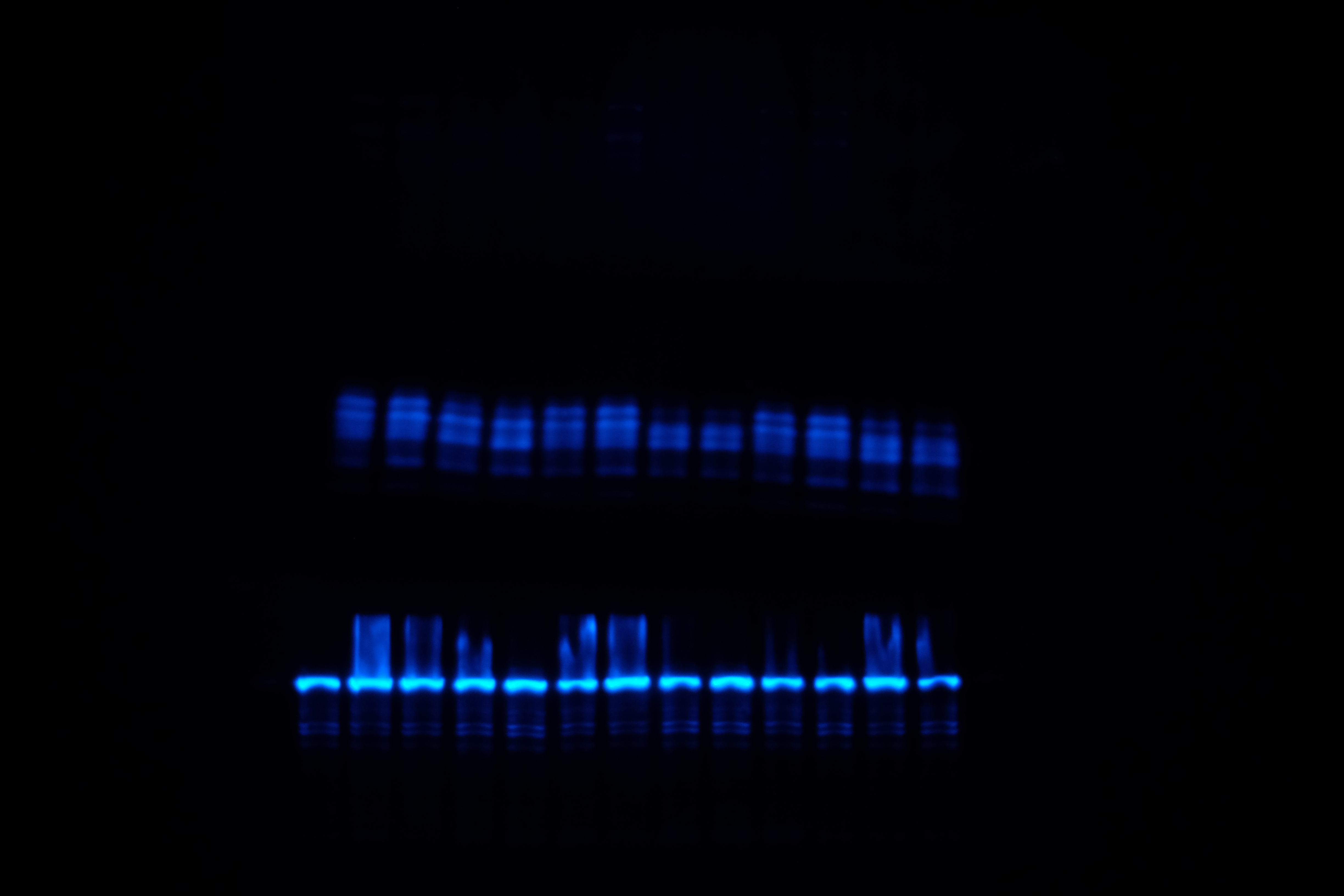

Supplement: Figure 6—figure supplement 1—source data 2. [file elife-78163-fig6-figsupp1-data2.zip › Figure 6-figure supplement 1-source data 2/Fig.6-S1F_p-4E-BP1.JPG]

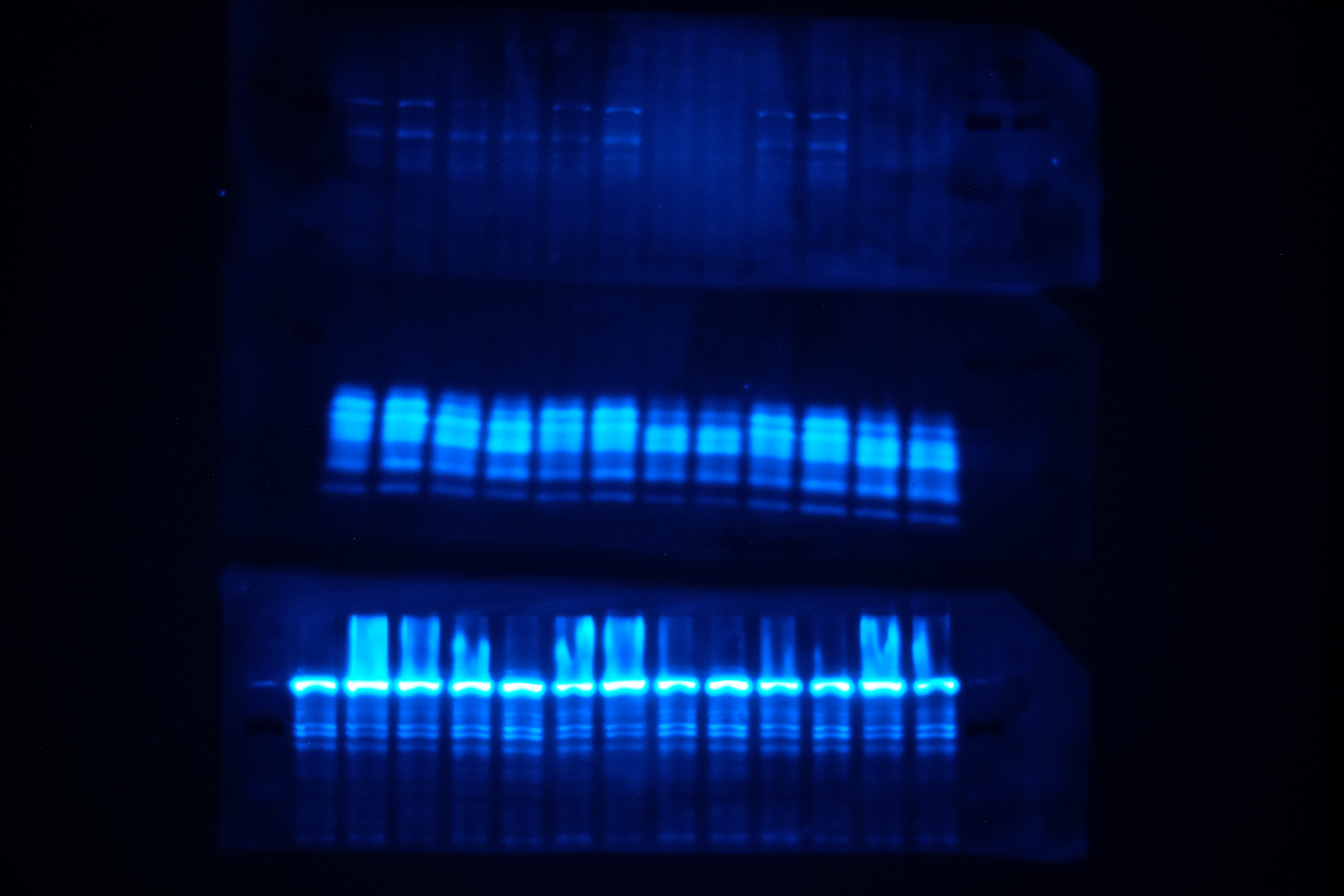

Supplement: Figure 6—figure supplement 1—source data 2. [file elife-78163-fig6-figsupp1-data2.zip › Figure 6-figure supplement 1-source data 2/Fig.6-S1F_p-S6K.JPG]

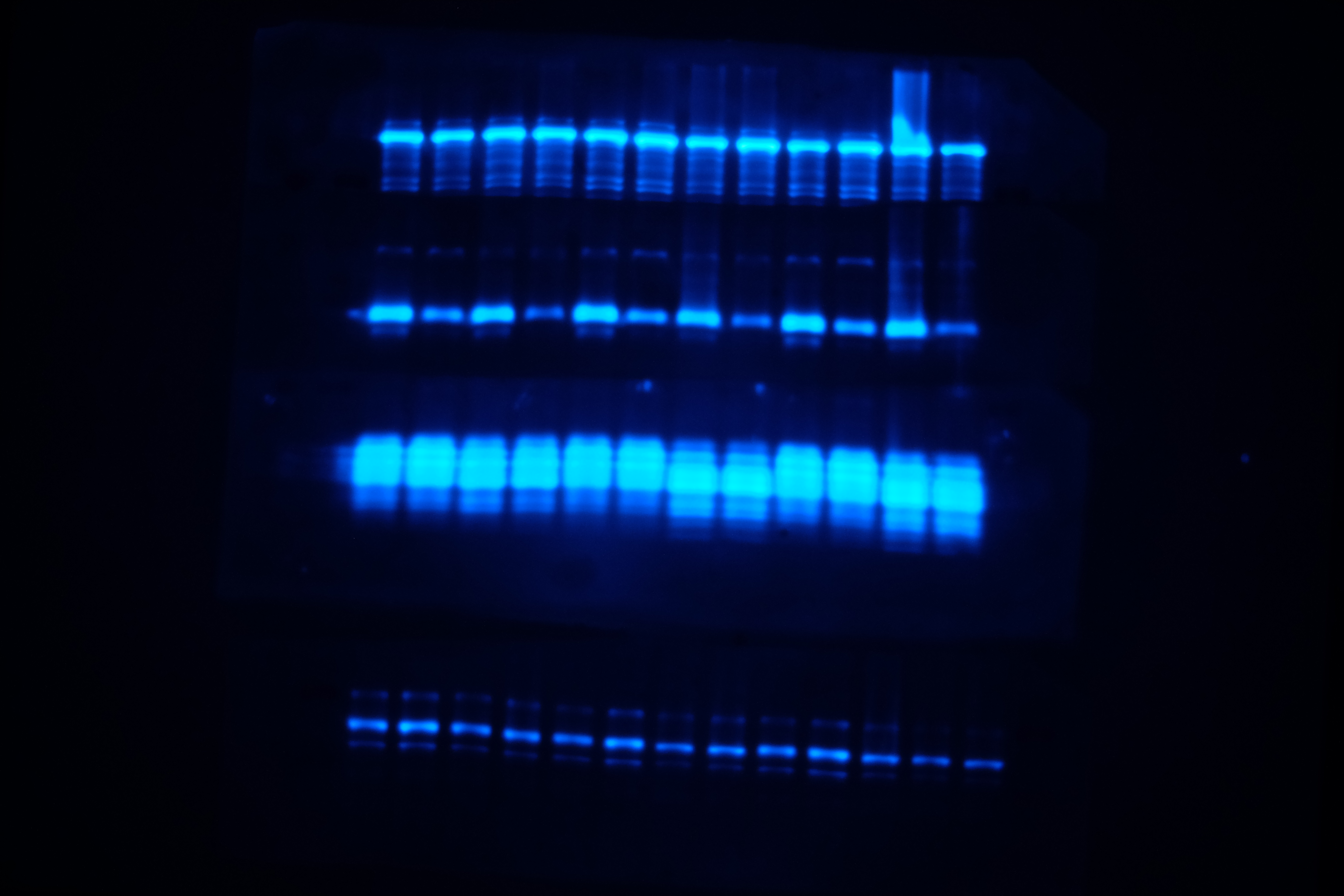

Supplement: Figure 6—figure supplement 1—source data 2. [file elife-78163-fig6-figsupp1-data2.zip › Figure 6-figure supplement 1-source data 2/Fig.6-S1F_S6K.JPG]

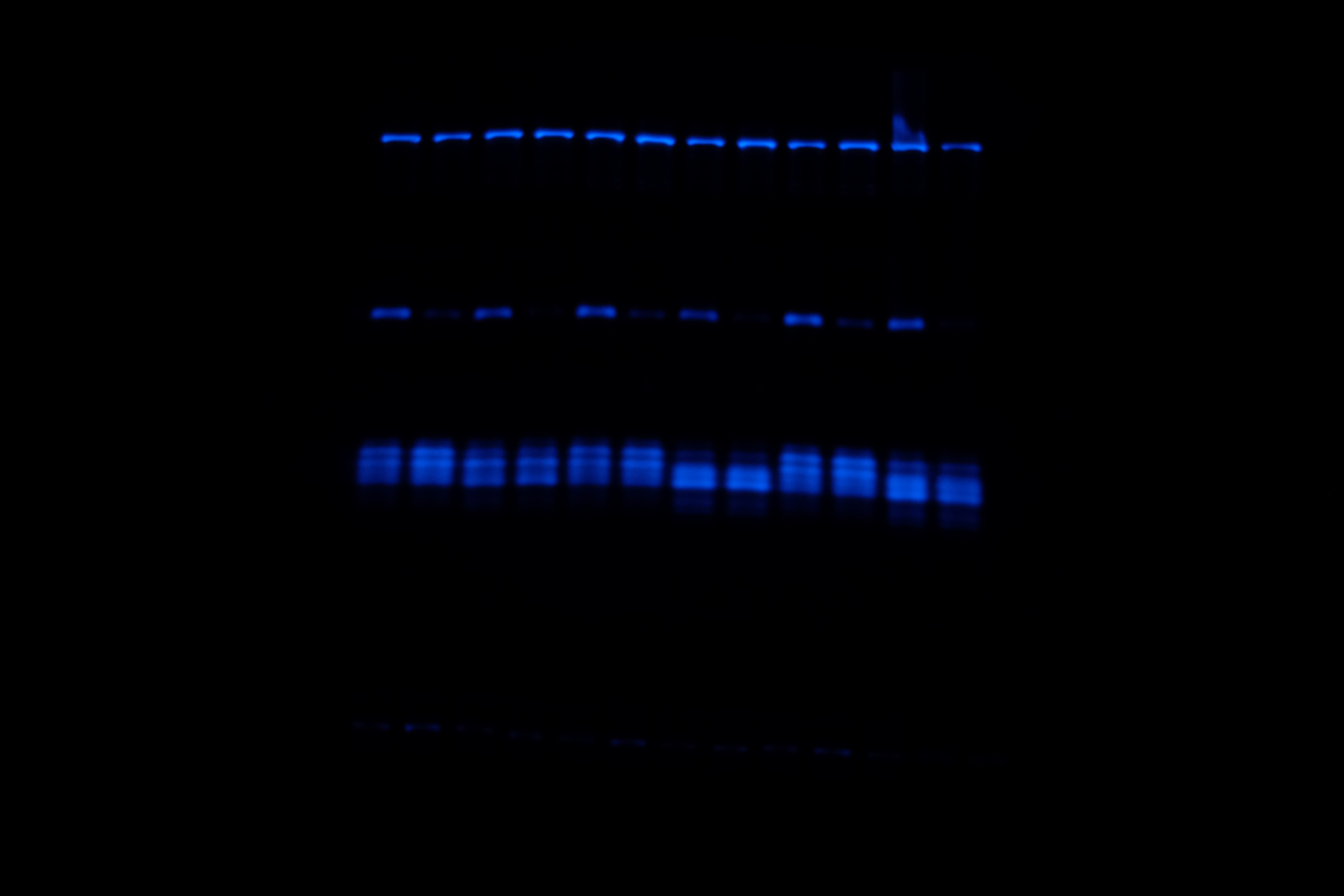

Supplement: Figure 6—figure supplement 1—source data 2. [file elife-78163-fig6-figsupp1-data2.zip › Figure 6-figure supplement 1-source data 2/Fig.6-S1F_vinculin_4E-BP1.JPG]

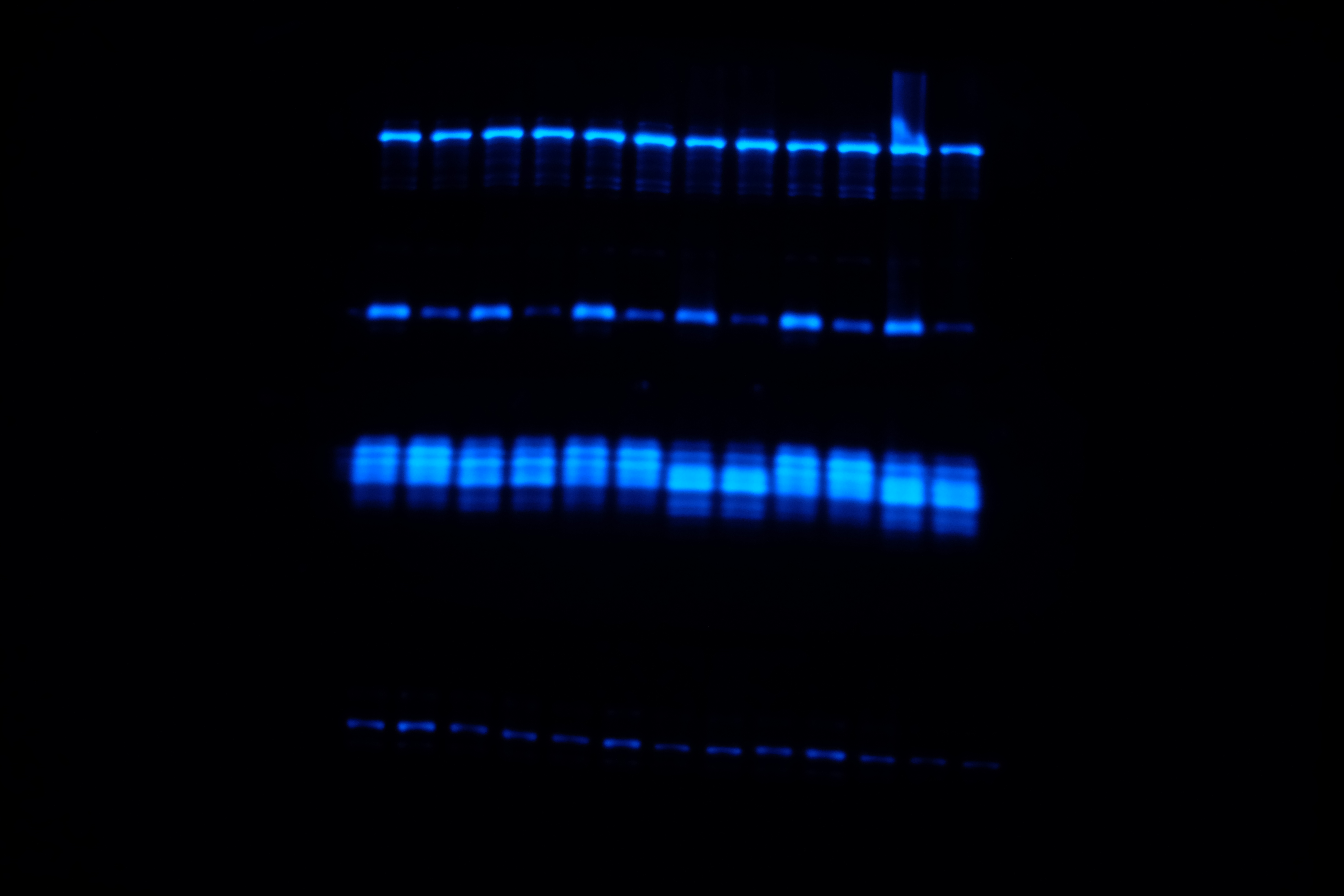

Supplement: Figure 6—figure supplement 1—source data 2. [file elife-78163-fig6-figsupp1-data2.zip › Figure 6-figure supplement 1-source data 2/Fig.6-S1F_WDR5.JPG]

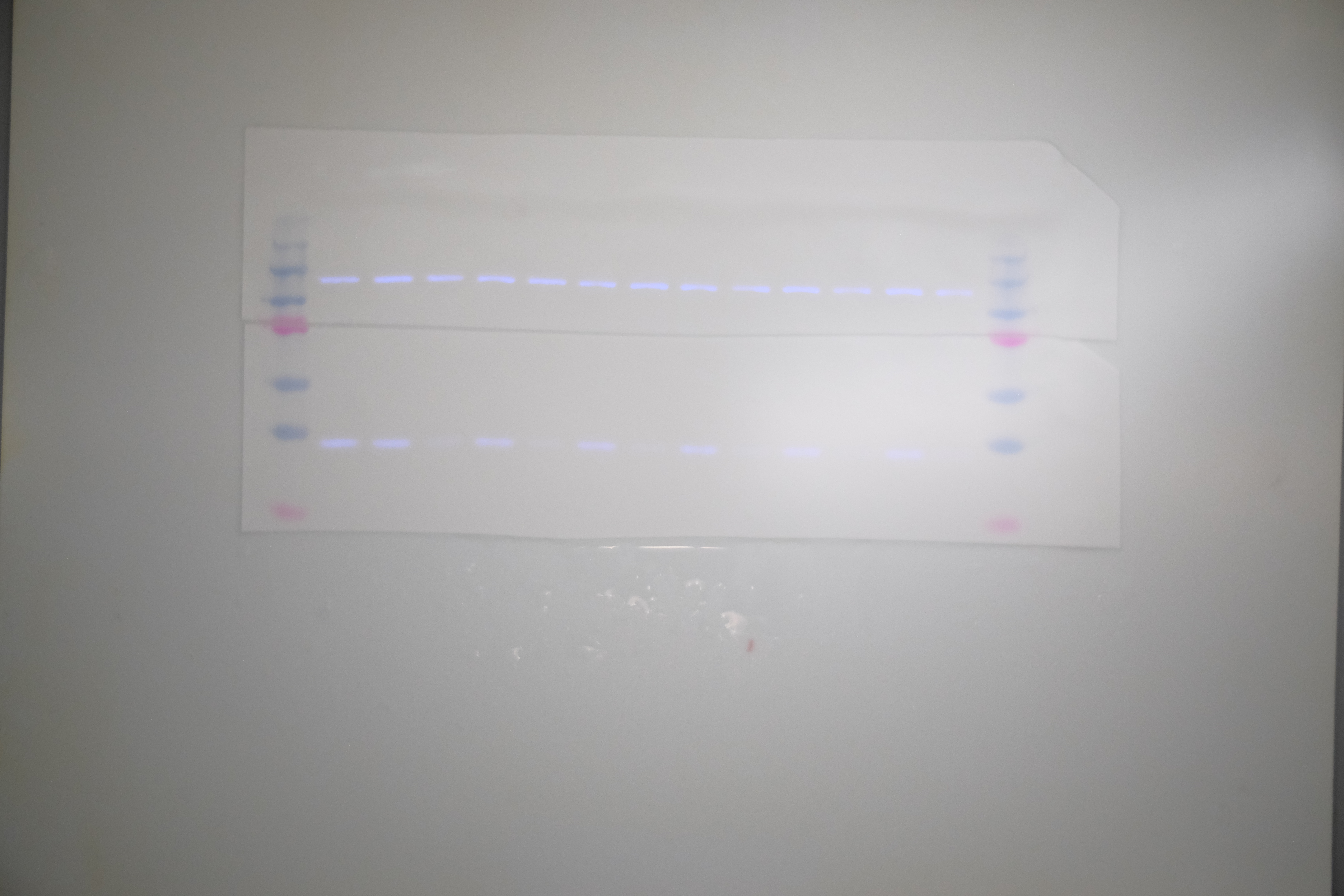

Supplement: Figure 7—source data 1. [file elife-78163-fig7-data1.zip › Figure 7-source data 1/DSCF4233.JPG]

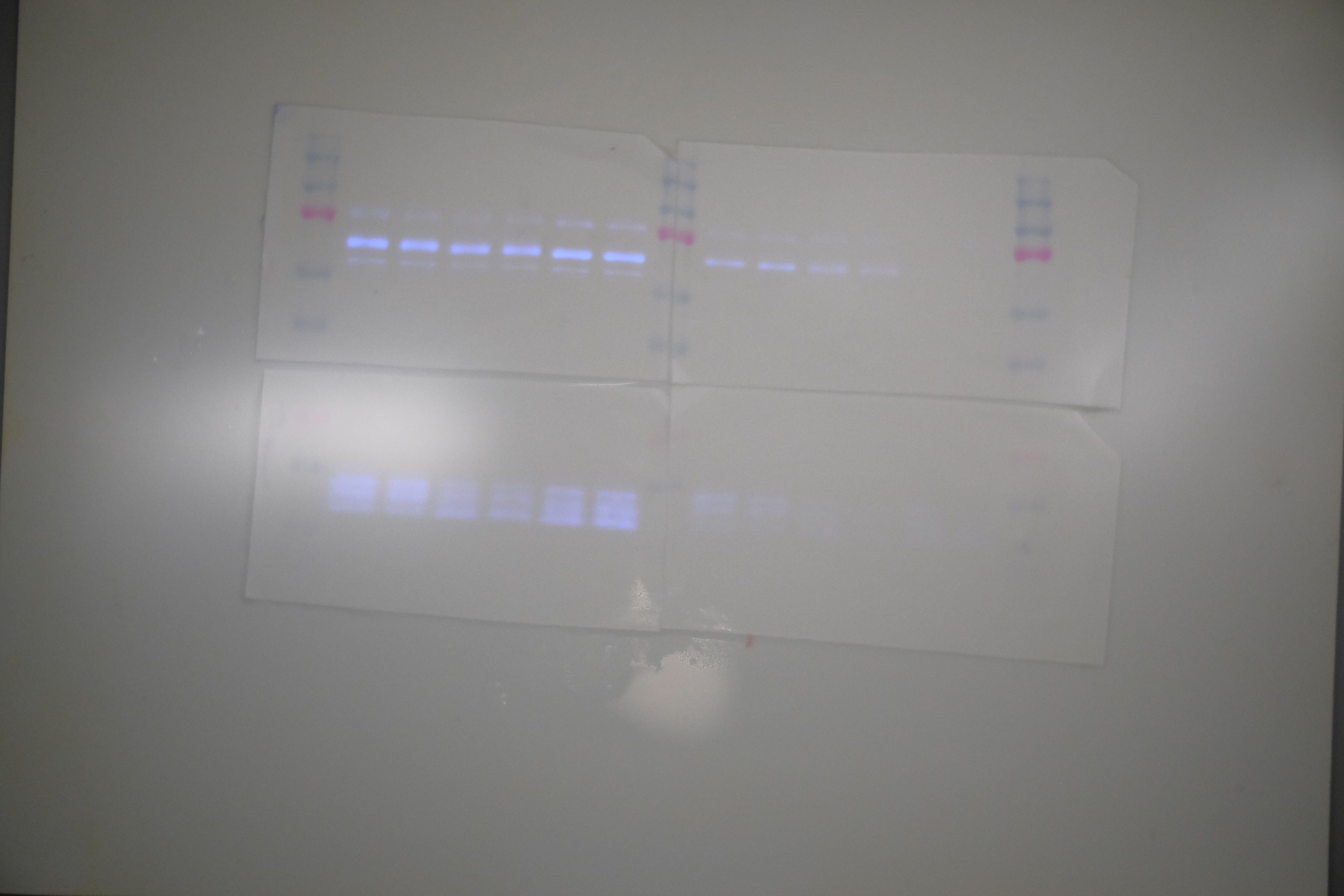

Supplement: Figure 7—source data 1. [file elife-78163-fig7-data1.zip › Figure 7-source data 1/DSCF4236.JPG]

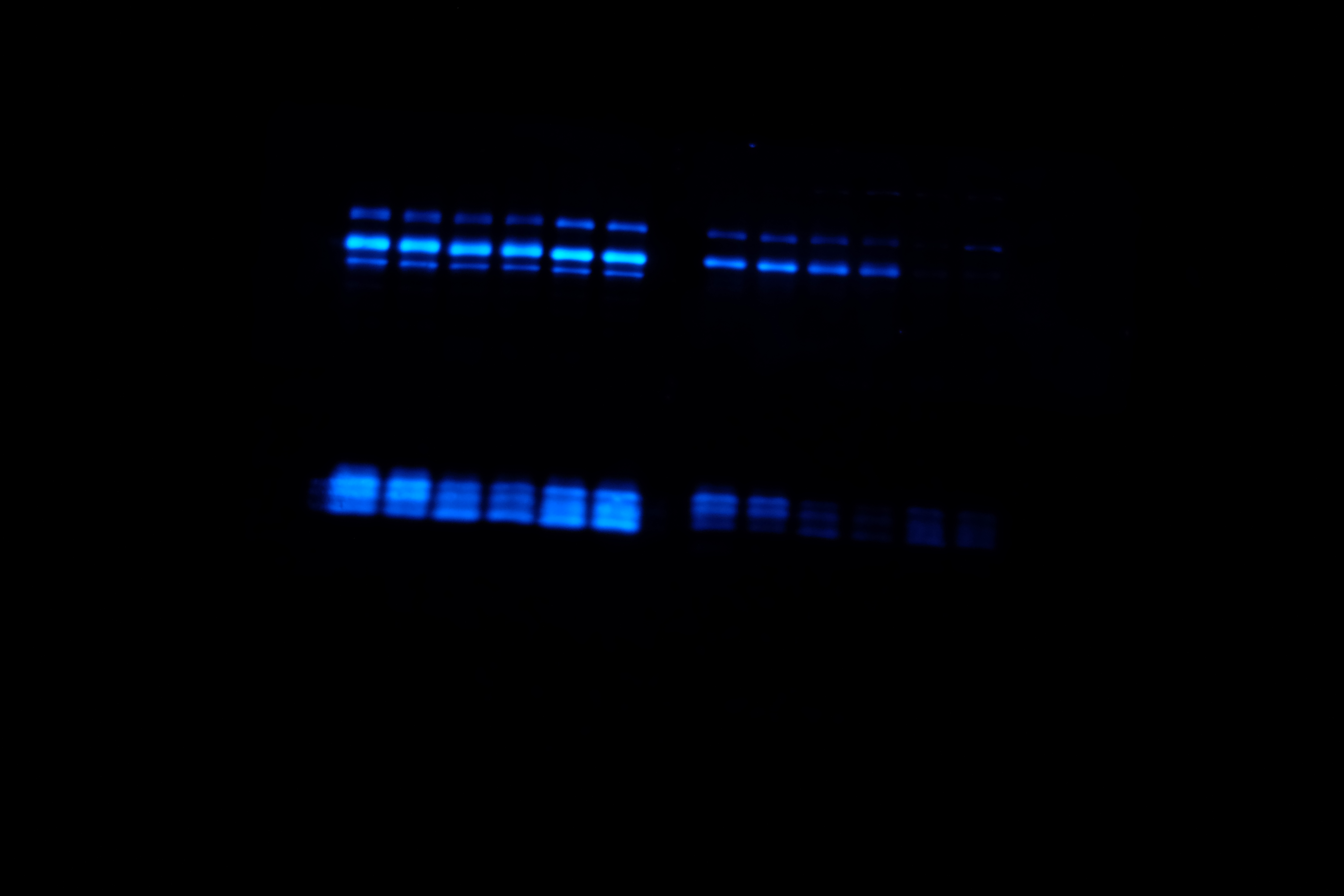

Supplement: Figure 7—source data 1. [file elife-78163-fig7-data1.zip › Figure 7-source data 1/Fig.7A_p-4E-BP1.JPG]

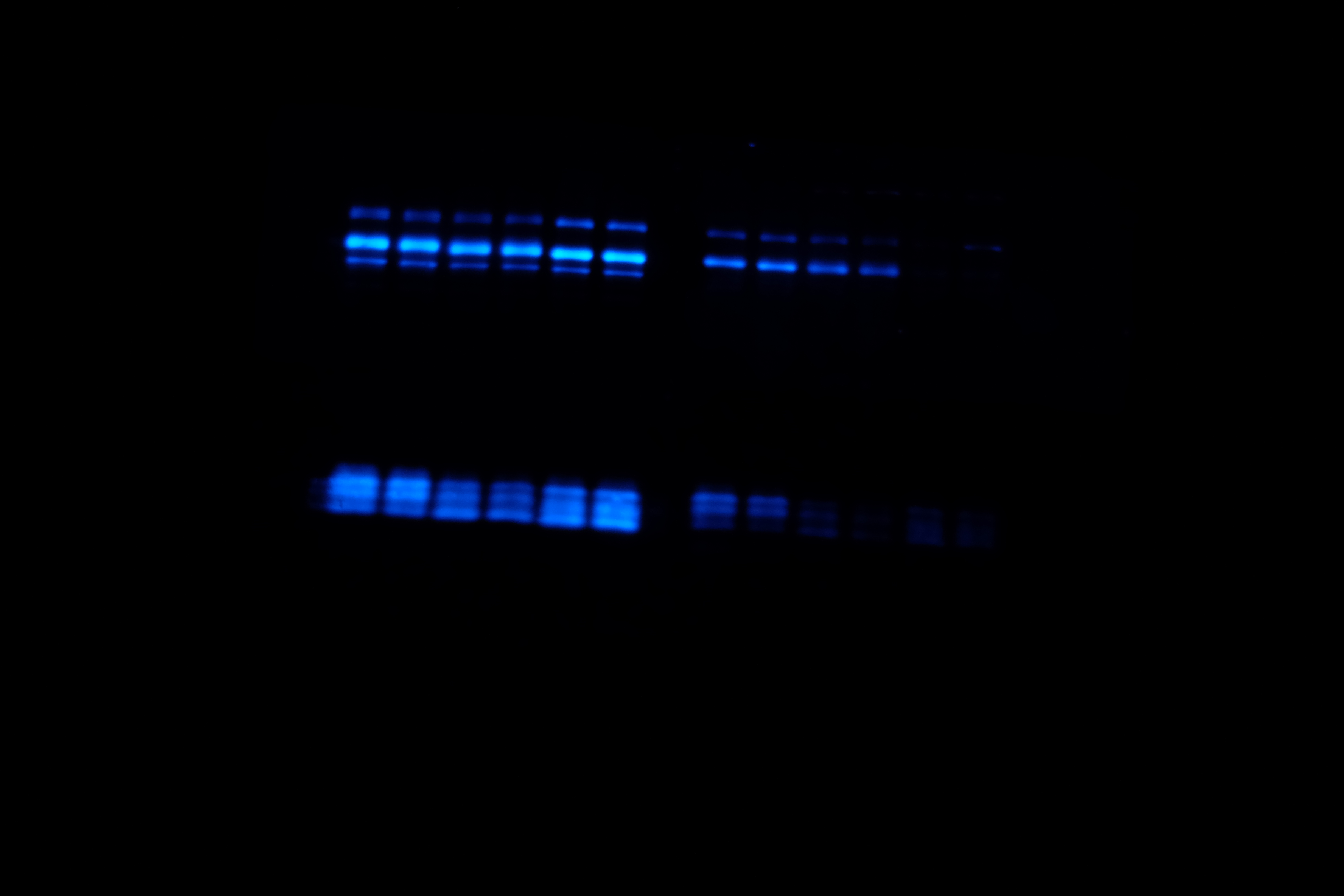

Supplement: Figure 7—source data 1. [file elife-78163-fig7-data1.zip › Figure 7-source data 1/Fig.7A_p-S6K_4E-BP1.JPG]

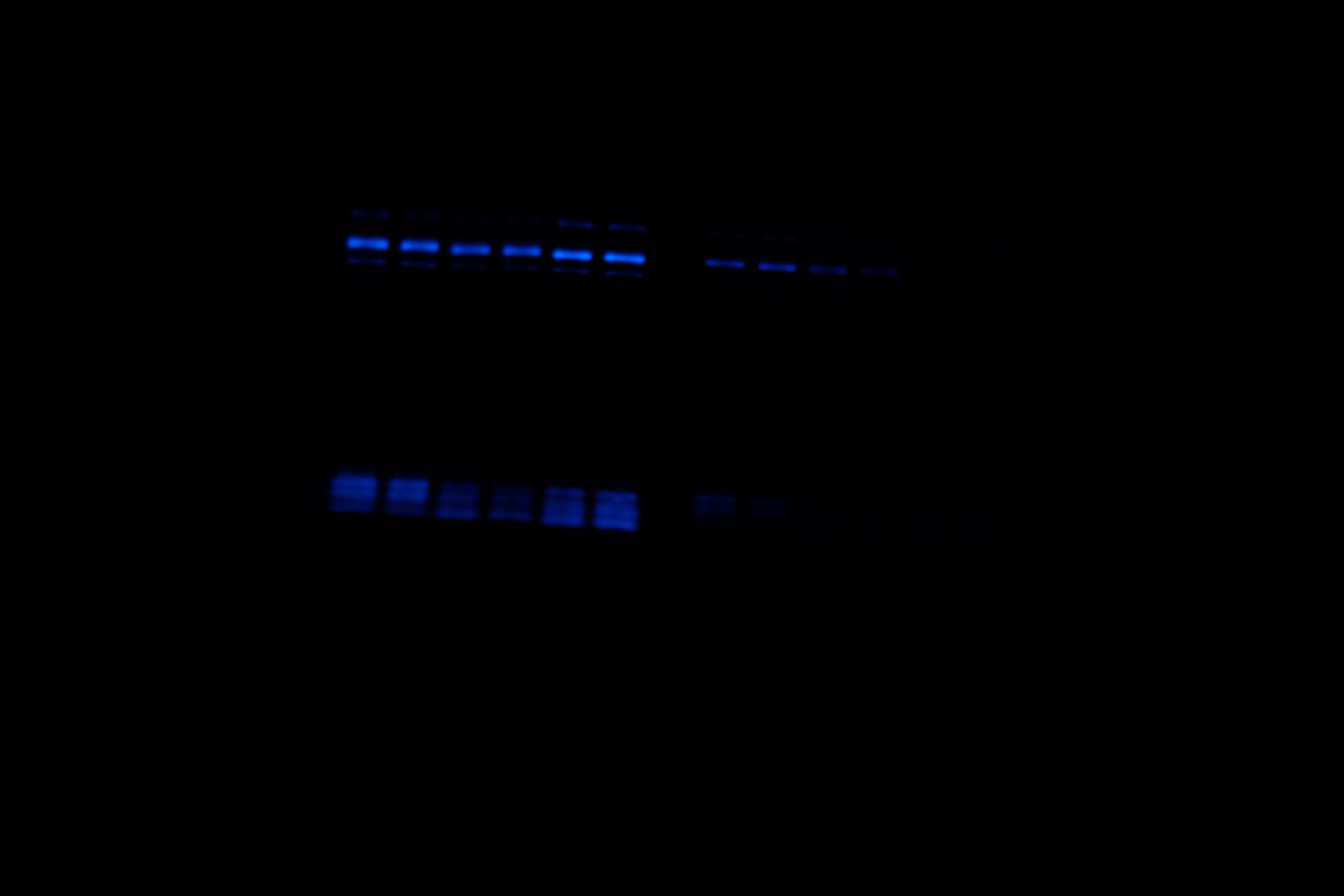

Supplement: Figure 7—source data 1. [file elife-78163-fig7-data1.zip › Figure 7-source data 1/Fig.7A_S6K.JPG]

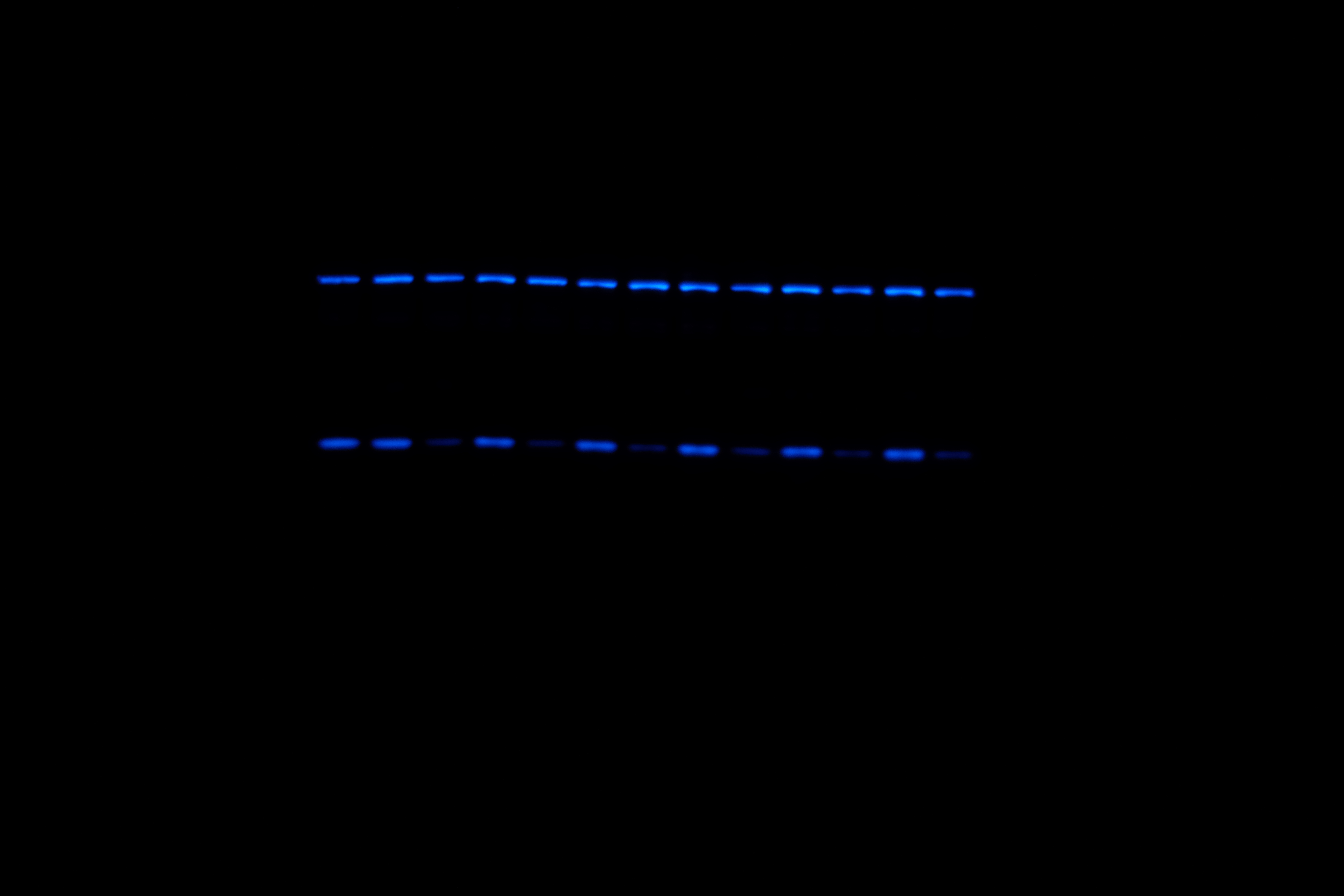

Supplement: Figure 7—source data 1. [file elife-78163-fig7-data1.zip › Figure 7-source data 1/Fig.7A_WDR5_vinculin.JPG]

## Slide 1
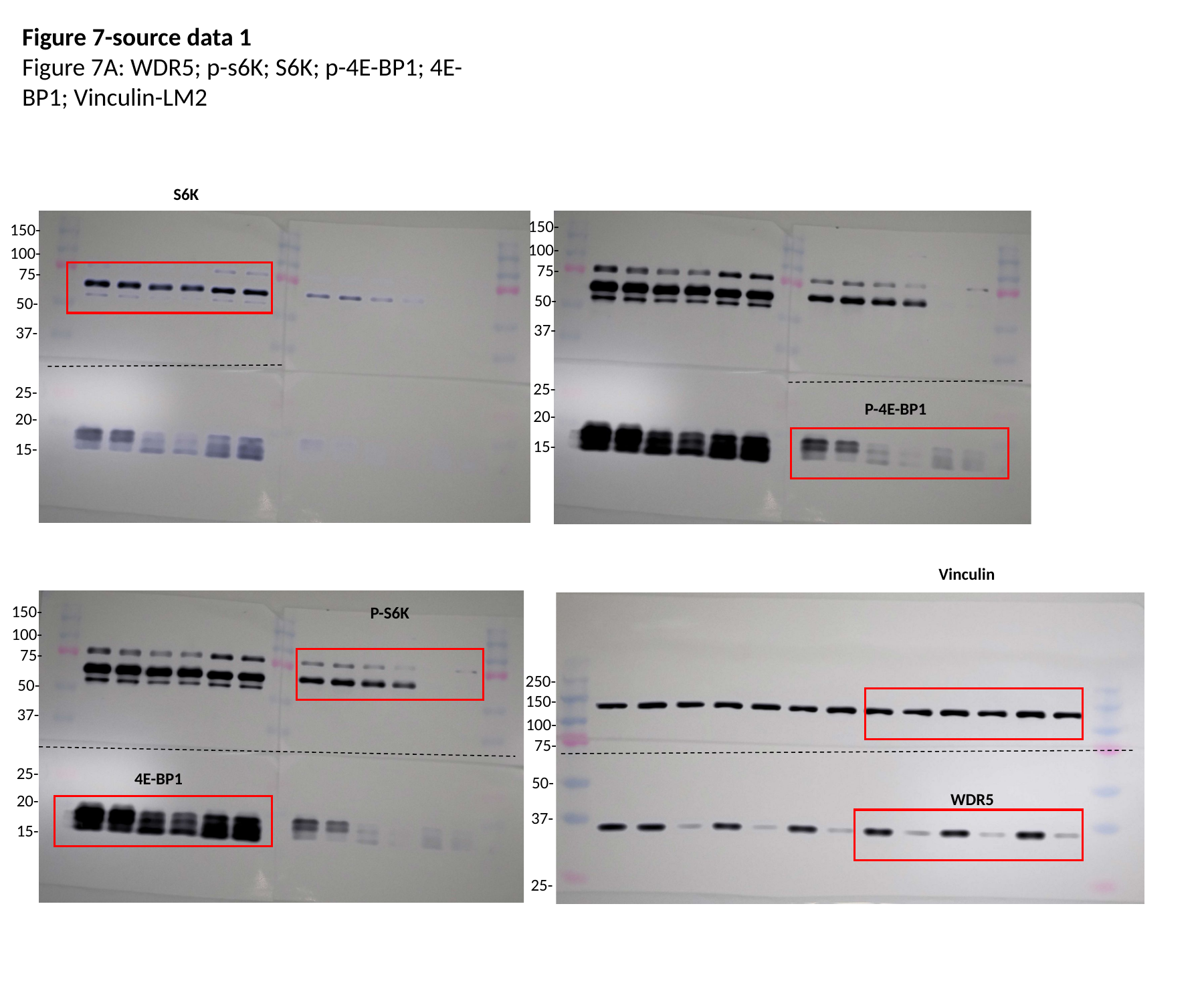

Figure 7-source data 1
Figure 7A: WDR5; p-s6K; S6K; p-4E-BP1; 4E-BP1; Vinculin-LM2
S6K
150-
150-
100-
100-
75-
75-
50-
50-
37-
37-
25-
25-
P-4E-BP1
20-
20-
15-
15-
Vinculin
150-
P-S6K
100-
75-
250-
50-
150-
37-
100-
75-
25-
4E-BP1
50-
WDR5
20-
37-
15-
25-

Supplement: Figure 7—source data 1. [file elife-78163-fig7-data1.zip › Figure 7-source data 1/Figure 7-source data 1_labeled images.pptx]

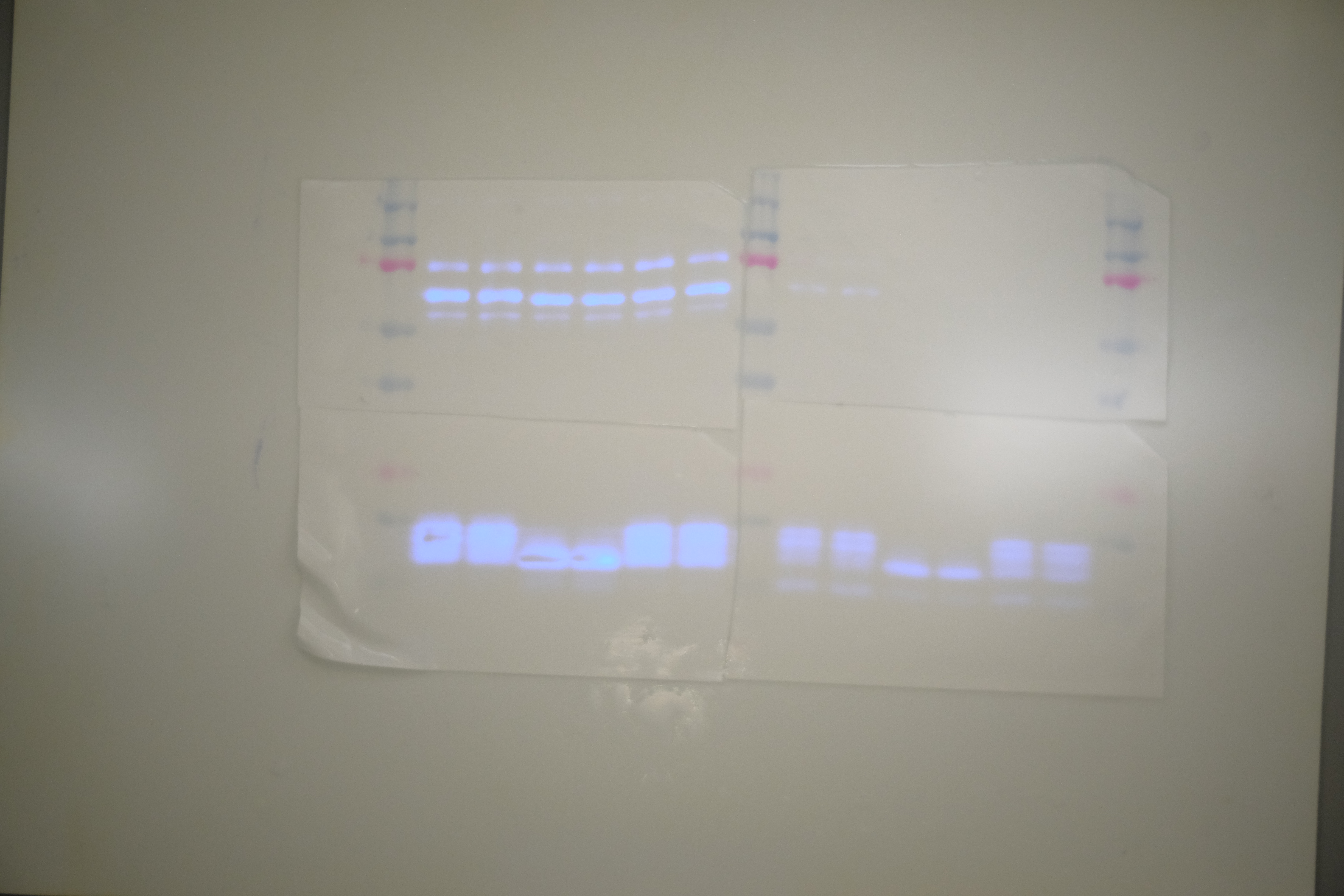

Supplement: Figure 7—source data 2. [file elife-78163-fig7-data2.zip › Figure 7-source data 2/DSCF4267.JPG]

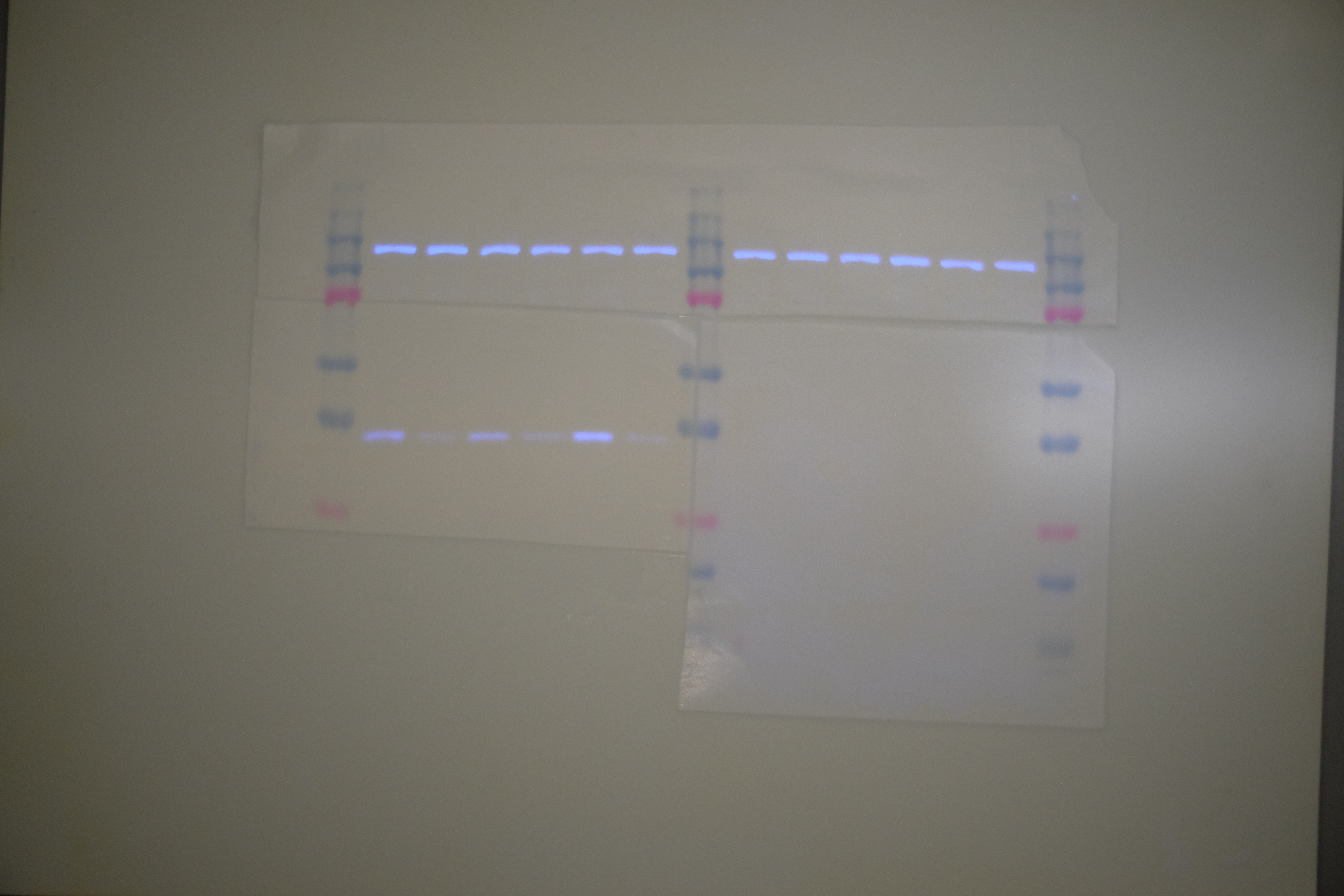

Supplement: Figure 7—source data 2. [file elife-78163-fig7-data2.zip › Figure 7-source data 2/DSCF4273.JPG]

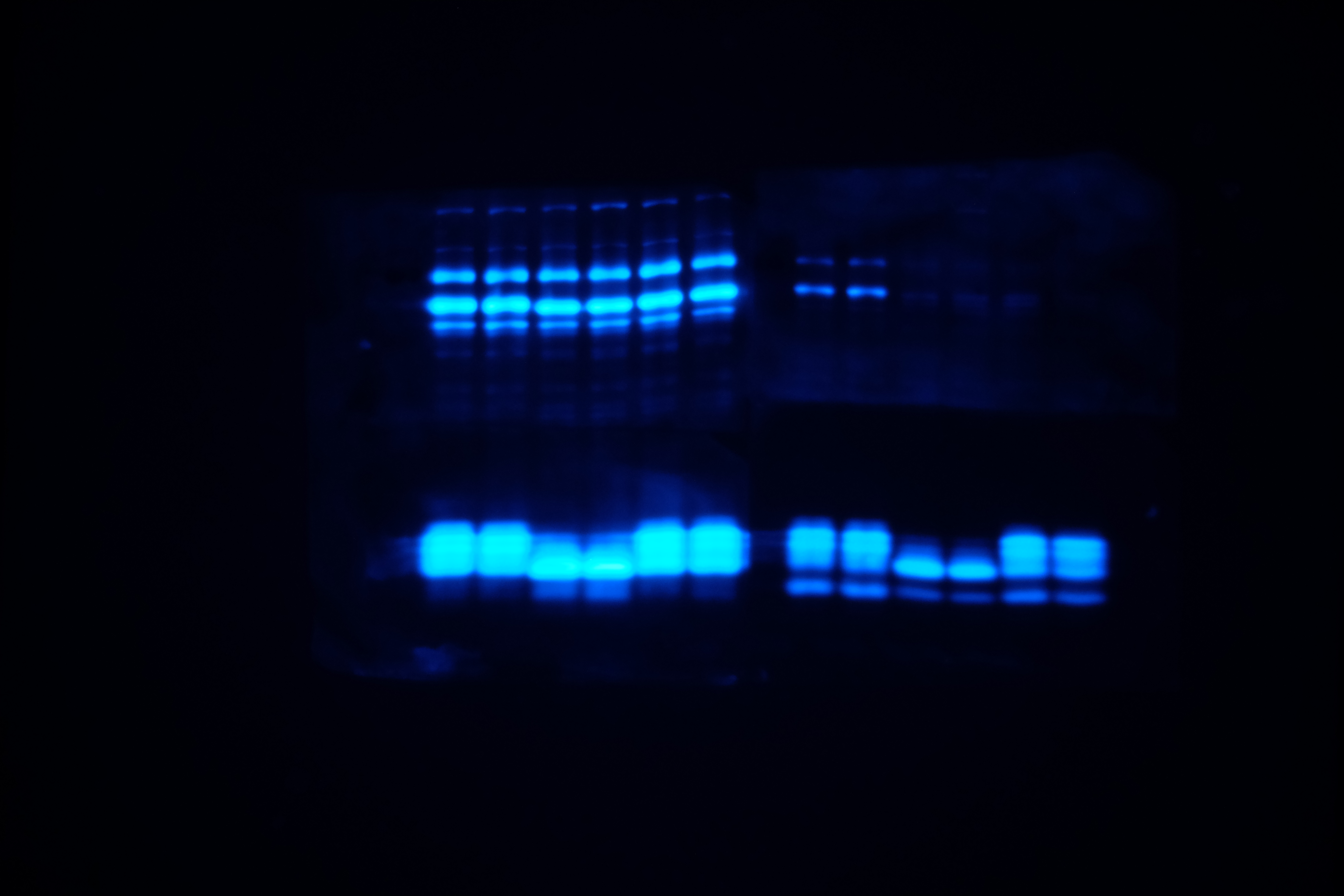

Supplement: Figure 7—source data 2. [file elife-78163-fig7-data2.zip › Figure 7-source data 2/Fig.7B_p-S6K.JPG]

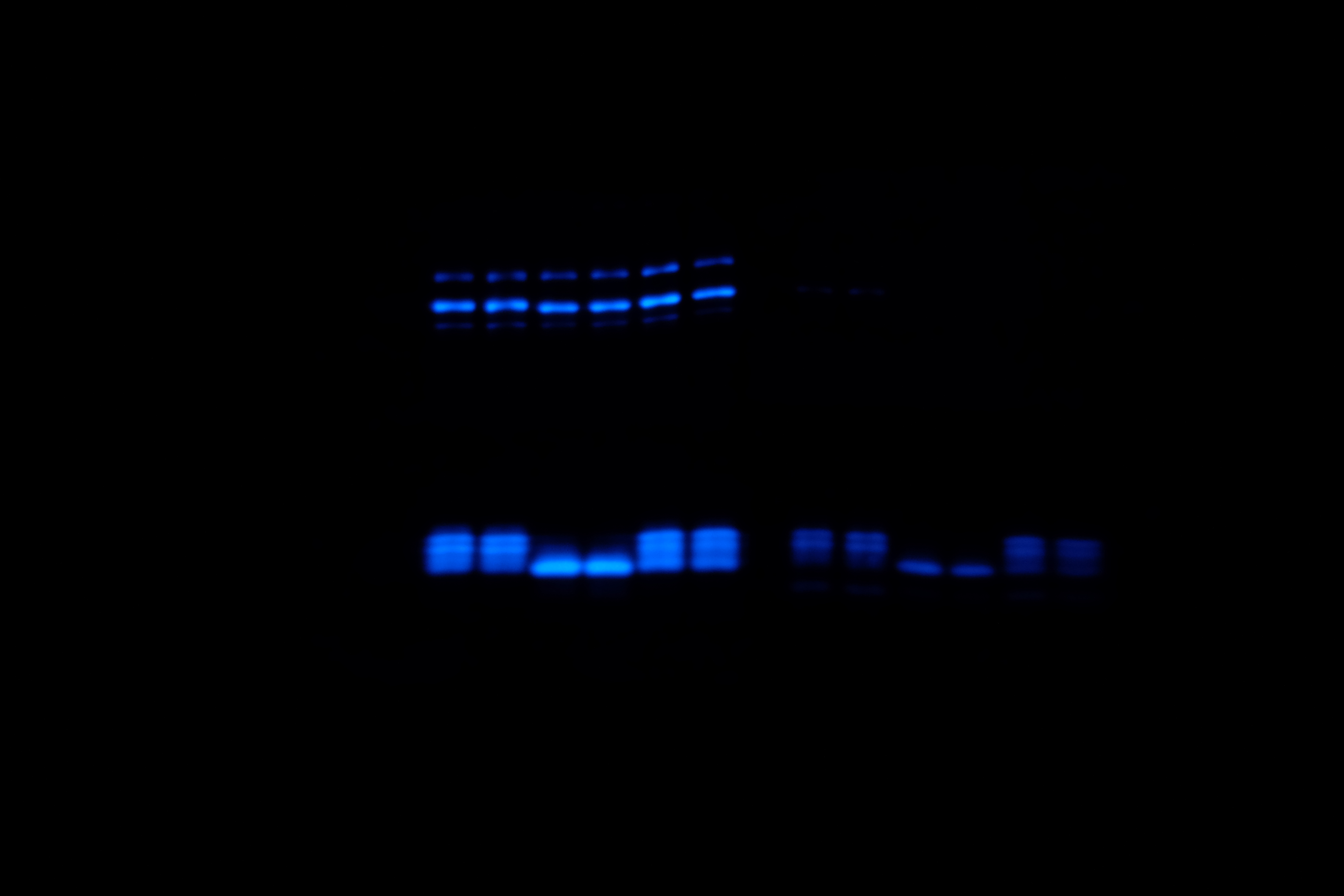

Supplement: Figure 7—source data 2. [file elife-78163-fig7-data2.zip › Figure 7-source data 2/Fig.7B_S6K_p-4E-BP1_4E-BP1.JPG]

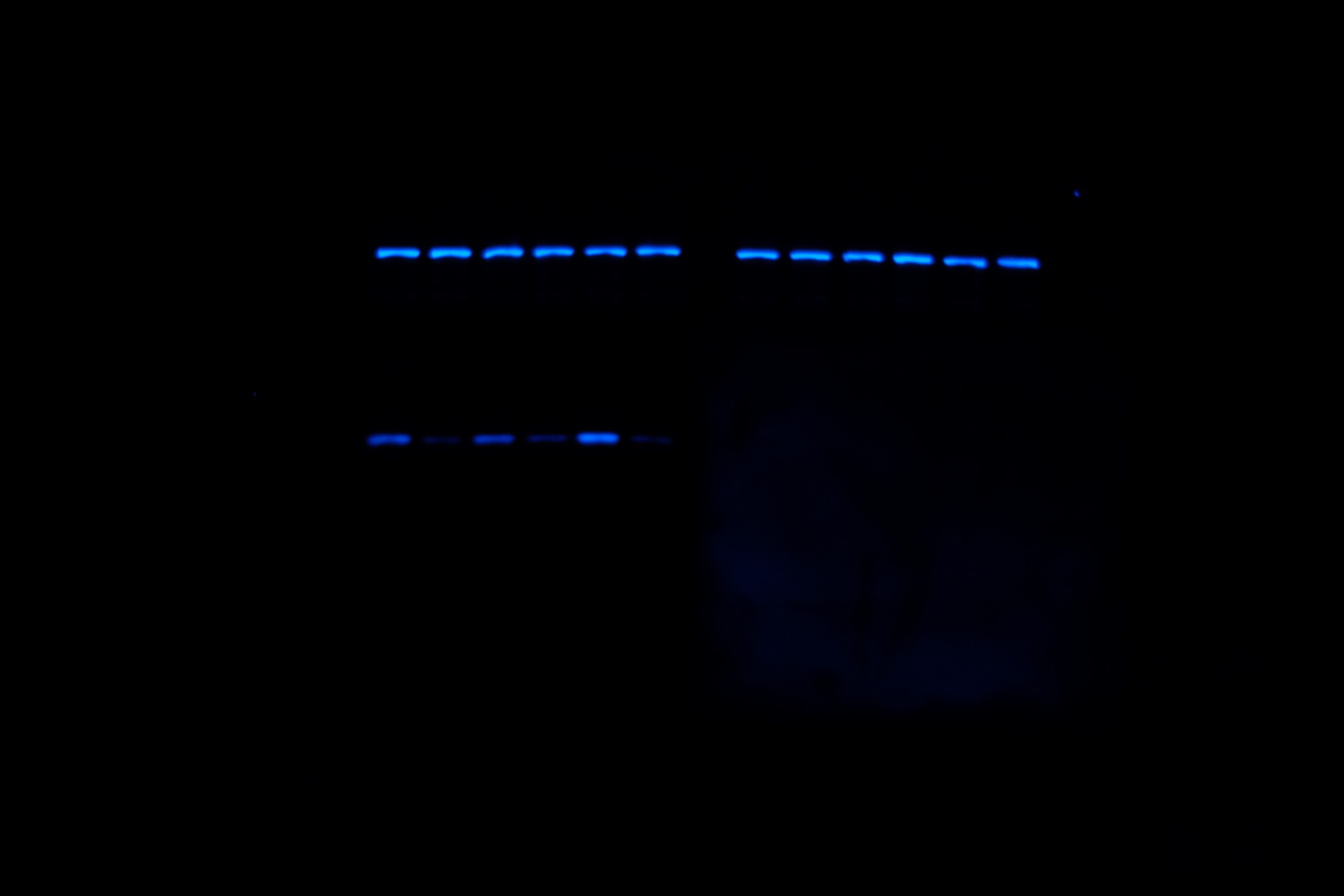

Supplement: Figure 7—source data 2. [file elife-78163-fig7-data2.zip › Figure 7-source data 2/Fig.7B_WDR5_vinculin.JPG]
