# Supplementary material for: Human WDR5 promotes breast cancer growth and metastasis via KMT2-independent translation regulation
Source: eLife. 2022 Aug 31;11:e78163. doi: 10.7554/eLife.78163 (PMC9584608; doi:10.7554/eLife.78163)
Supplement: Figure 2—source data 1. [file elife-78163-fig2-data1.zip › Figure 2-source data 1/Figure 2-source data 1_labeled images.pptx]

## Slide 1
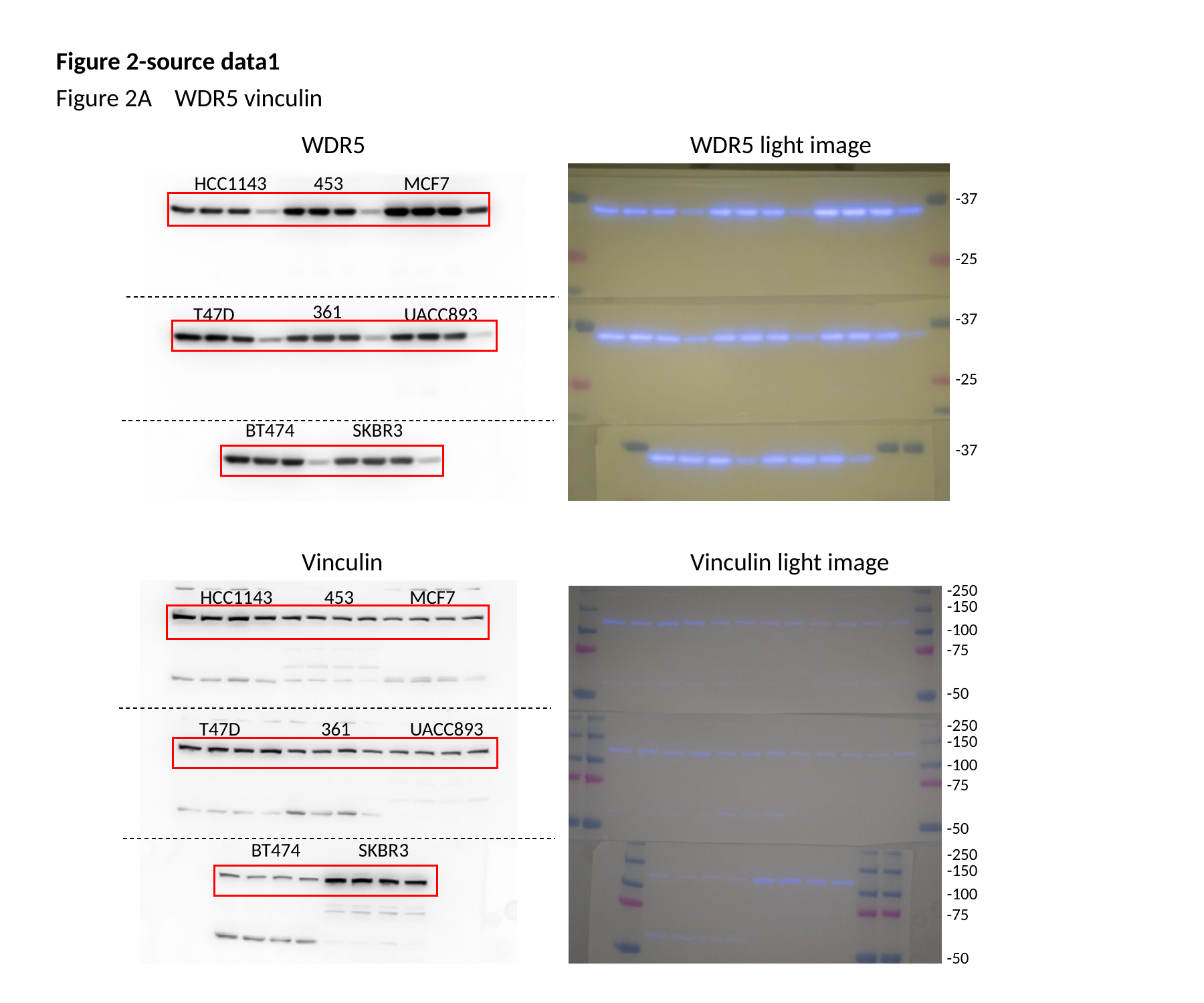

Figure 2-source data1
Figure 2A WDR5 vinculin
WDR5
WDR5 light image
HCC1143
453
MCF7
-37
-25
361
T47D
UACC893
-37
-25
BT474
SKBR3
-37
Vinculin
Vinculin light image
-250
HCC1143
453
MCF7
-150
-100
-75
-50
-250
T47D
361
UACC893
-150
-100
-75
-50
BT474
SKBR3
-250
-150
-100
-75
-50
