# Supplementary material for: Human WDR5 promotes breast cancer growth and metastasis via KMT2-independent translation regulation
Source: eLife. 2022 Aug 31;11:e78163. doi: 10.7554/eLife.78163 (PMC9584608; doi:10.7554/eLife.78163)
Supplement: Figure 2—source data 2. [file elife-78163-fig2-data2.zip › Figure 2-source data 2/Figure 2-source data 2_labeled images.pptx]

## Slide 1
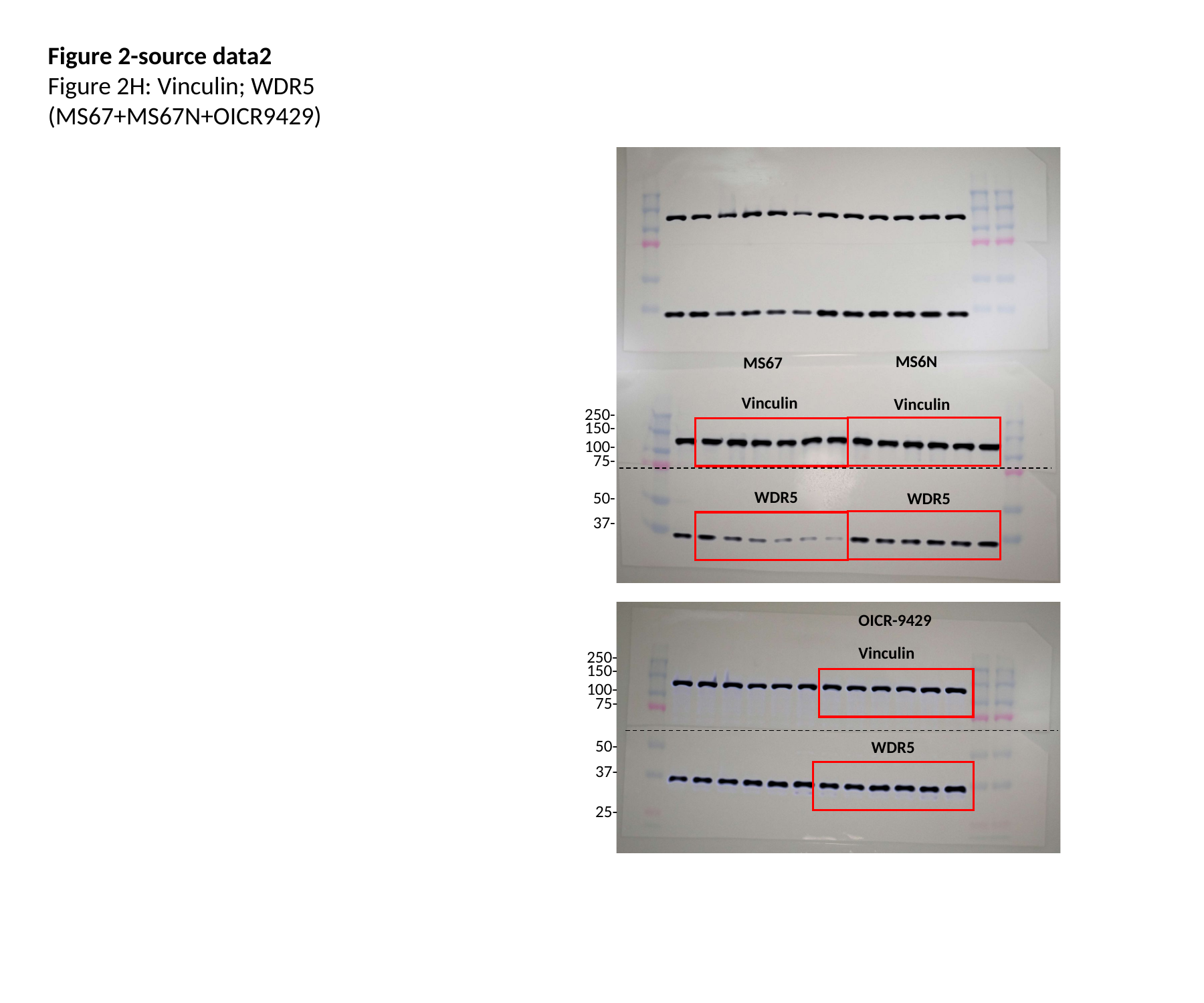

Figure 2-source data2
Figure 2H: Vinculin; WDR5 (MS67+MS67N+OICR9429)
MS6N
MS67
Vinculin
Vinculin
250-
150-
100-
75-
WDR5
50-
WDR5
37-
OICR-9429
Vinculin
250-
150-
100-
75-
50-
WDR5
37-
25-
