# Supplementary material for: Human WDR5 promotes breast cancer growth and metastasis via KMT2-independent translation regulation
Source: eLife. 2022 Aug 31;11:e78163. doi: 10.7554/eLife.78163 (PMC9584608; doi:10.7554/eLife.78163)
Supplement: Figure 2—figure supplement 1—source data 1. [file elife-78163-fig2-figsupp1-data1.zip › Figure 2-figure supplement 1-source data 1/Figure 2-figure supplement 1-source data 1_labeled images.pptx]

## Slide 1
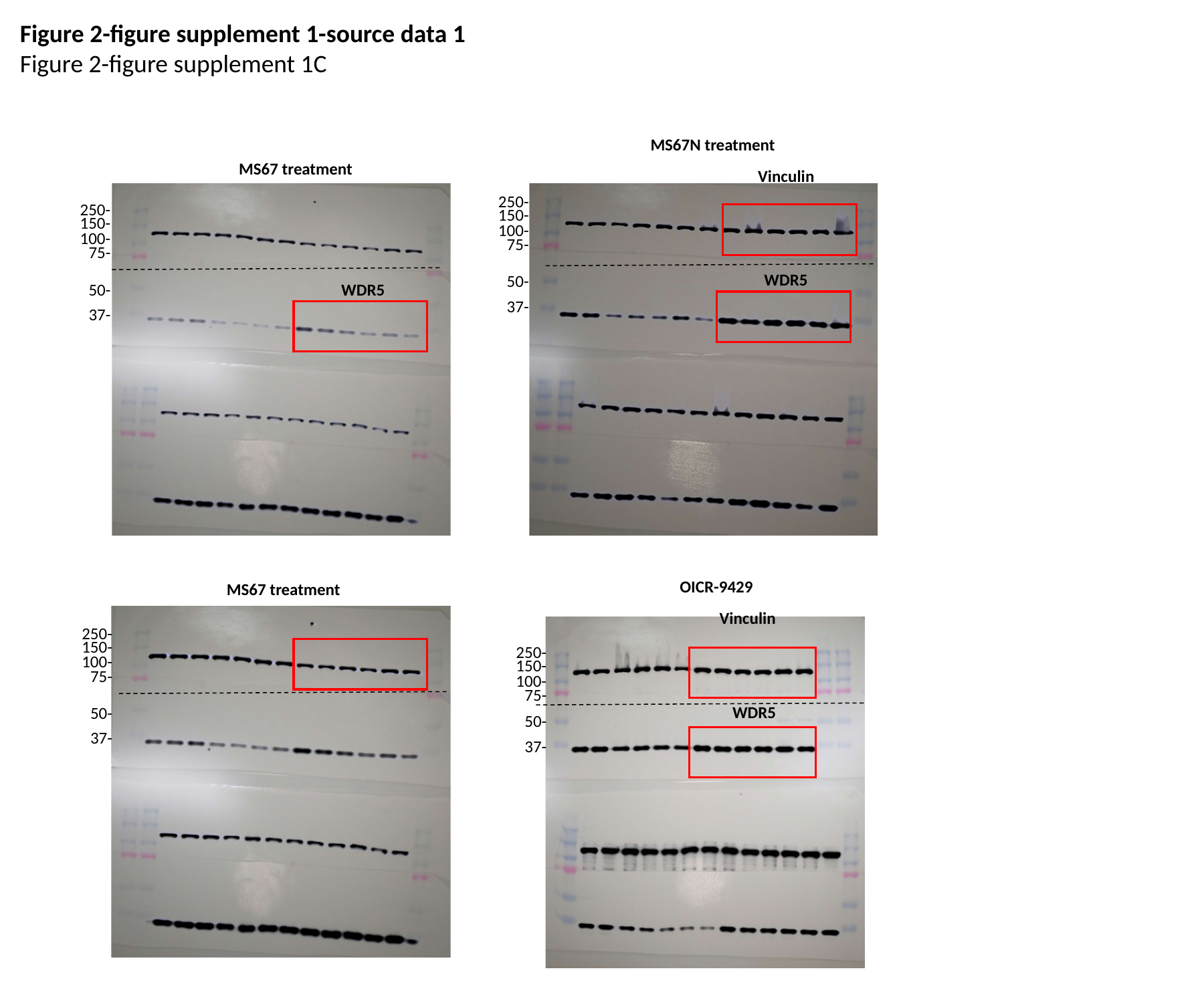

Figure 2-figure supplement 1-source data 1
Figure 2-figure supplement 1C
MS67N treatment
MS67 treatment
Vinculin
250-
250-
150-
150-
100-
100-
75-
75-
WDR5
50-
50-
WDR5
37-
37-
OICR-9429
MS67 treatment
Vinculin
250-
150-
250-
100-
150-
75-
100-
75-
WDR5
50-
50-
37-
37-
