# Supplementary material for: Human WDR5 promotes breast cancer growth and metastasis via KMT2-independent translation regulation
Source: eLife. 2022 Aug 31;11:e78163. doi: 10.7554/eLife.78163 (PMC9584608; doi:10.7554/eLife.78163)
Supplement: Figure 4—source data 2. [file elife-78163-fig4-data2.zip › Figure 4-source data 2/Figure 4-source data 2_labeled images.pptx]

## Slide 1
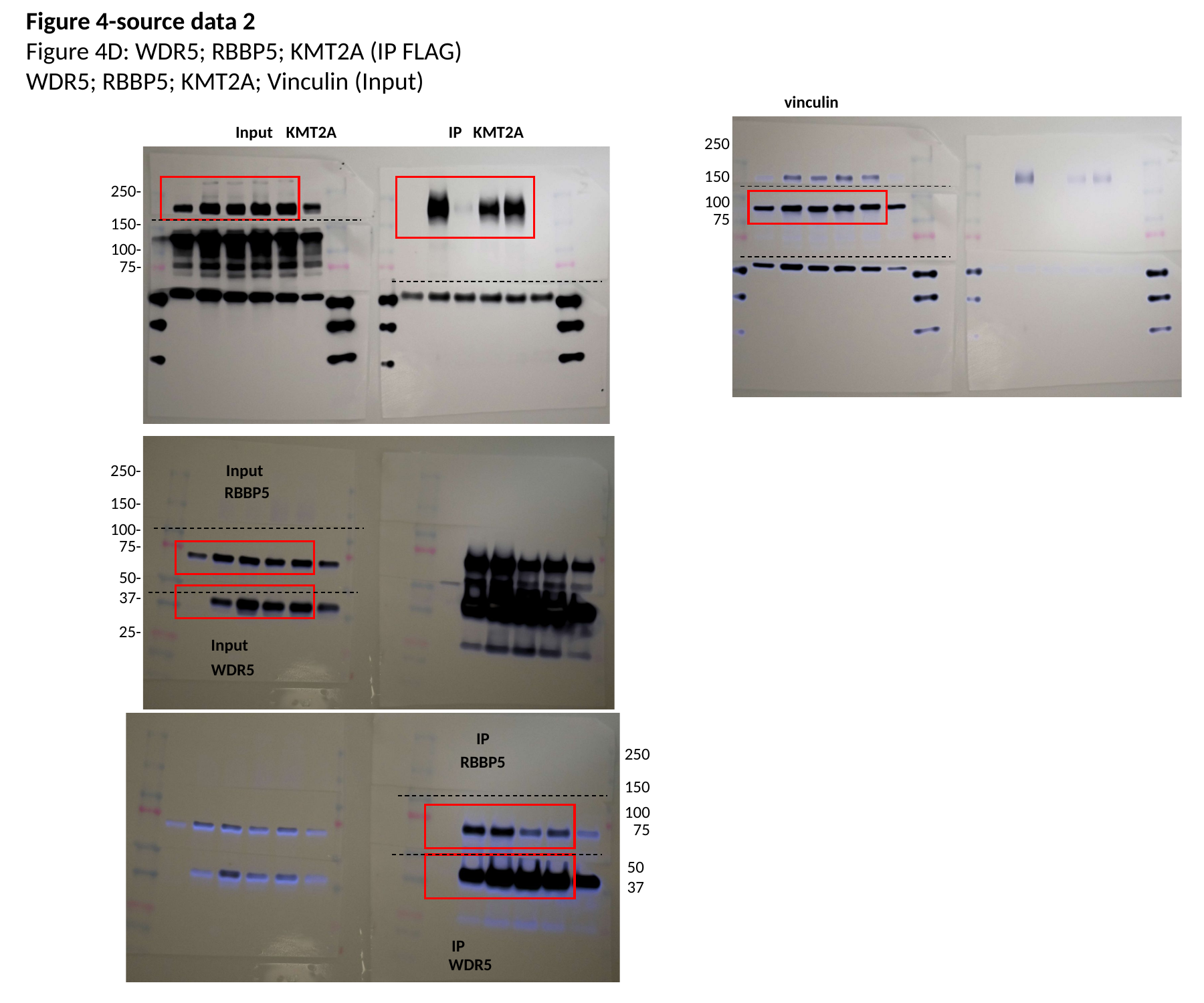

Figure 4-source data 2
Figure 4D: WDR5; RBBP5; KMT2A (IP FLAG) WDR5; RBBP5; KMT2A; Vinculin (Input)
vinculin
KMT2A
KMT2A
Input
IP
250
150
250-
100
75
150-
100-
75-
Input
250-
RBBP5
150-
100-
75-
50-
37-
25-
Input
WDR5
IP
250
RBBP5
150
100
75
50
37
IP
WDR5
