# Supplementary material for: Human WDR5 promotes breast cancer growth and metastasis via KMT2-independent translation regulation
Source: eLife. 2022 Aug 31;11:e78163. doi: 10.7554/eLife.78163 (PMC9584608; doi:10.7554/eLife.78163)
Supplement: Figure 5—source data 1. [file elife-78163-fig5-data1.zip › Figure 5-source data 1/Figure 5-source data 1_labeled images.pptx]

## Slide 1
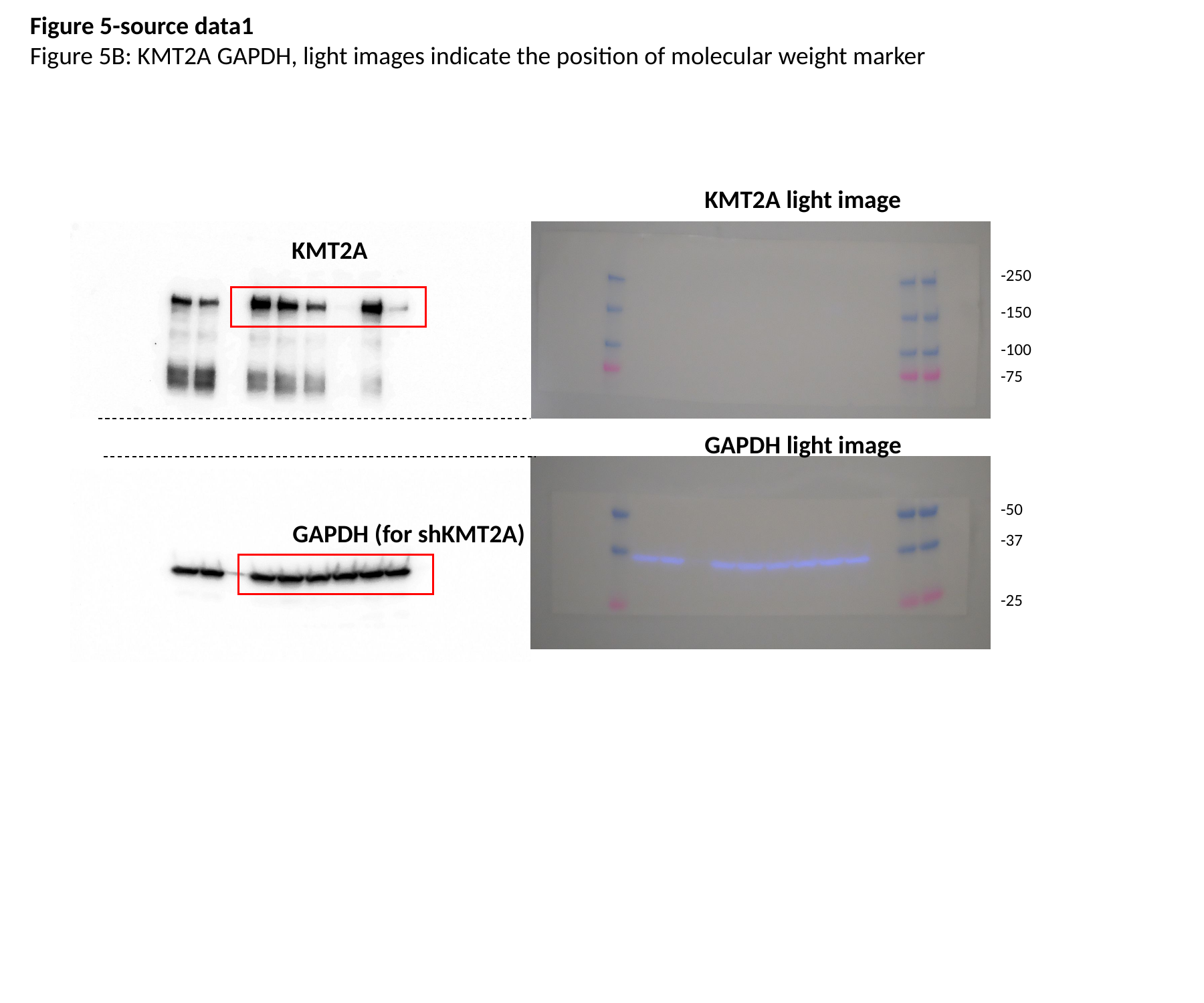

Figure 5-source data1
Figure 5B: KMT2A GAPDH, light images indicate the position of molecular weight marker
KMT2A light image
KMT2A
-250
-150
-100
-75
GAPDH light image
-50
GAPDH (for shKMT2A)
-37
-25

## Slide 2
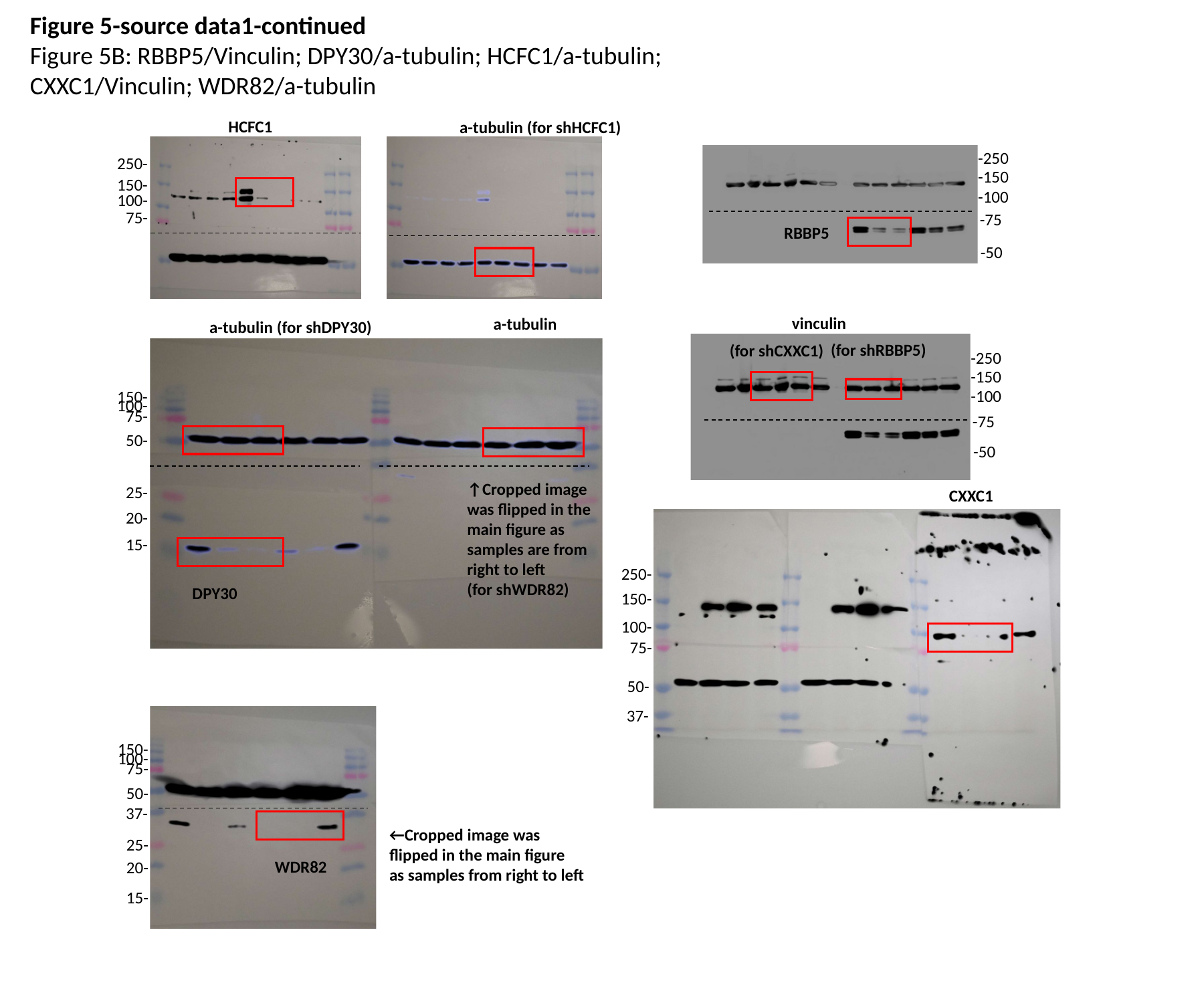

Figure 5-source data1-continued
Figure 5B: RBBP5/Vinculin; DPY30/a-tubulin; HCFC1/a-tubulin; CXXC1/Vinculin; WDR82/a-tubulin
HCFC1
a-tubulin (for shHCFC1)
-250
250-
-150
150-
-100
100-
75-
-75
RBBP5
-50
vinculin
a-tubulin
a-tubulin (for shDPY30)
(for shRBBP5)
(for shCXXC1)
-250
-150
-100
150-
100-
75-
-75
50-
-50
↑Cropped image was flipped in the main figure as samples are from right to left
(for shWDR82)
25-
CXXC1
20-
15-
250-
DPY30
150-
100-
75-
50-
37-
150-
100-
75-
50-
37-
←Cropped image was flipped in the main figure
as samples from right to left
25-
WDR82
20-
15-
