# Supplementary material for: Human WDR5 promotes breast cancer growth and metastasis via KMT2-independent translation regulation
Source: eLife. 2022 Aug 31;11:e78163. doi: 10.7554/eLife.78163 (PMC9584608; doi:10.7554/eLife.78163)
Supplement: Figure 6—source data 1. [file elife-78163-fig6-data1.zip › Figure 6-source data 1/Figure 6-source data 1_labeled images.pptx]

## Slide 1
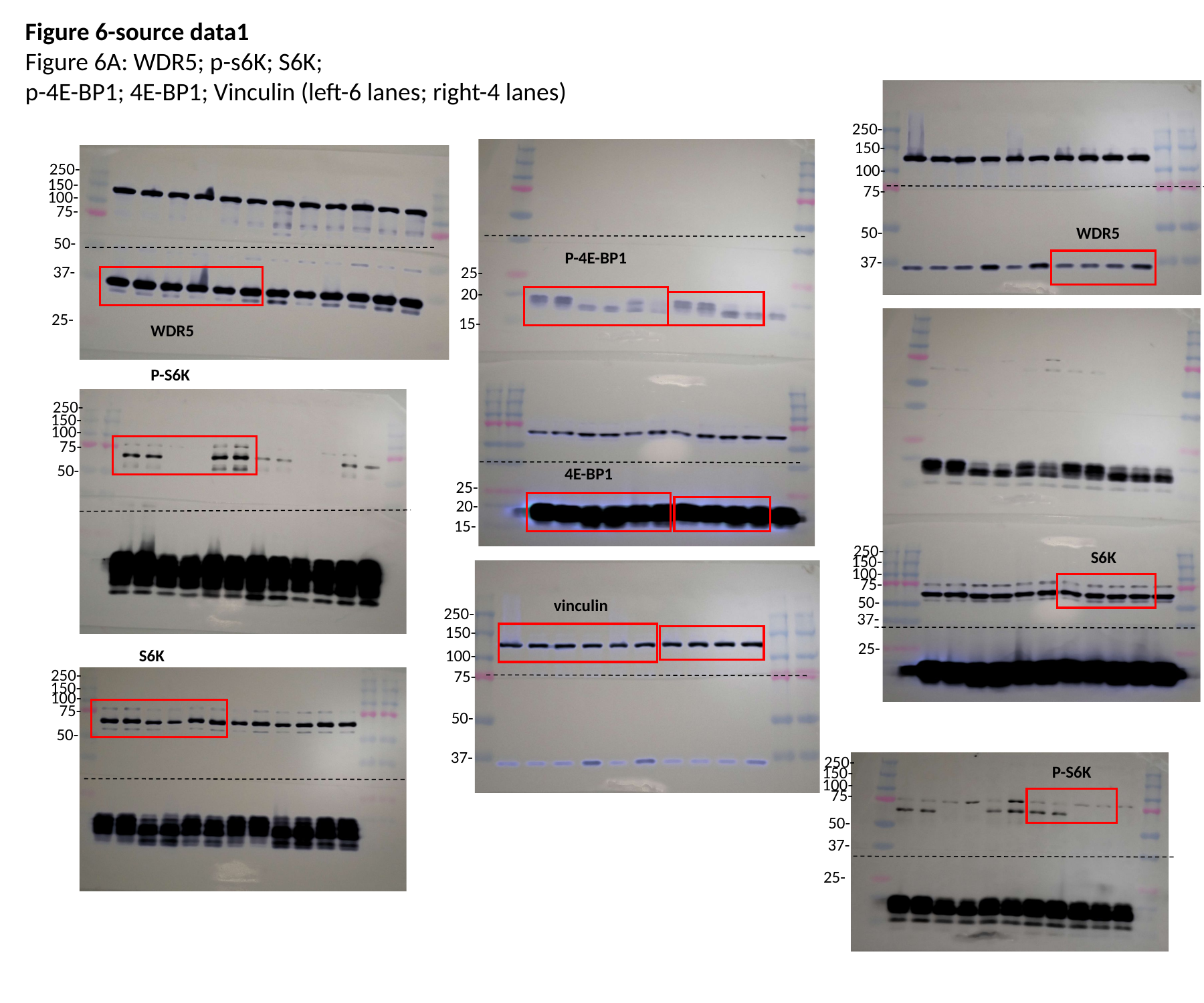

Figure 6-source data1
Figure 6A: WDR5; p-s6K; S6K;
p-4E-BP1; 4E-BP1; Vinculin (left-6 lanes; right-4 lanes)
250-
150-
250-
100-
150-
75-
100-
75-
50-
WDR5
50-
P-4E-BP1
37-
37-
25-
20-
25-
15-
WDR5
P-S6K
250-
150-
100-
75-
50-
4E-BP1
25-
20-
15-
250-
S6K
150-
100-
75-
50-
vinculin
250-
37-
150-
25-
S6K
100-
250-
75-
150-
100-
75-
50-
50-
37-
250-
P-S6K
150-
100-
75-
50-
37-
25-
