# Supplementary material for: Human WDR5 promotes breast cancer growth and metastasis via KMT2-independent translation regulation
Source: eLife. 2022 Aug 31;11:e78163. doi: 10.7554/eLife.78163 (PMC9584608; doi:10.7554/eLife.78163)
Supplement: Figure 6—source data 2. [file elife-78163-fig6-data2.zip › Figure 6-source data 2/Figure 6-source data 2_labeled images.pptx]

## Slide 1
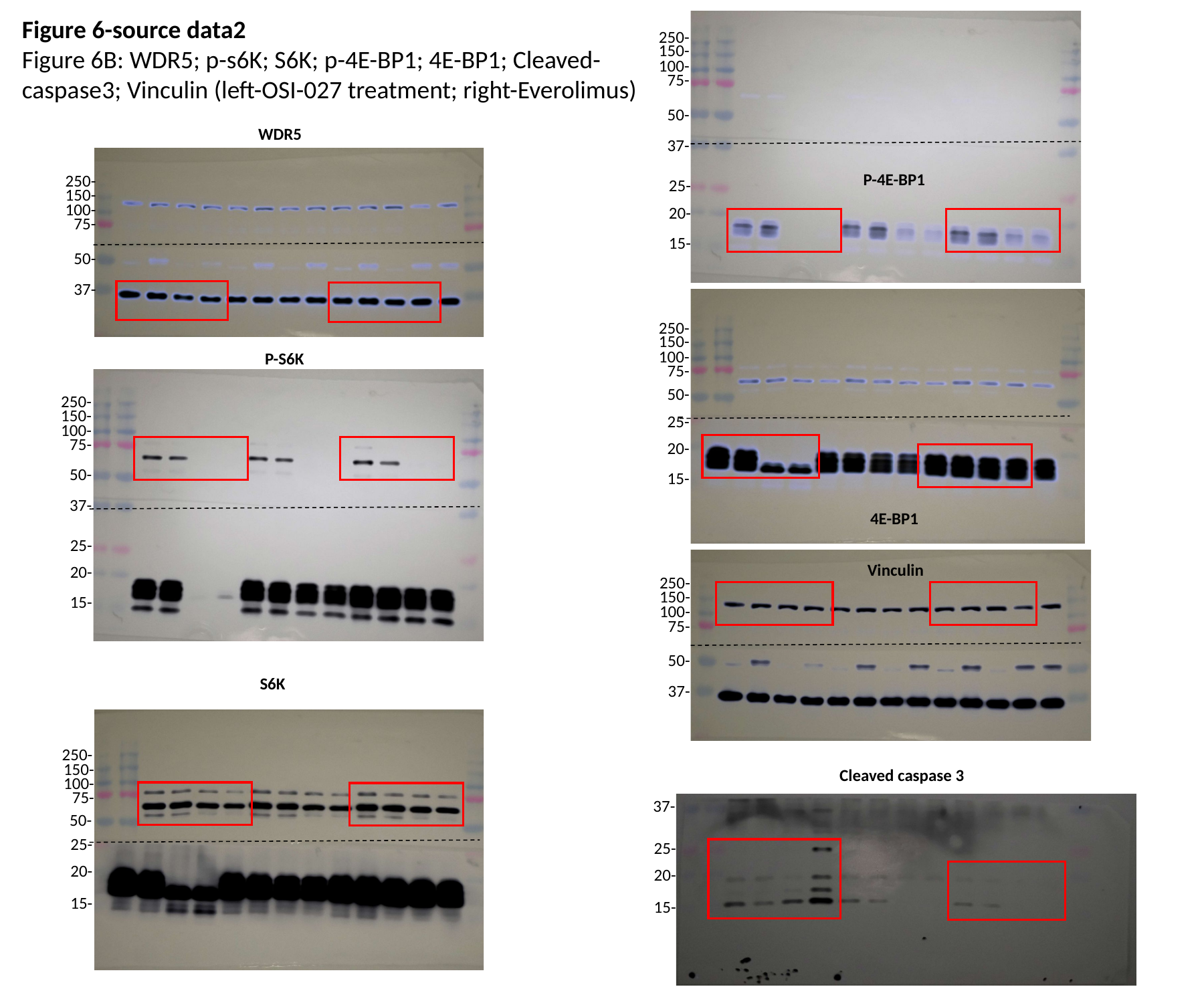

Figure 6-source data2
Figure 6B: WDR5; p-s6K; S6K; p-4E-BP1; 4E-BP1; Cleaved-caspase3; Vinculin (left-OSI-027 treatment; right-Everolimus)
250-
150-
100-
75-
50-
WDR5
37-
P-4E-BP1
250-
25-
150-
100-
20-
75-
15-
50-
37-
250-
150-
100-
P-S6K
75-
50-
250-
150-
25-
100-
75-
20-
50-
15-
37-
4E-BP1
25-
Vinculin
20-
250-
150-
15-
100-
75-
50-
S6K
37-
250-
150-
Cleaved caspase 3
100-
75-
37-
50-
25-
25-
20-
20-
15-
15-
