# Supplementary material for: Human WDR5 promotes breast cancer growth and metastasis via KMT2-independent translation regulation
Source: eLife. 2022 Aug 31;11:e78163. doi: 10.7554/eLife.78163 (PMC9584608; doi:10.7554/eLife.78163)
Supplement: Figure 6—figure supplement 1—source data 1. [file elife-78163-fig6-figsupp1-data1.zip › Figure 6-figure supplement 1-source data 1/Figure 6-figure supplement 1-source data 1_labeled images.pptx]

## Slide 1
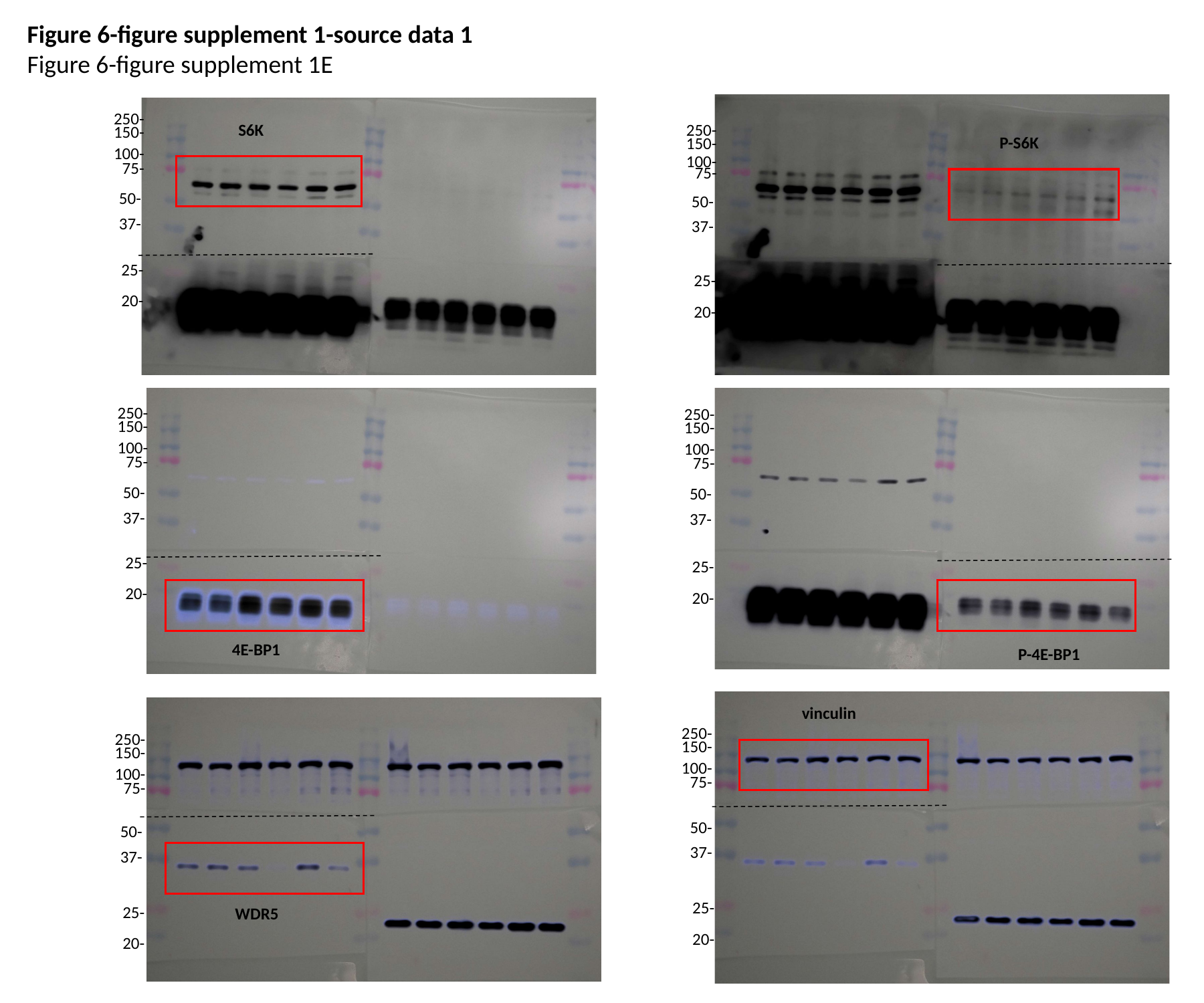

Figure 6-figure supplement 1-source data 1
Figure 6-figure supplement 1E
250-
250-
S6K
150-
P-S6K
150-
100-
100-
75-
75-
50-
50-
37-
37-
25-
25-
20-
20-
250-
250-
150-
150-
100-
100-
75-
75-
50-
50-
37-
37-
25-
25-
20-
20-
4E-BP1
P-4E-BP1
vinculin
250-
250-
150-
150-
100-
100-
75-
75-
50-
50-
37-
37-
25-
25-
WDR5
20-
20-
