# Supplementary material for: Human WDR5 promotes breast cancer growth and metastasis via KMT2-independent translation regulation
Source: eLife. 2022 Aug 31;11:e78163. doi: 10.7554/eLife.78163 (PMC9584608; doi:10.7554/eLife.78163)
Supplement: Figure 6—figure supplement 1—source data 2. [file elife-78163-fig6-figsupp1-data2.zip › Figure 6-figure supplement 1-source data 2/Figure 6-figure supplement 1-source data 2_labeled images.pptx]

## Slide 1
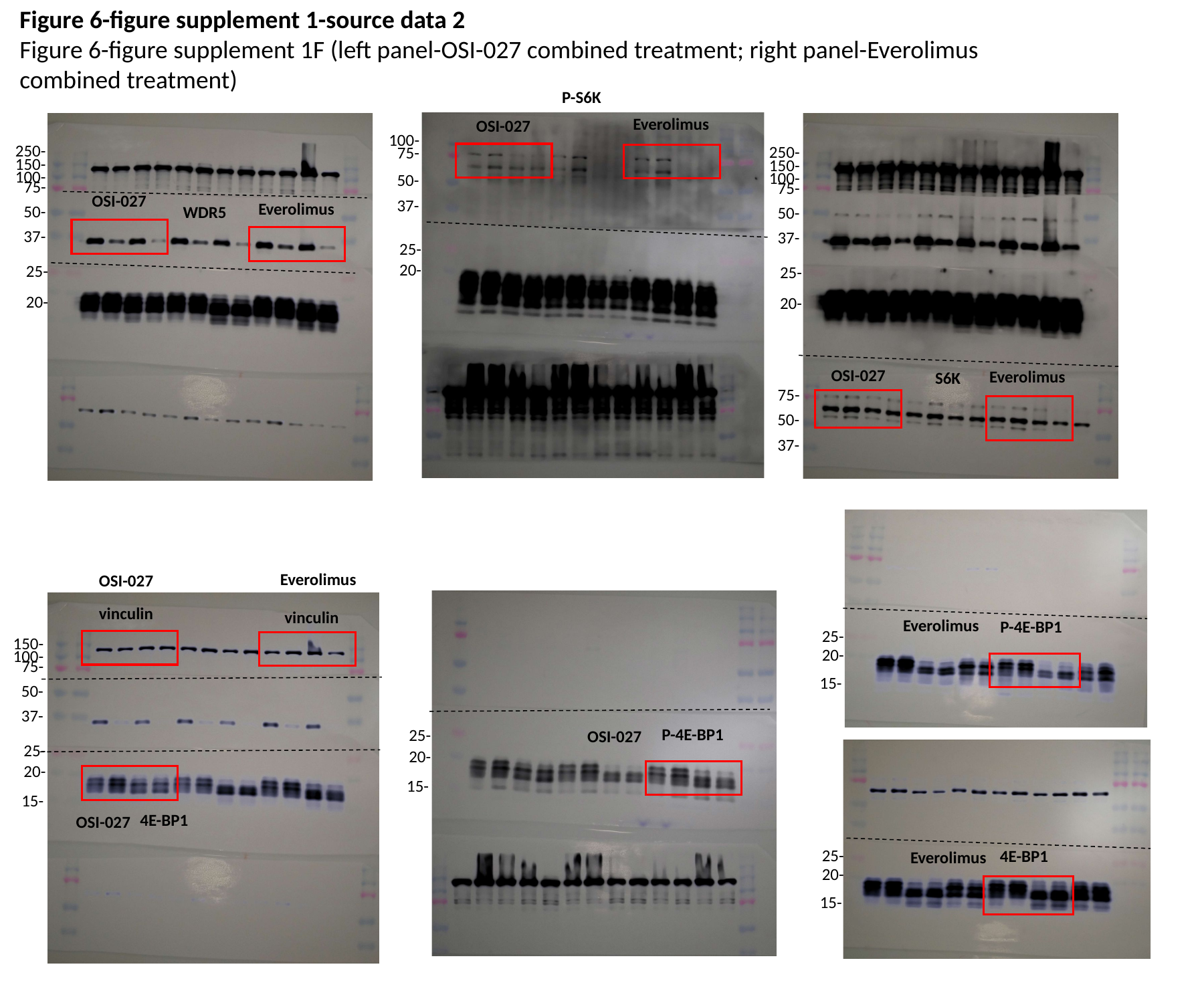

Figure 6-figure supplement 1-source data 2
Figure 6-figure supplement 1F (left panel-OSI-027 combined treatment; right panel-Everolimus combined treatment)
P-S6K
Everolimus
OSI-027
100-
250-
250-
75-
150-
150-
100-
100-
50-
75-
75-
OSI-027
37-
Everolimus
50-
WDR5
50-
37-
37-
25-
20-
25-
25-
20-
20-
OSI-027
Everolimus
S6K
75-
50-
37-
Everolimus
OSI-027
vinculin
vinculin
Everolimus
P-4E-BP1
25-
150-
20-
100-
75-
15-
50-
37-
P-4E-BP1
25-
OSI-027
25-
20-
20-
15-
15-
4E-BP1
OSI-027
25-
4E-BP1
Everolimus
20-
15-
