# Supplementary figures and images for: Human WDR5 promotes breast cancer growth and metastasis via KMT2-independent translation regulation (part 2 of 2)
Source: eLife. 2022 Aug 31;11:e78163. doi: 10.7554/eLife.78163 (PMC9584608; doi:10.7554/eLife.78163)

## Slide 1
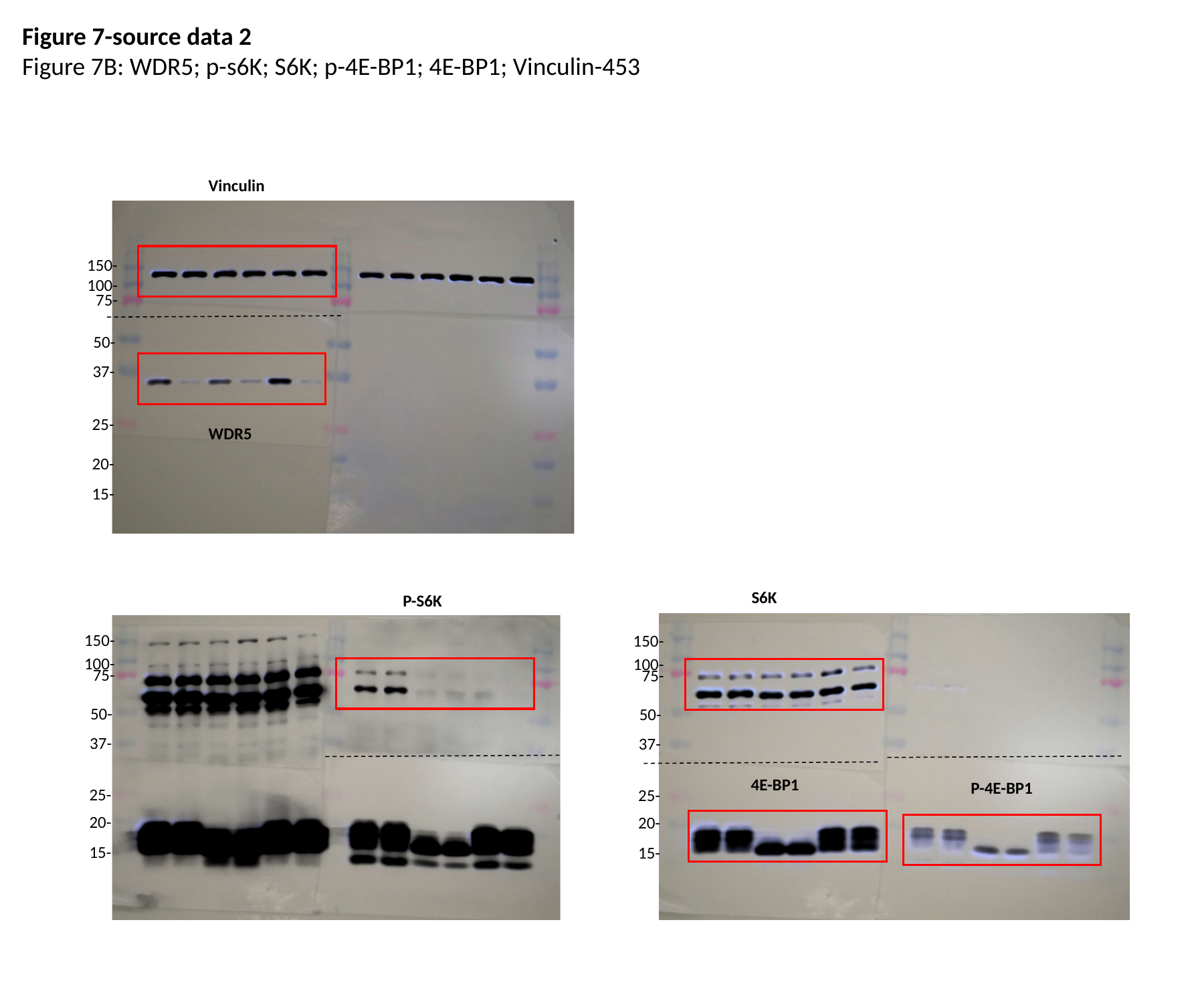

Figure 7-source data 2
Figure 7B: WDR5; p-s6K; S6K; p-4E-BP1; 4E-BP1; Vinculin-453
Vinculin
150-
100-
75-
50-
37-
25-
WDR5
20-
15-
S6K
P-S6K
150-
150-
100-
100-
75-
75-
50-
50-
37-
37-
4E-BP1
P-4E-BP1
25-
25-
20-
20-
15-
15-

Supplement: Figure 7—source data 2. [file elife-78163-fig7-data2.zip › Figure 7-source data 2/Figure 7-source data 2_labeled images.pptx]
